# Shao-Ma-Zhi-Jing granules alleviate Tourette Syndrome by modulating the cAMP/PI3K/AKT/NF-κB signaling pathway, T cell differentiation, microglia, and gut microbiota

**DOI:** 10.3389/fphys.2026.1827824

**Published:** 2026-06-17

**Authors:** Xin Xue, Lin-Ying Luo, Jing Wang, Rui Yang, Xiao-Hui Ma, Gen-Bei Wang, Zhao-Hui Song, Xin-Xin Li

**Affiliations:** 1 School of Traditional Chinese Pharmacy, China Pharmaceutical University, Nanjing, China; 2 National Key Laboratory of Chinese Medicine Modernization, Tasly Research Academy, Tasly Holding Group Co, Ltd., Tianjin, China

**Keywords:** immune mechanism, intestinal flora, microbial-gut-brain axis, Shao-Ma-Zhi-Jing granules, Tourette syndrome

## Abstract

**Introduction:**

Shao-Ma-Zhi-Jing Granules (SMZJ) is clinically used for Tourette syndrome (TS). This study aimed to investigate the regulatory effects of SMZJ on the immune system and gut microbiota of the 3,3’-iminodipropionitrile (IDPN)-induced TS rat model.

**Methods:**

Immune-related indicators were detected by flow cytometry, western blotting, ELISA and qRT-PCR. Gut microbiota composition was analyzed via 16S rDNA sequencing, and Spearman analysis was performed to assess the correlation between gut microbiota and immune function.

**Results:**

SMZJ suppressed IDPN-induced stereotypical behaviors. Furthermore, SMZJ regulated the mRNA and protein levels of T helper 1 (Th1), Th2, Th17, and T regulatory (Treg) cell-related transcription factors and modulated Th1 and Th2 cytokines in the TS rat model. SMZJ downregulated the inflammatory pathway in the brain striatum, the TNF-α and IL-6 (inflammatory cytokines) levels, and the striatal iNOS levels in the TS rat model. The diversity and structure of the gut microbiota were remodeled using the SMZJ-treated TS rat model. SMZJ modulated the correlation between immune parameters and gut microbiota.

**Conclusions:**

SMZJ alleviated peripheral and neuroinflammation in TS rats via promoting T cell differentiation and downregulating TNF-α and IL-6 levels, which may be related to changes in the cAMP/PI3K/Akt/NF-κB signaling pathway. SMZJ downregulated the activation of striatal M1 microglia in the TS model. SMZJ may potentially modulated gut microbiota structure and metabolism, relieves peripheral and neuroinflammation, and ameliorates intestinal mucosal barrier injury.

## Graphical Abstract

**Figure:**
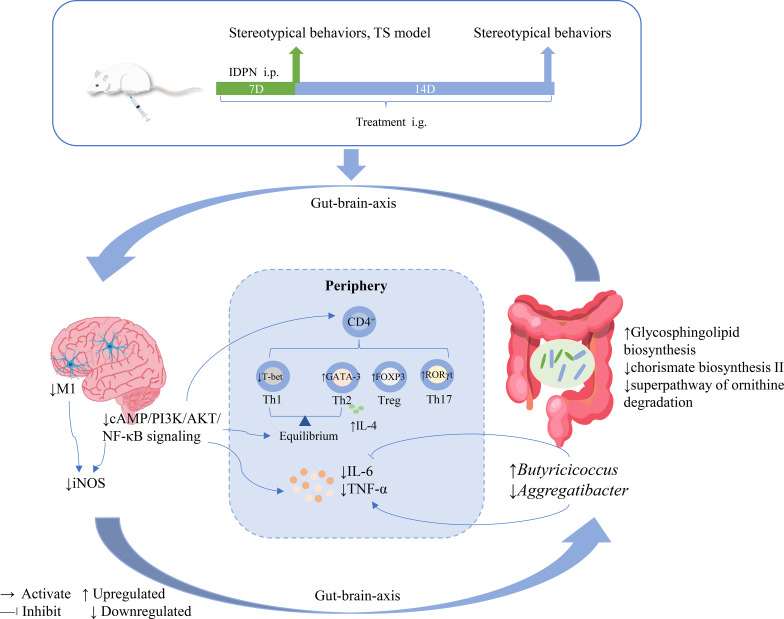
Some materials in the graphical abstract are derived from smartSERVIER MEDICAL ART (https://smart.servier.com/), licensed under CC BY 4.0 (https://creativecommons.org/licenses/by/4.0/).

## 1 Introduction

Tourette syndrome (TS), a complex neurodevelopmental disorder, is characterized by the presence of motor tics, vocal tics, or both. Globally, the prevalence rate of TS in children is in the range of 0.3%–1% (Scharf et al., 2015). A prospective clinical study revealed that the tics relieve in only 17% of patients with TS by the age of 16 years and persist to varying degrees in 83% of patients (Groth et al., 2017). The development of TS is associated with the dysfunction of various neurotransmitter systems, especially the dopaminergic (DA) system (Set and Warner, 2021). Recent studies examining the pathogenesis of TS have focused on neurotransmitters, immune mechanisms, and gut microbiota. Immune dysfunction in TS manifests as changes in various circumgyration immune indices of peripheral immunity and neuroimmunity (Hsu et al., 2021).

The human gut contains the most complex microbiota, which critically regulates host homeostasis and immunostasis and is essential for health. The gut microbiota modulates immune activity and cytokine secretion through crosstalk with gut-associated lymphoid tissues (Lazar et al., 2018). Concurrently, the intestinal microbiota is critical for maintaining intestinal barrier integrity, including mucus secretion and tight junction stability (Rolhion and Chassaing, 2016). The gut microbiota, immune system, and central nervous system (CNS) form a mutually regulatory network known as the gut–brain axis (Rutsch et al., 2020). The bi-directional transmission of immune signals can occur between the gut and the CNS through the humoral, cellular immune, and neuronal pathways (Agirman et al., 2021). Dysbiosis leads to intestinal barrier disruption and may trigger elevated pro-inflammatory immune responses, while inflammatory cytokines further increase intestinal permeability and aggravate barrier dysfunction, forming a vicious cycle (Takiishi et al., 2017). The systemic upregulation of pro-inflammatory cytokines promotes inflammatory signaling in the brain microglia (Parker et al., 2020). In addition to releasing inflammatory factors, the gut microbiota and its byproducts can exert neuroimmunomodulatory effects in the CNS. Gut microbiota also directly affects microglial maturation and function in the brain (Erny et al., 2015). The presence of particular gut microbiota changes in TS provides a new idea for its treatment. Hence, fecal microbial transplantation has been suggested for the treatment of TS (Geng et al., 2023).

Shao-Ma-Zhi-Jing Granules (SMZJ) is a Class I innovative traditional Chinese medicine approved for marketing in 2019, possessing official and standardized quality control criteria (Liao et al., 2023). This modern compound herbal preparation comprising the following 11 herbs: *Rhizoma Gastrodiae* (Tian Ma), *Uncariae Ramulus Cum Uncis* (Gou Teng), *Paeoniae Radix Alba* (Bai Shao), *Gardeniae fructus* (Zhi Zi), *Scutellariae radix* (Huang Qin), *Ganoderma Lucidum* (Ling Zhi), *Tribuli Fructus* (Ji Li), *Schisandrae Chinensis Fructus* (Wu Weizi), *Polygoni Multiflori Caulis* (Shou Wuteng), *Ziziphi Spinosae Semen* (Suan Zaoren), and *Arisaema Cum Bile* (Dan Nanxing). Our group previously performed ultra-performance liquid chromatography with quadrupole time-of-flight mass spectrometry to examine the plasma and urinary levels of SMZJ components. A total of 45 components were identified, mainly including flavonoids, cyclic enol ether terpene glycosides, and alkaloids, which were primarily derived from *Rhizoma Gastrodiae* (Tian Ma), *Paeoniae Radix Alba* (Bai Shao), *Uncariae Ramulus Cum Uncis* (Gou Teng), *Gardeniae fructus* (Zhi Zi), and *Scutellariae radix* (Huang Qin) (Wei et al., 2019). SMZJ granules are clinically used to treat TS and chronic tic disorders. The results of a phase III clinical study revealed that the effective rate of SMZJ was 74%, which was significantly higher than that of the placebo (Zheng et al., 2016). The overall incidence of adverse events in the SMZJ group was significantly lower than that in the tiapride group. The regulatory effects of SMZJ on the immune system and gut flora in TS have not been previously examined.

This study hypothesized that SMZJ alleviates TS by modulating immunity and gut microbiota structure in rats. Thus, this study evaluated the regulatory effects of SMZJ on peripheral immune and neuroimmune indices in the 3,3’-iminodipropionitrile (IDPN)-induced TS rat model. Additionally, the effects of SMZJ on intestinal dysbiosis in the TS rat model were examined. Furthermore, the correlation between gut flora and immune indicators was analyzed.

## 2 Materials and methods

### 2.1 Drugs and reagents

The following materials and reagents were obtained from different vendors: SMZJ (Batch No.: 20220101, Tasly Pharmaceutical Group Co., Ltd., Tianjin, China); IDPN (SIGMA, St. Louis, MO, USA); interleukin 4 (IL-4), IL-6, tumor necrosis factor (TNF-α), and interferon gamma (IFN-γ) enzyme-linked immunosorbent assay (ELISA) kits (Enzyme-linked Biotechnology Co., Ltd., Shanghai, China); allophycocyanin (APC)-conjugated anti-CD3 monoclonal antibody (eBioG4.18 (G4.18), fluorescein isothiocyanate (FITC)-conjugated anti-CD4 monoclonal antibody (OX35), and phycoerythrin (PE)-conjugated anti-CD8a monoclonal antibody (OX8) (Thermo Fisher Scientific Inc. Waltham, MA, USA); anti-cyclic adenosine monophosphate (cAMP), anti-p-AKT, anti-IBA-1 antibodies (Abcam Plc. Cambridge, MA, USA); anti-phosphatidylinositol-3-kinase (PI3K) and anti-NF-κB antibodies (Cell Signaling Technology, Inc. Danvers, MA, USA); anti-AKT and anti-iNOS antibodies (Proteintech Group, Inc. Chicago, IL, USA); anti-p-p65, anti-FOXP3, and anti-T-bet antibodies (Beijing Biosynthesis Biotechnology Co., Ltd., Beijing, China); anti-RORγt antibody (Boster Biological Technology Co., Ltd., CA, USA); anti-GATA-3 antibody (HUABIO Co., Ltd., Hangzhou, China); anti-β-tubulin (ZSGB-BIO Co., Ltd., Beijing, China).

### 2.2 Animals

Male Sprague Dawley rats (bodyweight: 200 g ± 20 g) were procured from Beijing Vital River Laboratory Animal Technology Co., Ltd, (Beijing, China, Animal certificate: SCXK [Beijing] 2012–0001, License No. 110011230102383475). All rats were housed in an air-conditioned animal room under the following conditions: circadian cycle, 12 h light/dark cycle; temperature, 23 °C ± 2 °C; humidity, 50% ± 10%. The animal experiments were approved by the Ethics Review Committee of Tasly (approval number: TSL-IACUC-2023-11).

### 2.3 Grouping and drug administration

After conventional feeding for 3–4 days, the rats were randomly divided into the following five groups (n=10) (Xue et al., 2025): control (Ctrl) group, intraperitoneally administered with normal saline and orally administered with distilled water at a volume of 10mL/kg; model group, intraperitoneally administered with IDPN (SIGMA, St. Louis, MO, USA) at a dose of 150 mg/kg bodyweight/day for 7 days and orally administered with distilled water at a volume of 10mL/kg (Liu et al., 2021); haloperidol (Hal) group, treated with oral haloperidol (Ningbo Dahongying Pharmaceutical Co., Ltd., Zhejiang, China) at a dose of 105 µg/kg bodyweight after modelling with IDPN; low-dose SMZJ (SML) group, gavaged with SMZJ (Tasly Pharmaceutical Group Co., Ltd., Tianjin, China) at a dose of 1.575 g/kg bodyweight after modelling with IDPN; high-dose SMZJ (SMH) group, gavaged with SMZJ at a dose of 6.3 g/kg bodyweight after modelling with IDPN (Xue et al., 2025). The experimental period was 21 days.

### 2.4 Behavioral observations

The rats were placed in the observation cage (a quiet indoor environment without light interference) and observed after 5 min of adaptation. Each observation lasted for 2–3 min. Scoring was performed using the double-blind method. The scores determined by two evaluators were averaged. The stereotyped behavior scoring criteria were scored using the criteria proposed by Diamond BI (Lin et al., 2019) (**Table 1**). The number of times the animals exhibited head raising and circumgyration behaviors in 5 min was recorded on days 7 and 21.

**Table 1:** Stereotyped behavior scale.

Scores	Stereotyped behaviors
0	No stereotypical movement
1	Circumgyration behavior (clockwise or counterclockwise rotational motion)
2	Excessive vertical head and neck movements (stereotypic head raising)
3	Excessive vertical head and neck movements plus circumgyration behavior;
4	Head swings to the side, in combination excessive vertical head and neck movements

### 2.5 Thymus and spleen indices

Each animal was sacrificed after recording the bodyweight, collecting peripheral blood samples, and removing the spleen and thymus. The fresh weight of the excised spleen and thymus was recorded. The spleen and thymus indices were determined by dividing their weight by the bodyweight.

### 2.6 Proportions of T cell subset

CD3^+^, CD4^+^, and CD8^+^ T cells in the blood samples were detected using flow cytometry with the APC-conjugated anti-CD3 monoclonal antibody, FITC-conjugated anti-CD4 monoclonal antibody, and PE-conjugated anti-CD8a monoclonal antibody. The data were collected and evaluated using ACEA NovoCyte (Agilent Technologies Inc. Palo Alto, CA, USA) and NovoExpress 1.4.1 (Agilent Technologies Inc. Palo Alto, CA, USA).

### 2.7 Western blotting

Total proteins were extracted from the spleen and brain striatum tissues using radioimmunoprecipitation assay lysis buffer (Beyotime Biotechnology, Shanghai, China). The protein concentration was quantified using the bicinchoninic acid protein assay kit (Biosharp, Beijing, China). Based on the molecular weight of the proteins, the protein samples were resolved using a 10% separated or a 4% concentrated gel. The resolved proteins were blotted onto a polyvinylidene difluoride membrane. The membrane was blocked with a blocking solution (Beyotime Biotechnology, Shanghai, China) at room temperature with slow shaking on a shaker for 1 h. Next, the membrane was probed with the anti-cAMP (1:50000), anti-AKT (1:25000), anti-β-tubulin (1:2000), anti-iNOS (1:2000), anti-PI3K, anti-p-AKT, anti-p-p65, anti-p65, anti-IBA-1, anti-FOXP3, anti-RORγt, anti-T-bet (all 1:1000), and anti-GATA-3 (1:700) antibodies at 4 °C overnight, followed by incubation with the secondary antibody (Goat Anti-Mouse IgG, ZB-2305) for 1 h. Immunoreactive signals were developed using an enhanced chemiluminescence reagent (Biosharp, Beijing, China) and visualized using Champchemi 610 plus (SinSage Technology Co., Ltd., Beijing, China). Densitometric analysis of immunoreactive bands was performed using the ImageJ software (Gao et al., 2024).

### 2.8 Quantitative real-time polymerase chain reaction (qRT-PCR) analysis

Total RNA was extracted from the rat spleen tissues using TRIZOL reagent (Thermo Fisher Scientific Inc. Waltham, MA, USA). The isolated RNA was subjected to reverse transcription to obtain complementary DNA (cDNA). The reaction conditions were as follows: oligo (dT) primer, dNTP mixture, RNA template, and diethyl pyrocarbonate (DEPC)-treated water incubated at 65 °C for 5 min, followed by freezing for 10 min and incubation with Moloney murine leukemia virus (M-MLV) reverse transcriptase, 5× M-MLV buffer, RNase inhibitor, and DEPC-treated water at 42 °C for 60 min. The mRNA levels of *Foxp3*, *Rorγt*, *T-bet*, and *Gata-3* in the spleen tissues were determined using qRT-PCR analysis. The qRT-PCR mixture comprised Ultra SYBR mixture (low rox) (Cowin Biotech Co., Ltd., Beijing, China), forward primer, reverse primer, cDNA template, and double distilled water. The primer sequences for *Foxp3*, *Rorγt*, *T-bet*, and *Gata-3* are listed in **Table 2**. The expression of genes was determined using the 2^−ΔΔCt^ method.

**Table 2:** Primer sequences used in quantitative real-time polymerase chain reaction (qRT-PCR) analysis.

Genes	Forward primer (5’-3’)	Reverse primer (5’-3’)
*T-bet*	GTGAATGACGGTGAGCCAGAG	GGTAGGCAGTCACGGCAATG
*Gata-3*	CCCTTATCAAGCCCAAGCGAAG	GTCTCCGTTAGCGTTCCTCCT
*Rorγt*	TCTGGAAGCTGTGGGATAGA	GAGGAGCCTGTGGAGAAATAC
*Foxp3*	ACGCATGTTCGCCTACTTCAG	TCTCACTCTCCACTCGCACAA
*β-actin*	CGCGAGTACAACCTTCTTGC	ATACCCACCATCACACCCTG

### 2.9 ELISA

The serum levels of IL-4, IL-6, TNF-α, and IFN-γ were determined using the ELISA detection kits, following the manufacturer’s instructions.

### 2.10 Analysis of gut microbiota using 16S rRNA sequencing

The microbial DNA was extracted from the rat cecal samples using an OMEGA soil DNA kit (Omega Bio-Tek, Norcross, GA, USA). The V3-V4 region of the bacterial 16S rDNA was amplified by PCR using primers 338F (ACTCCTACGGGAGGCAGCA) and 806R (GGACTACHVGGGTWTCTAAT). The amplicons were purified with Vazyme VAHTSTM DNA clean beads (Vazyme, Nanjing. China) and quantified using the Quant-iT PicoGreen dSDNA assay kit (Invitrogen, Carlsbad, CA, USA). After the individual quantification, amplicons were pooled in equal amounts. Sequencing libraries were prepared using the Illumina TruSeq Nano DNA LT Library Prep Kit (Illumina, San Diego, CA, USA). Paired-end 2 × 250 bp sequencing was performed on the Illumina NovaSeq 6000 platform (Illumina, San Diego, CA, USA) with the NovaSeq 6000 SP Reagent Kit (500 cycles) at Shanghai Metabo-Profile Biotechnology Co., Ltd (Shanghai, China). The raw sequence data were demultiplexed using the demux plugin (QIIME2 2019.4), and the primer sequences were removed using the cutadapt plugin (QIIME2 2019.4). Quality filtering was performed using the DADA2 plugin (QIIME2 2019.4, https://docs.qiime2.org/2019.4/tutorials/) with truncation lengths of 280 bp (forward) and 220 bp (reverse), maxEE of 2, and Q20 threshold. Singleton amplicon sequence variants (ASVs) were removed. After denoising, merging, and chimera removal, non-singleton ASVs were obtained. A total of 2,276,858 raw reads were generated from 29 samples, averaging 78,512 ± 7,959 reads per sample. After quality filtering and chimera removal, 43,257 ± 6,573 high-quality reads per sample were retained. The sequencing data analyses were performed using QIIME2 and R packages (v3.2.0).

ASV-level alpha diversity indices, such as Chao1 richness, Shannon diversity index, and Simpson index were calculated using the ASV table in QIIME2. Beta diversity analysis was performed to investigate the structural variation of microbial communities across samples using Jaccard metrics (Jaccard, 1908) and visualized using principal coordinate analysis (PCoA) and non-metric multidimensional scaling (NMDS). A Venn diagram was generated to visualize the shared and unique ASVs among samples or groups using the R package “Venn Diagram” based on the occurrence of ASVs across samples/groups irrespective of their relative abundance. The linear discriminant analysis (LDA) effect size (LEfSe) was performed to detect differentially abundant taxa across groups using the default parameters. This analysis was conducted from the phylum level to the genus level based on the following criteria: LDA threshold > 2; P < 0.05.

Phylogenetic Investigation of Communities by Reconstruction of Unobserved States 2 (PICRUSt2) is a widely used bioinformatics tool for predicting functional potential of gut microbiota based on 16S rRNA gene sequences (Wright and Langille, 2025). In this study, Microbial functions were predicted using Arginine and proline metabolism (phylogenetic investigation of communities by reconstruction of unobserved states, https://github.com/picrust/picrust2) in MetaCyc (https://metacyc.org/) and Kyoto Encyclopedia of Genes and Genomes (KEGG) (https://www.kegg.jp/) databases.

### 2.11 Statistical analysis

GraphPad Prism 8.0 (GraphPad Software Inc. San Diego, CA, USA) was used for data analysis and plotting. Data are presented as mean ± standard deviation. Differences among groups were analyzed using one-way analysis of variance (ANOVA). Dunnett’s *post hoc* test was used for multiple comparisons, with the model group serving as the control. Statistical significance was set at P < 0.05 (two-tailed). Spearman correlation analysis between immune features and gut microbiota was performed as an exploratory analysis, and no correction for multiple comparisons was applied.

## 3 Results

### 3.1 SMZJ alleviated IDPN-induced stereotypical behaviors

**Figure 1:**
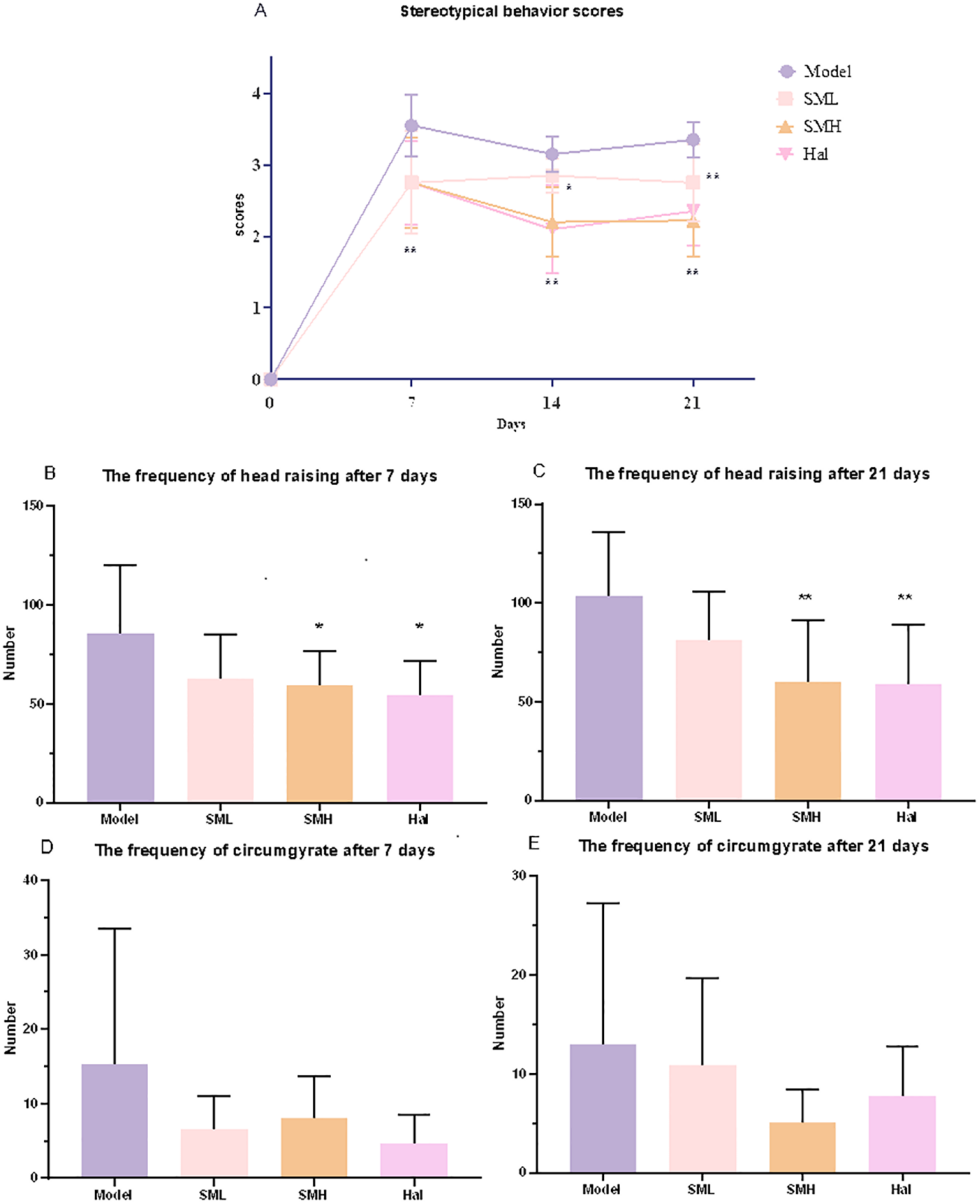
Shao-Ma-Zhi-Jing (SMZJ) granules alleviated 3,3′-iminodipropionitrile (IDPN) -induced stereotypical behaviors. **(A)** Stereotypic behavior scores. **(B)** The frequency of head raising behavior on day 7 post-drug administration. **(C)** The frequency of head raising behavior on day 21 post-drug administration. **(D)** The frequency of circumgyration behavior on day 7 post-drug administration. **(E)** The frequency of circumgyration behavior on day 21 post-drug administration. ^*^P < 0.05 and ^**^P < 0.01 vs. model group. Ctrl, control group; SML, low-dose SMZJ-treated group; SMH, high-dose SMZJ-treated group; Hal, haloperidol-treated group.

The therapeutic effects of SMZJ were evaluated using the IDPN-induced TS model. Stereotypical behavior was observed in the model group but not in the Ctrl group. On days 7, 14, and 21 post-drug administration, the scores of stereotyped behaviors in the SML, SMH, and Hal groups were significantly lower than those in the model group (P < 0.05, **Figure 1A**). To further quantify the effects of drugs on the behavior of the IDPN-induced TS rat model, representative stereotypes (head raising and circumgyration) were selected for recording. The number of stereotyped head raising and circumgyration behaviors was observed for 5 min in rats on days 7 and 21 post-drug administration. The stereotyped behaviors of rats in the model group persisted after day 21 post-drug administration. The frequency of 5-min stereotyped head raising behaviors in the SMH and Hal groups on days 7 and 21 post-drug administration was lower than that in the model group (P < 0.05, **Figure 1B**), especially on day 21 post-drug administration (P < 0.01, **Figure 1C**). The frequency of circumgyration behavior in the SML, SMH, and Hal groups has a tendency to decrease on days 7 and 21 post-drug administration (**Figures 1D, E**). These behavioral results indicated that SMZJ, especially at a high dose, alleviated the stereotyped behavior of the IDPN-induced TS rat model.

### 3.2 SMZJ exerted protective effects on the thymus and spleen in the IDPN-induced TS rat model

**Figure 2:**
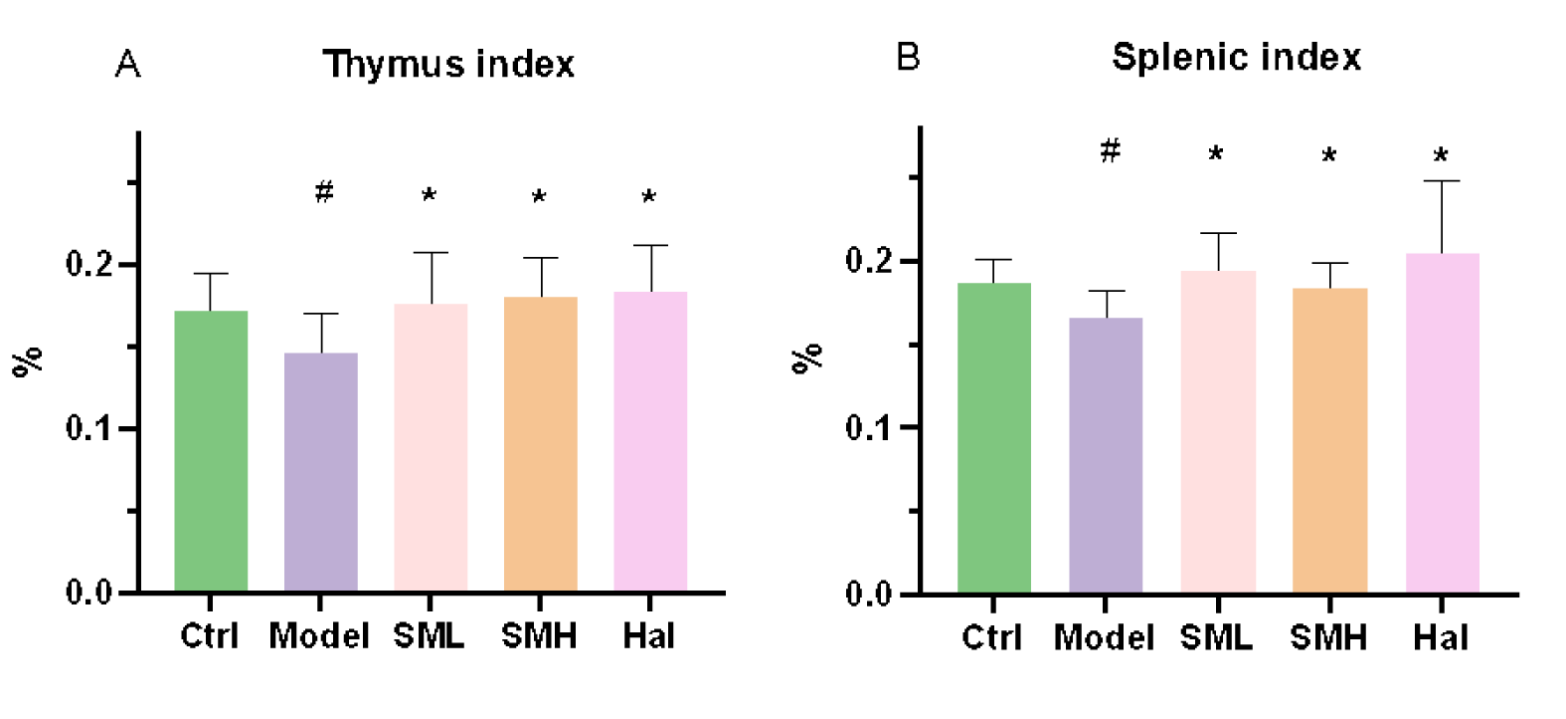
Shao-Ma-Zhi-Jing (SMZJ) granules upregulated the thymus and spleenic indices. **(A)** Thymus index. **(B)** Splenic index. ^#^P < 0.05 vs. Ctrl group, ^*^P < 0.05 vs. model group. Ctrl, control group; SML, low-dose SMZJ-treated group; SMH, high-dose SMZJ-treated group; Hal, haloperidol-treated group.

The thymus is critical for immune system function and is a major lymphoid organ involved in the development of T cells (Thapa and Farber, 2019). The spleen, which initiates both innate and adaptive immune responses, contains immune cells (T cells and B cells) and distributes immune cells to non-lymphoid tissues (Bronte and Pittet, 2023). The thymic and splenic indices provide a crude assessment of immune function. Compared with those in the Ctrl group, the thymic and splenic indices were significantly lower in the model group (P < 0.05, **Figure 2**). Meanwhile, the thymic and splenic indices in the SML, SMH, and Hal groups were significantly higher than those in the model group (P < 0.05, **Figure 2**). Treatment with SMZJ suppressed the IDPN-induced downregulation of thymic and splenic indices, suggesting that SMZJ can protect the immune organs thymus and spleen.

### 3.3 SMZJ regulated the levels of CD3^+^, CD4^+^, and CD8^+^ T cells

**Figure 3:**
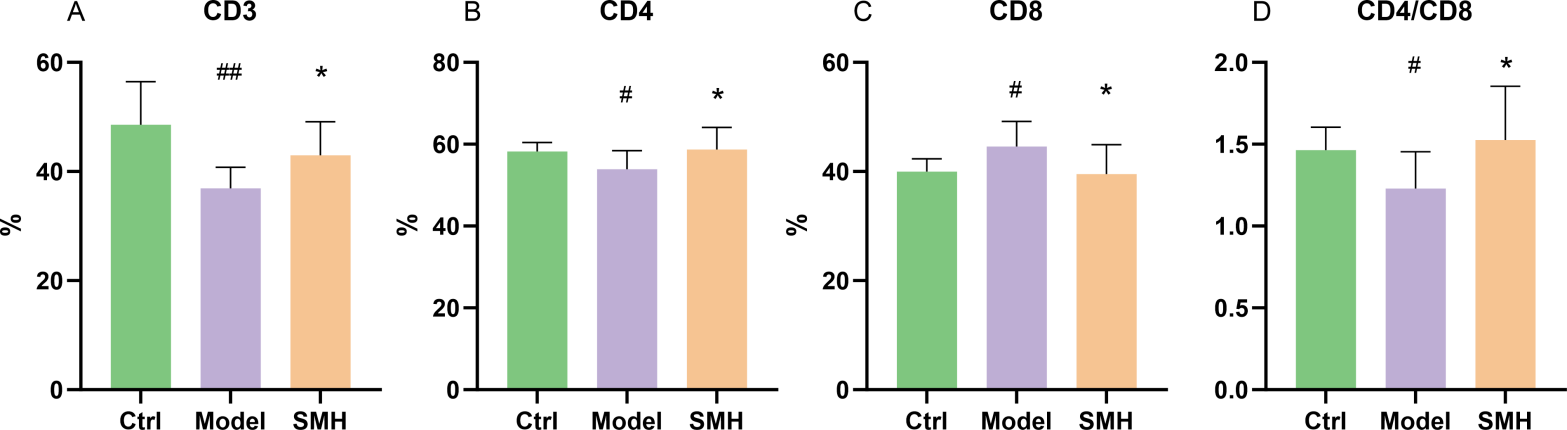
Shao-Ma-Zhi-Jing (SMZJ) granules regulated the proportions of CD3^+^, CD4^+^, and CD8^+^ T cells. **(A)** CD3^+^ cell proportion. **(B)** CD4^+^ cell proportion. **(C)** CD8^+^ cell proportion. **(D)** The CD4^+^ cell proportion to CD8^+^ cell proportion ratio. ^#^P < 0.05, ^##^P < 0.01 vs. Ctrl group; ^*^P<0.05 vs. model group. Ctrl, control group; Model, model group; SMH, high-dose SMZJ-treated group.

Flow cytometric analysis revealed that compared with those in the Ctrl group, the CD3^+^ (P < 0.01) and CD4^+^ T cell proportions (P < 0.05) and the CD4^+^/CD8^+^ ratio (P < 0.05) (**Figure 3**) were significantly lower and the CD8^+^ T cell proportions were significantly higher (P < 0.05) in the model group. Compared with those in the model group, the CD3^+^ T cell and CD4^+^ T cell proportions and the CD4^+^/CD8^+^ ratio were significantly higher (P < 0.05) and the CD8^+^ T cell proportions were significantly lower (P < 0.05) in the SMH group (**Figure 3**). These results indicated that SMZJ promotes the differentiation of T cells in the IDPN-induced TS rat model.

### 3.4 SMZJ regulated the transcription factors in splenic T helper 1 (Th1), Th2, T regulatory (Treg), and Th17 cells and improved the Th1/Th2 balance

**Figure 4:**
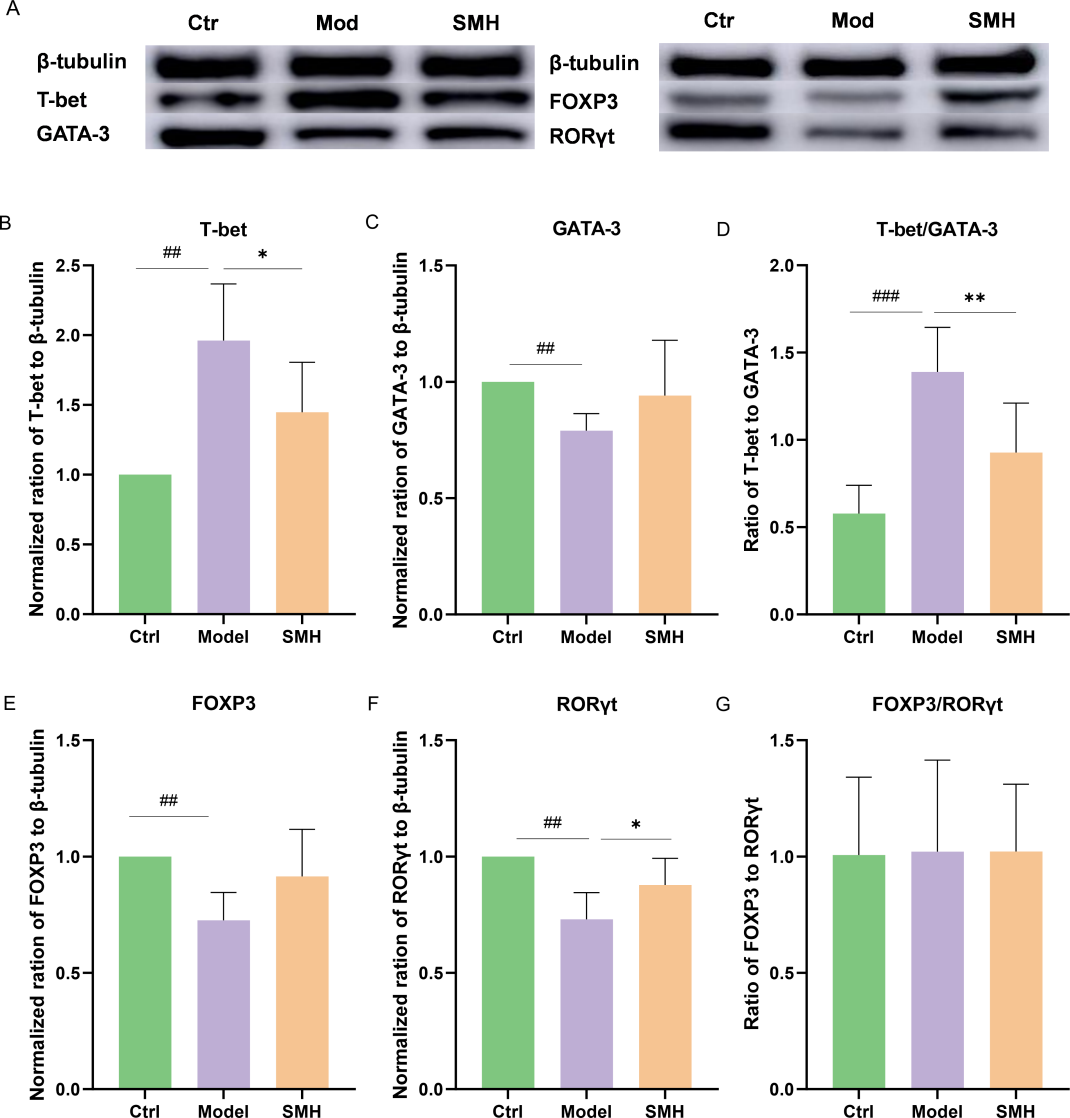
The protein levels of T-bet, GATA-3, FOXP3, and RORγt in the spleen. **(A)** Western blotting analysis of T-bet, GATA-3, FOXP3, and RORγt protein levels. **(B)** The protein expression levels of T-bet. **(C)** The protein expression levels of GATA-3. **(D)** The T-bet expression to GATA-3 expression ratio. **(E)** The protein expression levels of FOXP3. **(F)** The protein expression levels of RORγt. **(G)** The FOXP3 expression to RORγt expression ratio. ^##^P < 0.01, ^###^P < 0.001 vs. Ctrl group; ^*^P < 0.05, ^**^P < 0.01 vs. model group. Ctrl, control group; SML, high-dose Shao-Ma-Zhi-Jing (SMZJ)-treated group.

During CD4^+^ T cell differentiation, different transcription factors and cytokines determine the generation of Th cell types, including Th1, Th2, Th17, and Treg cells (Sun et al., 2023). T-bet and GATA-3 promote Th1 and Th2 cell differentiation, respectively. Additionally, T-bet antagonizes Th2 and Th17 cell differentiation by inhibiting the functions of GATA-3 and RORγt, respectively. RORγt, and FOXP3 are major regulatory transcription factors for Th17 and Treg cell development, respectively (Li et al., 2014). To examine the effect of SMZJ on the differentiation of CD4^+^ T cells in the TS rat model, the protein levels of the splenic Th1 cell transcription factor T-bet, the Th2 cell transcription factor GATA-3, the Treg cell transcription factor FOXP3, and the Th17 cell transcription factor RORγt were examined (**Figure 4A**). Compared with those in the Ctrl group, the T-bet levels (P < 0.01) and the T-bet expression to GATA-3 expression ratio were significantly higher (P < 0.001; **Figures 4B, D**) and the GATA-3, FOXP3, and RORγt levels were significantly lower (all P < 0.01; **Figures 4C, E, F**) in the model group. Meanwhile, compared with those in the model group, the T-bet levels (P < 0.05) and the T-bet expression to GATA-3 expression ratio were significantly lower (P < 0.01; **Figures 4B, D**), the RORγt levels were significantly higher (P < 0.05; **Figure 4F**). The GATA-3 and FOXP3 levels, and the ratio of FOXP3 to RORγt expression, were not significantly higher in the SMH group (all P > 0.05; **Figures 4C, E, G**).

**Figure 5:**
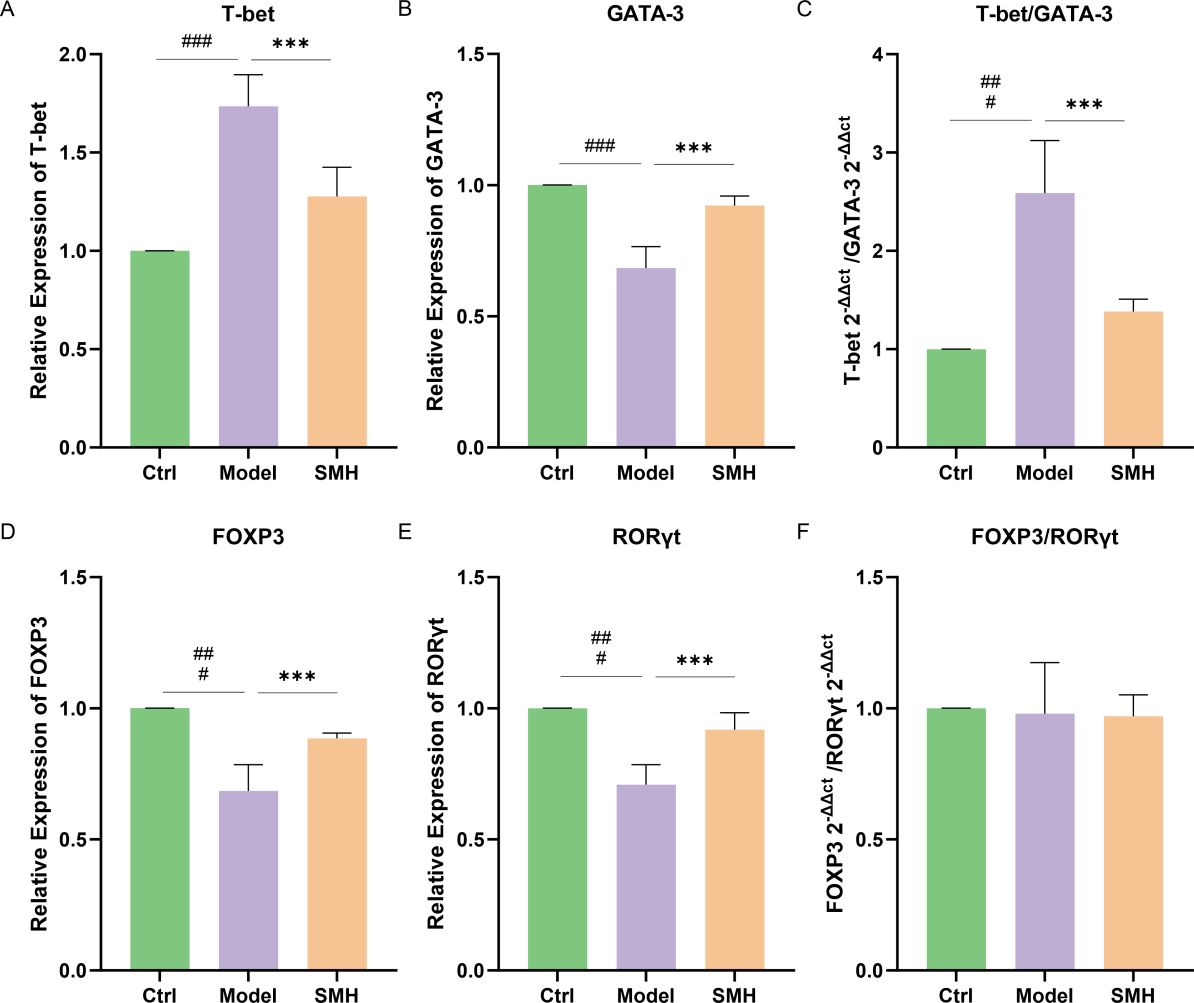
The relative mRNA expression levels of *T-bet*, *Gata-3*, *Foxp3*, and *Rorγt* in the spleen. **(A)** The mRNA expression levels of *T-bet*. **(B)** The mRNA expression levels of *Gata-3*. **(C)** The *T-bet* expression to *Gata-beita3* expression ratio. **(D)** The mRNA expression levels of *Foxp3*. **(E)** The mRNA expression levels of *Rorγt*. **(F)** The *Foxp3* expression to *Rorγt* expression ratio. ^###^P < 0.001 vs. Ctrl group; ^***^P < 0.001 vs. model group. Ctrl, control group; SML, high-dose Shao-Ma-Zhi-Jing (SMZJ)-treated group.

The mRNA levels of *T-bet*, *Gata-3*, *Foxp3*, and *Rorγt* were analyzed using qRT-PCR analysis (**Figure 5**). Compared with those in the Ctrl group, the *T-bet* mRNA levels and the *T-bet* expression to *Gata-3* expression ratio were significantly upregulated (P < 0.001), the *Gata-3*, *Foxp3*, and *Rorγt* mRNA levels were significantly downregulated (P < 0.001), and the *Foxp3* expression to *Rorγt* expression ratio was not significantly different (P > 0.05) in the model group. Meanwhile, compared with those in the model group, the *T-bet* mRNA levels and the *T-bet* expression to *Gata-3* expression ratio were significantly lower (P < 0.001), the *Gata-3*, *Foxp3*, and *Rorγt* mRNA levels were significantly higher (P < 0.001), and the *Foxp3* expression to *Rorγt* expression ratio was not significantly different (P > 0.05) in the SMH group. Thus, SMZJ regulated the mRNA levels of the Th1 and Th2 cell transcription factors *Foxp3* and *Rorγt* in the TS rat model (P < 0.001) but not the *Foxp3* expression to *Rorγt* expression ratio (P > 0.05). Additionally, SMZJ regulated the mRNA levels of the Th1, Th2, Treg, and Th17 cell transcription factors in the TS rat model and improved the Th1/Th2 balance.

**Figure 6:**
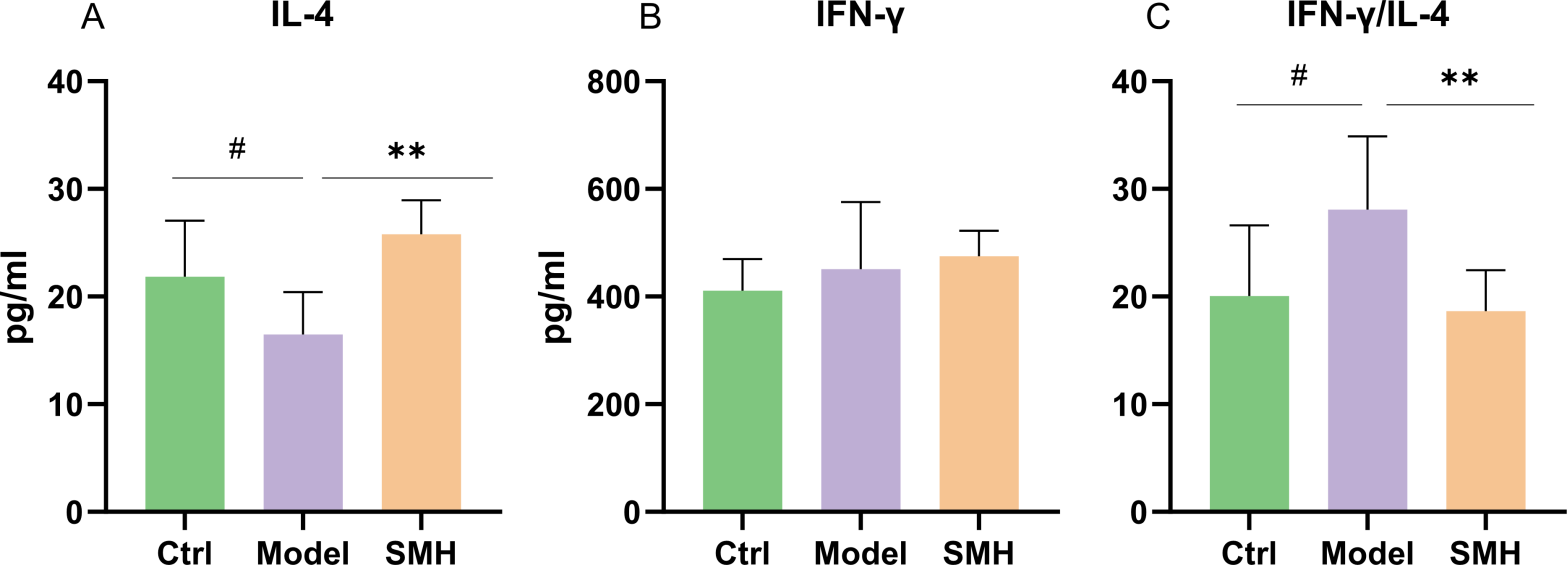
Shao-Ma-Zhi-Jing (SMZJ) granules improved the balance of T helper 1 (Th1)/Th2 cell cytokines. The serum levels of IL-4 **(A)** and IFN-γ **(B)**. **(C)** The IL-4 to IFN-γ ratio. ^#^P < 0.05 vs. Ctrl group; ^**^P < 0.01 vs. model group. Ctrl, control group; SMH, high-dose SMZJ-treated group.

Western blotting and qRT-PCR analyses suggested that SMZJ regulates the Th1/Th2 balance. IFN-γ is a characteristic cytokine of Th1 cells, while Th2 cells produce IL-4 (Zhu et al., 2010). Compared with those in the Ctrl group, the IL-4 levels were significantly downregulated (P < 0.05, **Figure 6A**) and the IFN-γ level to IL-4 level ratio was significantly upregulated (P < 0.05, **Figure 6C**) in the model group. Meanwhile, compared with those in the model group, the IL-4 levels were significantly upregulated (P < 0.05, **Figure 6A**) and the IFN-γ level to IL-4 level ratio was significantly downregulated (P < 0.01, **Figure 6C**) in the SMH group. These findings suggest that SMZJ regulates the level of IL-4 and improves the balance of Th1/Th2 cytokines.

Thus, SMZJ modulates the levels of Th1, Th2, Treg, and Th17 cell transcription factors in the rat spleen and improves the Th1/Th2 balance.

### 3.5 SMZJ downregulated the cAMP/PI3K/NF-κB inflammatory signaling pathway in the brain striatum

**Figure 7:**
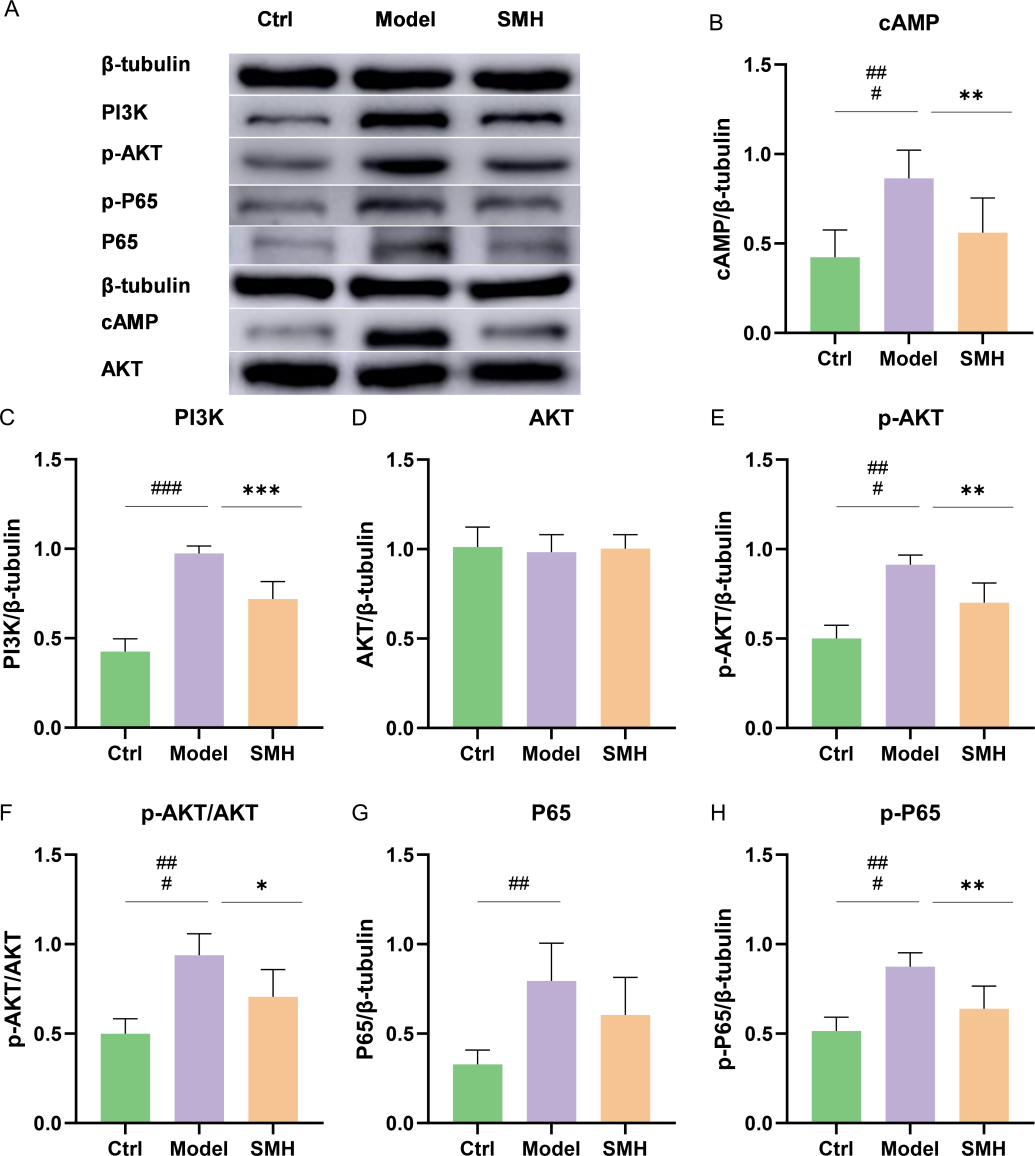
Shao-Ma-Zhi-Jing (SMZJ) granules downregulated the cAMP/PI3K/NF-κB inflammatory signaling pathway in the brain striatum. **(A)** Western blotting analysis of cyclic adenosine monophosphate (cAMP), PI3K, AKT, p-AKT, p-P65, and P65. The protein expression levels of cAMP **(B)**, PI3K **(C)**, AKT **(D)**, p-AKT **(E)**, p-P65 **(G)**, and P65 **(H)**. **(F)** The p-AKT expression to AKT expression ratio. ^##^P < 0.01, ^###^P < 0.001 vs. Ctrl group; ^*^P < 0.05, ^**^P < 0.01, ^***^P < 0.001 vs. model group. Ctrl, control group; SMH, high-dose SMZJ-treated group.

cAMP regulates PI3K, which is one of the key effectors of PI3K of protein kinase B (AKT). PI3K/AKT can regulate downstream genes, such as those encoding the nuclear transcription factor NF-κB (*RELA*). NF-κB is a redox-regulated transcription factor and is mainly involved in the regulation of inflammatory/immune responses, apoptosis, and cell growth (Jiang et al., 2022). To examine the regulatory effects of SMZJ on the inflammatory pathway in the TS rat model, the levels of cAMP/PI3K/NF-κB related signaling pathway-related proteins were examined in the striatum of the brain (**Figure 7A**). The levels of cAMP (P < 0.001), PI3K (P < 0.001), p-AKT (P < 0.001), p-P65 (P < 0.001), and P65 (P < 0.01) and the p-AKT/AKT ratio (P < 0.001) in the model group were significantly higher than those in the Ctrl group (**Figure 7**). Meanwhile, the levels of cAMP (P < 0.01), PI3K (P < 0.001), p-AKT (P < 0.01), and p-P65 (P < 0.01) and the p-AKT/AKT ratio (P < 0.05) in the SMH group were significantly downregulated when compared with those in the model group (**Figure 7**). Western blotting analysis demonstrated that the activity of the cAMP/PI3K/NF-κB signaling pathway was upregulated in the TS rat brain striatum but downregulated in the SMH group.

### 3.6 SMZJ downregulated inflammatory cytokines in the TS rat model

**Figure 8:**
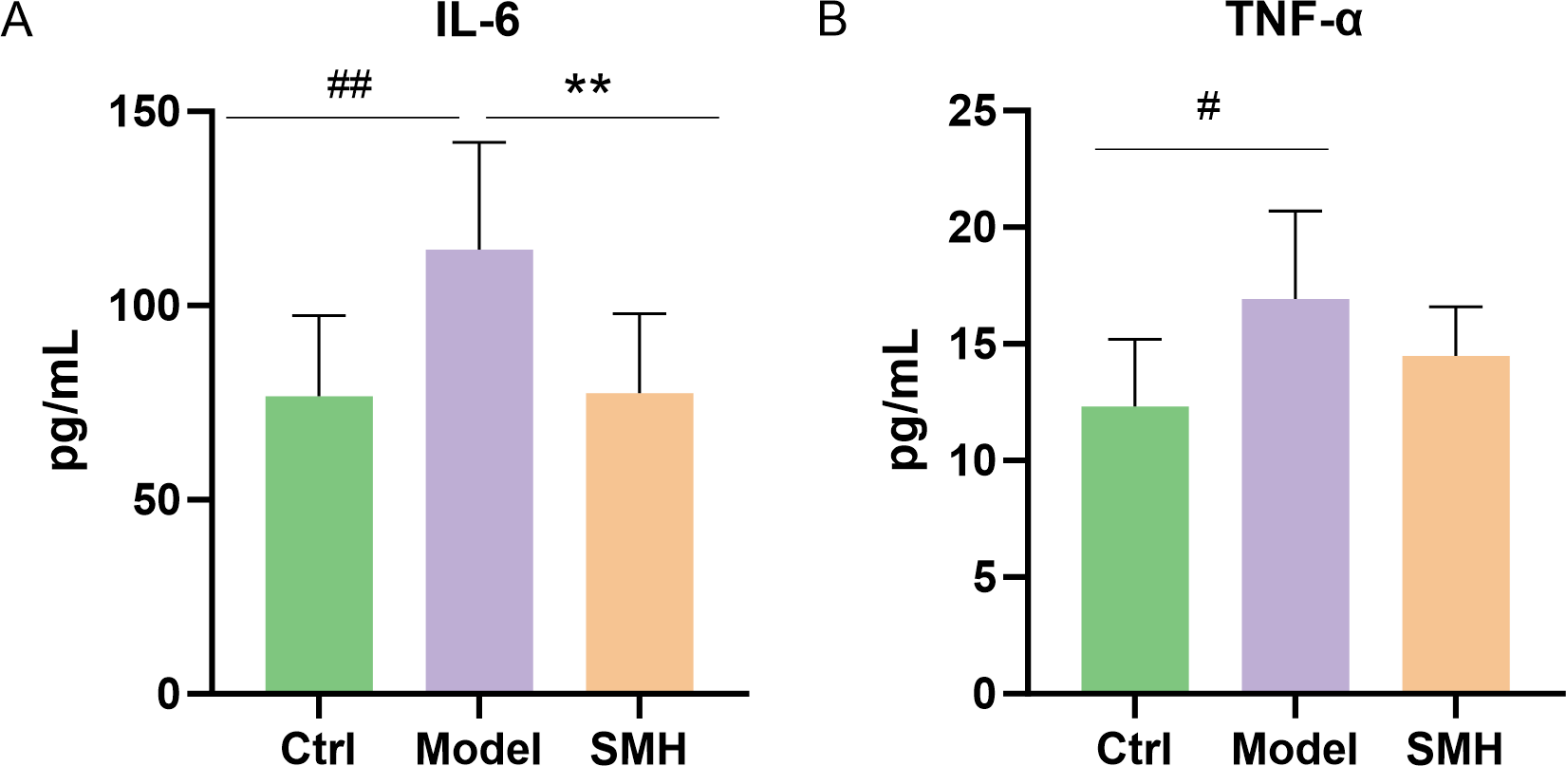
Shao-Ma-Zhi-Jing (SMZJ) granules downregulated the levels of the inflammatory factors IL-6 and TNF-α. The serum levels of IL-6 **(A)** and TNF-α **(B)**. ^#^P < 0.05, ^##^P < 0.01 vs. Ctrl group; ^**^P < 0.01 vs. model group. Ctrl, control group; SMH, high-dose SMZJ-treated group.

NF-κB initiates the transcription of pro-inflammatory molecules, including cytokines (IL-6 and TNF-α) (Sivandzade et al., 2019). Pediatric patients with TS exhibit elevated levels of pro-inflammatory cytokines, including TNF-α and IL-6 (Li et al., 2022). IL-6, a cytokine involved in the inflammatory cascade response, is produced by various hematopoietic and non-hematopoietic cells and exerts a pro-inflammatory effect. In contrast, TNF-α, which is secreted by macrophages, monocytes, neutrophils, CD4^+^ T cells, and other cells, exerts pro-inflammatory effects and is involved in physiological inflammatory and immune responses (Hohensinner et al., 2011). To investigate the effect of SMZJ on inflammation in the TS rat model, the serum levels of IL-6 and TNF-α were examined (**Figure 8**). The ELISA results demonstrated that the IL-6 (P < 0.01) and TNF-α (P < 0.05) levels in the model group were significantly higher than those in the Ctrl group (**Figure 8**). Meanwhile, the IL-6 (P < 0.01) levels in the SMH group were significantly lower than those in the model group (P < 0.01, **Figure 8A**). The TNF-α levels in the SMH group were non-significantly lower than those in the model group (P > 0.05, **Figure 8B**). Thus, SMZJ modulated the levels of the inflammatory cytokines TNF-α and IL-6 in the TS rat model.

### 3.7 SMZJ promotes microglial activation in the rat brain striatum

**Figure 9:**
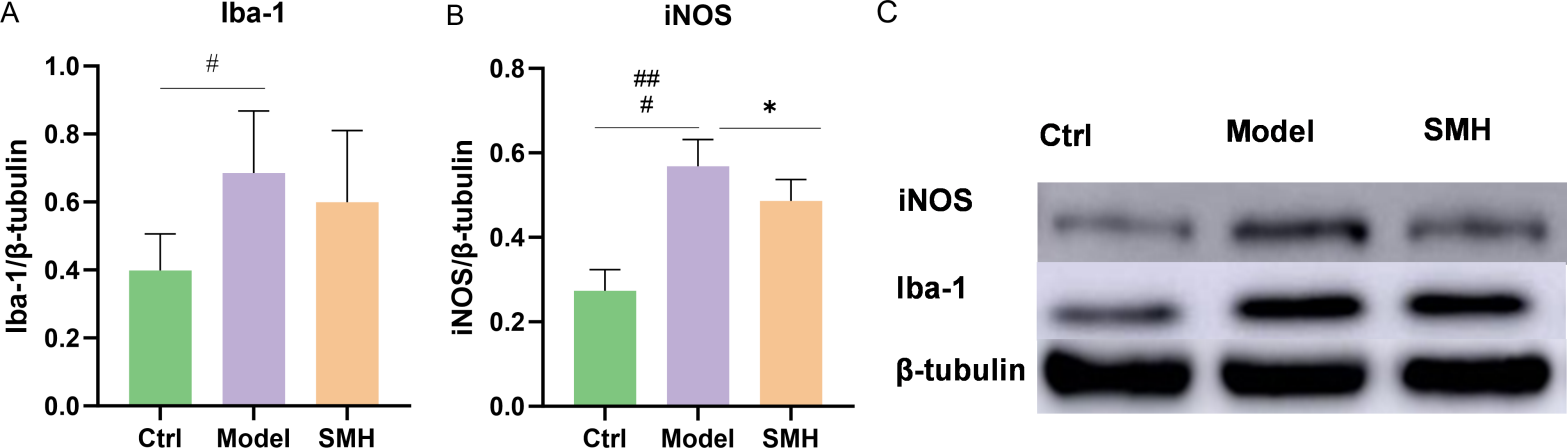
Shao-Ma-Zhi-Jing (SMZJ) granules inhibited microglial activation in the rat brain striatum. The protein expression levels of Iba-1 **(A)** and iNOS **(B)**. ^#^P < 0.05, ^###^P < 0.01 vs. Ctrl group; ^*^P < 0.05 vs. model group. Ctrl, control group; SMH, high-dose SMZJ-treated group.

iNOS and Iba-1 are markers of M1 microglia and activated microglia, respectively (Tang et al., 2023). To examine the effects of SMZJ on microglia in the brain striatum of the TS rat model, the brain striatum was subjected to western blotting analysis to examine the levels of iNOS and Iba-1 (**Figure 9C**). Compared with those in the Ctrl group, the Iba-1 (P < 0.05) and iNOS levels were significantly higher (P < 0.001) (**Figure 9**) in the model group. Meanwhile, compared with those in the model group, the iNOS levels were significantly lower (P < 0.05; **Figure 9B**) and the Iba-1 levels were non-significantly lower (P > 0.05) in the SMH group. Thus, SMZJ inhibited the activation of M1-type microglia in the striatum and regulated microglial activation.

### 3.8 SMZJ increased the diversity of gut microbiota and regulated community composition

**Figure 10:**
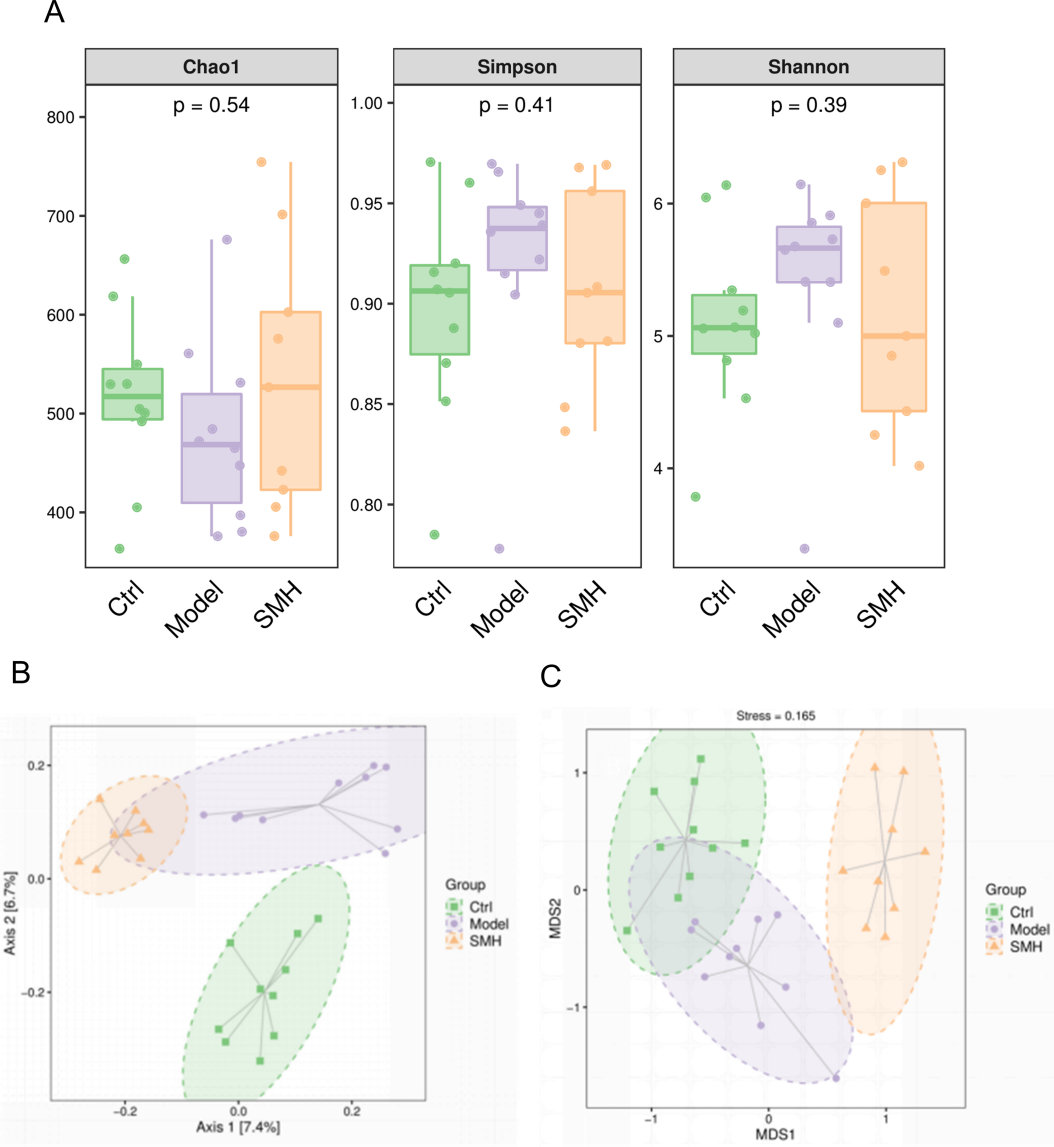
Shao-Ma-Zhi-Jing (SMZJ) granules increased the diversity of gut microbiota. **(A)** Chao1, Simpson, and Shannon indices. **(B)** Principal coordinate analysis results. **(C)** Non-metric multidimensional scaling analysis results. Ctrl, control group; SMH, high-dose SMZJ-treated group.

Gut microbiota complexity was positively correlated with Shannon and Chao1 indices and negatively correlated with Simpson index. In α-diversity, no differences in gut microbial richness and evenness were identified among the groups (Chao1 *p* =  0.54, Simpson *p* = 0.41, and Shannon *p* = 0.39) (**Figure 10A**). The β diversity index of the intestinal flora was calculated by downscaling the multidimensional microbiological data using PCoA and NMDS. The results of PCoA (**Figure 10B**) and NMDS (**Figure 10C**) revealed the distinct clustering of the gut microbiota from the Ctrl, model, and SMH groups. Both PCoA and NMDS suggested that the gut microbiota composition varied between the Ctrl, model, and SMH groups.

**Figure 11:**
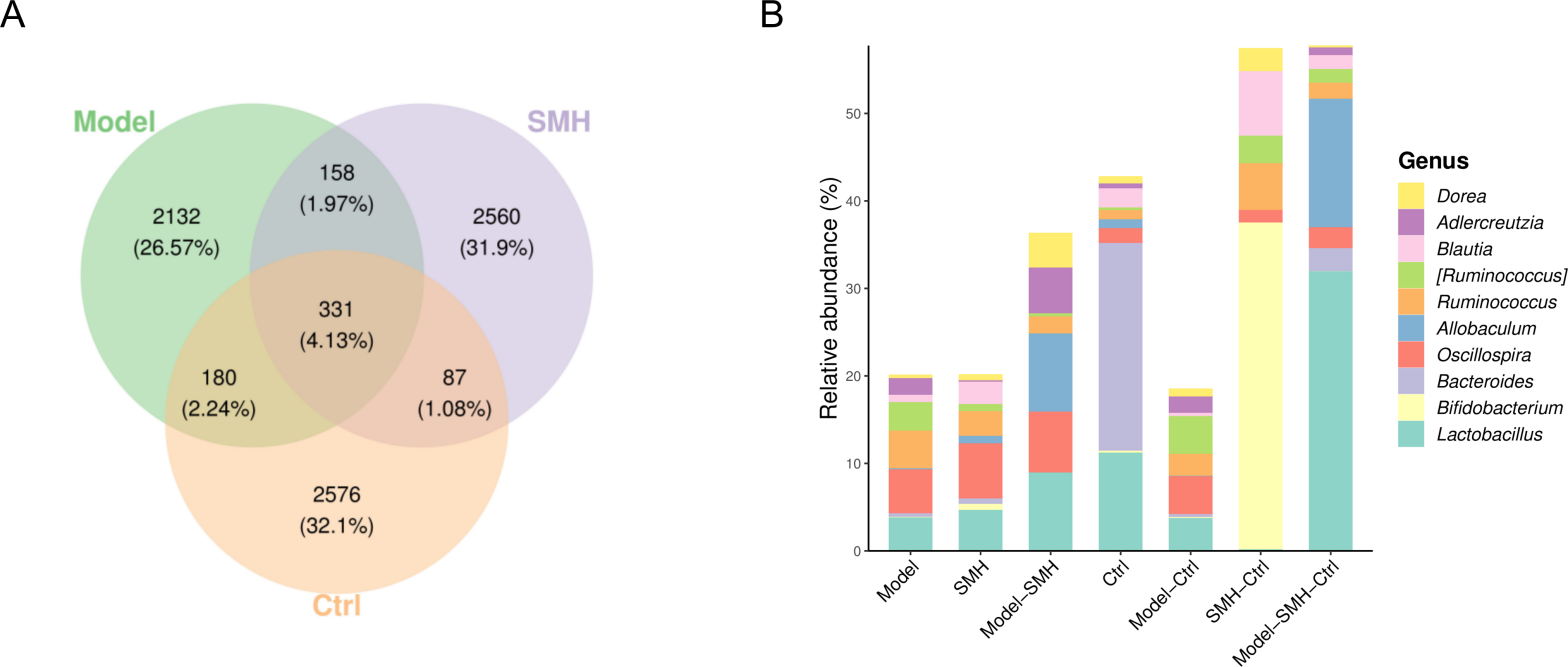
Species abundance analysis. **(A)** Venn diagram. **(B)** Genus-level relative abundance of amplicon sequence variants (ASVs).

Next, the microbial composition was analyzed at the genus level (**Figure 11**). Compared with those in the Ctrl group, the abundances of *Lactobacillus*, *Blautia*, *Dorea*, *Allobaculum*, *Bifidobacterium*, and *Bacteroides* were lower and the abundances of *Oscillospira*, *Ruminococcus*, *Adlercreutzia*, and *[Ruminococcus]* were higher in the model group. Meanwhile, compared with those in the model group, the abundances of *Lactobacillus*, *Dorea*, *Blautia*, *Allobaculum*, *Bifidobacterium*, and *Bacteroides* were higher and the abundances of *Ruminococcus*, *Adlercreutzia*, and *[Ruminococcus]* were lower in the SMH group.

**Figure 12:**
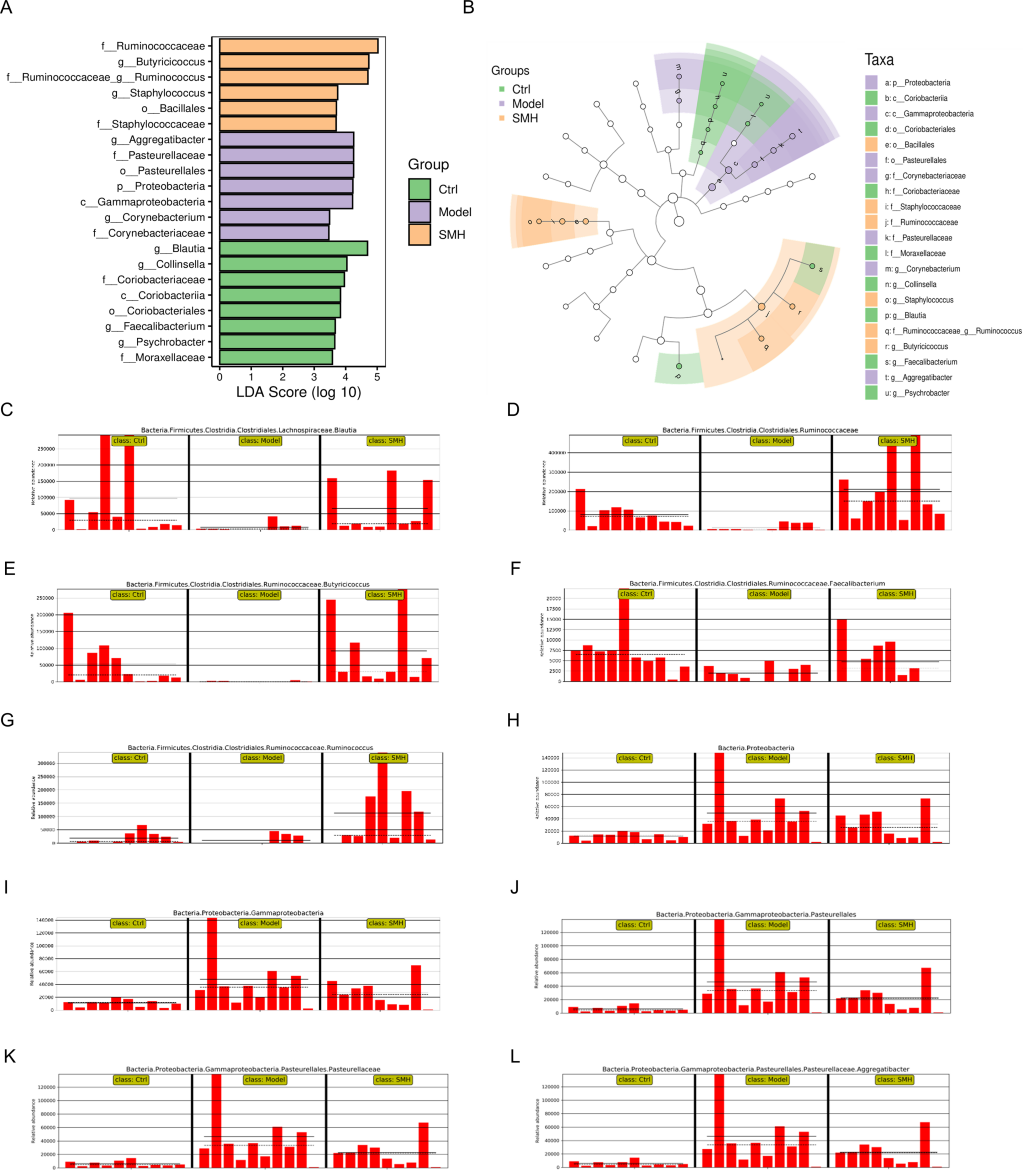
Results of linear discriminant analysis (LDA) effect size (LEfSe) analysis. **(A)** Marker species for each group. **(B)** LEfSe-generated cladogram showing differential gut microbial abundances between the Ctrl, model, and SMH groups. **(C–L)** Relative abundance of marker species. The solid line indicates the mean relative abundance of the marker species in each subgroup, while the dashed line represents the median relative abundance, reflecting the differences between groups through the mean and median values. Ctrl, control group; SMH, high-dose SMZJ-treated group.

A histogram of the distribution of LDA values for significantly different species was used to show the species that were significantly enriched within each group and their level of importance. In total, 21 marker species were screened in the three groups (**Figure 12A**). The biological classification of the three groups of marker species was mainly distributed in six phyla/classes/orders, such as p_Proteobacteria, c_Coriobacteriia, and c_Gammaproteobacteria (**Figure 12B**). To further analyze the specific distribution of marker species between groups, the intergroup abundance of individual marker species was analyzed. The abundances of g_Blautia, f_Ruminococcaceae, g_Butyricicoccus, g_Faecalibacterium, and f_Ruminococcaceae_g_Ruminococcus were downregulated, whereas those of p_Proteobacteria, c_Gammaproteobacteria, o_Pasteurellales, f_Pasteurellaceae, and g_Aggregatibacter were upregulated in the model group. The abundances of these marker species in the SMH group were opposite to those in the model group (**Figures 12C–L**).

### 3.9 SMZJ improved microbial metabolism and function based on PICRUSt2 prediction

The PICRUSt2 tool was used to assess the potential functional alterations in the gut microbial composition in distinct groups of rats. The differential KEGG and MetaCyc metabolic pathways between the groups were predicted based on P ≤ 0.05. Specific results are listed in **Tables 3**–**5**. Differential KEGG metabolic pathway analysis revealed that compared with the Ctrl group, the model group exhibited changes in three metabolic pathways (including the downregulation of glycosphingolipid biosynthesis-lacto and neolacto series pathways and the upregulation of ethylbenzene degradation and polycyclic aromatic hydrocarbon degradation pathways). Compared with the model group, the SMH group exhibited downregulation of three metabolic pathways and upregulation of three metabolic pathways (including the upregulation of the glycosphingolipid biosynthesis-lacto and neolacto_series pathway). These findings suggest that SMZJ upregulated the glycosphingolipid biosynthesis-lacto and neolacto_series pathway in the TS rat model.

**Table 3:** Differential Kyoto Encyclopedia of Genes and Genomes (KEGG) metabolic pathway results between the Ctrl and model groups.

Pathway	logFC	P-value
Ethylbenzene degradation	2.446	0.05185
Polycyclic aromatic hydrocarbon degradation	1.445	0.03954
Glycosphingolipid biosynthesis-lacto and neolacto_series	−1.75	0.04951

Ctrl, control group; FC, fold-change.

**Table 4:** Differential Kyoto Encyclopedia of Genes and Genomes (KEGG) metabolic pathway results between the model and SMH groups.

Pathway	logFC	P-value
Vibrio cholerae infection	2.96	0.004103
Glycosphingolipid biosynthesis-lacto and neolacto_series	2.767	7.60E-05
Hypertrophic cardiomyopathy (HCM)	1.216	0.04666
Vasopressin-regulated water reabsorption	−1.451	0.02992
Steroid biosynthesis	−2.52	0.0306
Limonene and pinene degradation	−4.54	0.009398

SMH, high-dose Shao-Ma-Zhi-Jing-treated group; FC, fold-change.

**Table 5:** Differential MetaCyc metabolic pathway results.

Pathway	Ctrl vs. model		Model vs. SMH	
	logFC	P-value	logFC	P-value
Chorismate biosynthesis II (archaea)	−1.974	3.35E-06	1.237	0.06703
Superpathway of ornithine degradation	−0.9945	0.05592	0.9469	0.0314
Androstenedione degradation	0.09181	0.05885	−0.09841	0.03426

Ctrl, control group; FC, fold-change; SMH, high-dose Shao-Ma-Zhi-Jing-treated group.

Differential MetaCyc metabolic pathway analysis revealed that compared with those in the Ctrl group, chorismate biosynthesis II (archaea) and superpathway of ornithine degradation pathways were downregulated, and the androstenedione degradation pathway was upregulated in the model group. Meanwhile, compared with those in the model group, the chorismate biosynthesis II (archaea) and superpathway of ornithine degradation pathways were significantly upregulated and the androstenedione degradation pathway was significantly downregulated in the SMH group. SMH mitigated the IDPN-induced changes in chorismate biosynthesis II (archaea), superpathway of ornithine degradation, and androstenedione degradation pathways in rats.

### 3.10 Analysis of the correlation between immune features and gut microbiota

**Figure 13:**
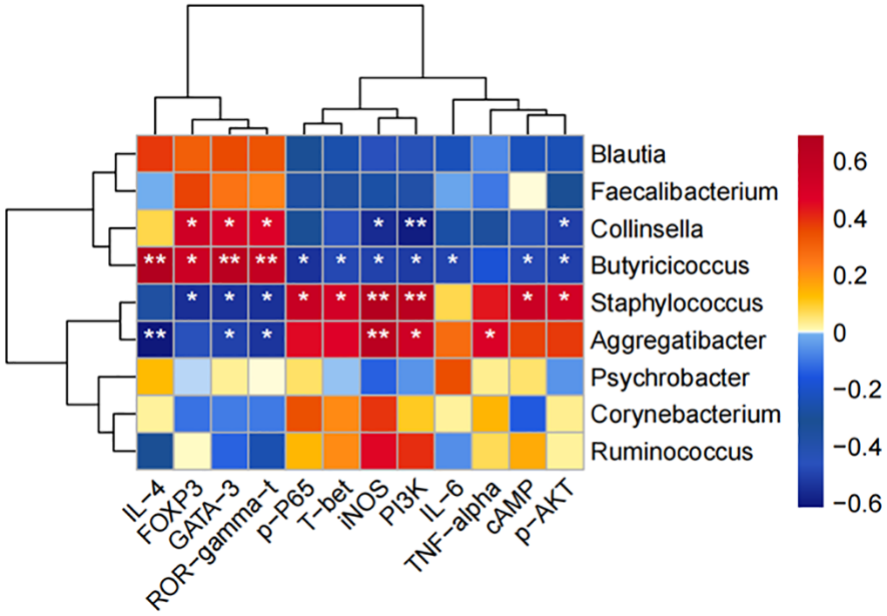
Spearman correlation analysis. Red boxes indicate the microbial species whose abundances are positively correlated with clinical indicators. Blue boxes indicate the microbial species whose abundances are negatively correlated with clinical indicators. ^*^P < 0.05 and ^**^P < 0.01. Note: Spearman correlation analyses are exploratory. No correction for multiple comparisons was applied.

To examine the correlation between gut microbiota abundance at the genus level and immunological parameters, a Spearman correlation analysis was performed (**Figure 13**). The inflammatory pathway-associated proteins, inflammatory cytokines, Th1 cell transcription factors, and striatal indicators were negatively correlated with the abundances of *Blautia*, *Faecalibacterium*, *Collinsella*, and *Butyricoccus* and positively correlated with the abundances of *Staphylococcus* and *Aggregatibacter*. Additionally, Th2 cell transcription factors, Th2 cytokine, Th17 transcription factors, and Treg transcription factors were positively correlated with the abundances of *Blautia*, *Faecalibacterium*, *Collinsella*, and *Butyricoccus* and negatively correlated with the abundances of *Staphylococcus* and *Aggregatibacter*. The abundances of *Collinsella*, *Butyricoccus*, *Staphylococcus*, and *Aggregatibacter* were significantly correlated with immune indicators. These data suggested the correlation between immune parameters and gut microbiota in the TS rat model.

## 4 Discussion

IDPN, a neurotoxin, disrupts the extrapyramidal DA system and persistently downregulates the DA concentration, leading to DA receptor hypersensitivity and stereotyped behavior, which is similar to dopamine dysfunction in TS. Therefore, IDPN can be used as a modeling agent in TS pharmacodynamic studies. The IDPN-induced rat model was adopted in this study due to its extensive application in preclinical exploratory investigations of tic-related behavioral changes (Liu et al., 2021). Epidemiological data indicate that the male-to-female ratio in patients with TS is approximately 3:1 (74.5% male vs. 25.5% female) (Dy-Hollins et al., 2024). Accordingly, male Sprague Dawley rats were selected for modeling. SMZJ administration significantly improved IDPN-induced stereotypical behaviors in rats (**Figure 1**).

TS is characterized by immune dysfunctions, which include alterations in T cells, effector molecules, and neuronal immunity (Hsu et al., 2021). Immune mechanisms may be involved in the pathophysiology of TS. Children with TS exhibit a disrupted T-cell subset equilibrium. The proportions of CD3^+^ T cells and CD4^+^ T cells are downregulated in children with TS, whereas the proportion of CD8^+^ T cells is upregulated, and the CD4^+^/CD8^+^ ratio is decreased (Ruan et al., 2007; Li et al., 2022). The proportions of CD3^+^ T cells and CD4^+^ T in the TS rat model in this study were consistent with those in patients with TS. CD4^+^ T cells can differentiate into Th1, Th2, Th17, and Treg cells. This study examined the protein and mRNA levels of transcription factors of these four cells and the cytokines of Th1 and Th2 cells. SMZJ modulated the splenic levels of Th1, Th2, Treg, and Th17 transcription factors and improved the Th1/Th2 cytokine balance. These results suggest that SMZJ regulates T cell differentiation in the TS rat model. Th1 cells secrete inflammatory cytokines, such as IFNG, which promote the function of macrophages and their production of NO and reactive oxygen intermediates. Th2 cells are involved in humoral and allergic immune responses and produce anti-inflammatory cytokines, such as IL-4, which suppress macrophage activation, mitigating inflammatory responses (Bao et al., 2022). Treg cells, which exhibit immunosuppressive and anti-inflammatory activities, can suppress immune responses to CNS antigens and inhibit neuroinflammation to maintain immune homeostasis (Xie et al., 2015). This suggests that SMZJ ameliorates peripheral inflammation in the TS rat model.

PI3K inhibitors modulating the PI3K/Akt/NF-κB pathway alleviated symptoms and improved cytokine levels (IL-6, TNF-α, and IL-1β) in the 2,5-dimethoxy-4-iodoamphetamine-induced twitching rat model (Hongyan et al., 2017). The serum levels of inflammatory factors were upregulated in the TS rat model, which was consistent with the serum inflammatory cytokine profiles of clinical patients with TS (Li et al., 2022). SMZJ downregulated the brain striatal cAMP/PI3K/Akt/NF-κB signaling pathway and the levels of inflammatory cytokines. The TLR4-mediated differentiation pathway activates NF-κB phosphorylation, promoting CD4^+^ T cell activation and differentiation and exerting immunomodulatory activity in the innate and adaptive immune system (Wang et al., 2020). Antipsychotics can also regulate T cell subsets (Th1/Th2 cells) through the AKT/NF-κB-related pathway (Chen et al., 2011). In the TS rat model, the cAMP/PI3K/Akt/NF-κB signaling pathway and the levels of Th1 cell transcription factors were significantly upregulated and the levels of Th2, Th17, and Treg transcription factors were downregulated. SMZJ might affect the immunoregulatory activity of T cells possibly through the cAMP/PI3K/Akt/NF-κB signaling pathway, which may be involved in regulating T cell differentiation. The activation/polarization of microglia, which are resident macrophages in the CNS, promotes neuroinflammation. Typically activated microglia (type M1) release pro-inflammatory mediators (such as TNF-α, IL-6), exacerbating inflammatory injury. iNOS and Iba-1 are the markers of M1 microglia and microglial activation, respectively. SMZJ downregulated the NOS2 levels in the striatum of the TS rat model, suggesting that SMZJ inhibits M1-type microglial activation and suppresses neuroinflammatory responses in the TS rat model. NF-κB activates iNOS under inflammatory conditions, promoting the production of nitric oxide (NO) and eliciting immune responses (Stachon et al., 2021). Thus, SMZJ might mitigate neuroinflammatory responses by regulating NF-κB/iNOS to alleviate neuroinflammation in the TS rat model. Proinflammatory cytokines including TNF-α and IL-6 are able to activate the NF-κB pathway, which suppresses the expression of ZO-1 and other tight junction proteins and consequently elevates intestinal permeability (Marchiando et al., 2010; Mohammad and Thiemermann, 2021). Tight junction proteins, including the cytoskeletal adaptor protein ZO-1 and transmembrane proteins occludin and claudin-1, form stable intercellular complexes to maintain intestinal barrier structural and functional integrity (Marchiando et al., 2010). In the present study, the increased level of TNF-α in TS rats may contribute to intestinal barrier impairment via disrupting tight junction stability. In contrast, SMZJ treatment significantly downregulated TNF-α levels in TS rats, suggesting that SMZJ could alleviate inflammation-related intestinal barrier damage by inhibiting TNF-α-mediated inflammatory cascades. These results suggest that SMZJ modulates peripheral immune indices including T cell differentiation and inflammatory cytokines, alleviates neuroinflammation via regulating NF-κB pathways and microglia in the brain striatum of the TS rats, and might exert protective effects on intestinal barrier function.

As reported by Chu et al., gut microbiota is pivotal in regulating gut-brain axis communication (Chu et al., 2025). In the present study, the gut microbiota composition of model rats was compared with that of normal control rats. No significant difference in α-diversity was observed between the two groups, whereas β-diversity differed markedly. Consistent with our results, a clinical study using 16S rRNA sequencing reached similar conclusions (Bao et al., 2023). It revealed no significant differences in gut microbial α-diversity between children with TS and healthy controls, whereas obvious disparities were observed in β-diversity. Such differences imply evident structural changes in gut microbiota, which is a typical sign of intestinal flora dysbiosis among TS patients. Gut microbiota composition is correlated with immunity. Dysbiosis may modulate inflammatory cytokine levels and cerebral microglial activity as well as inflammatory signaling, which in turn affects intestinal barrier permeability. In this study, SMZJ restored the IDPN-induced changes in the diversity and structure of the gut microbiota in the TS rat model. In particular, SMZJ restored the IDPN-induced changes in the abundances of the following 10 marker species: g_Blautia, f_Ruminococcaceae, g_Butyricoccus, g_Faecalibacterium, f_Ruminococcaceae_g_Ruminococcus, p_Proteobacteria, c_Gammaproteobacteria, o_Pasteurellales, f_Pasteurellaceae, and g_Aggregatibacter. Decreased abundance of *Blautia*, a major acetate-producing genus, may lead to the downregulation of acetate, which is a major short-chain fatty acid involved in regulating immune homeostasis (Ye et al., 2023). *Faecalibacterium* modulates the immune system by upregulating metabolites and microbial anti-inflammatory molecules (Martín et al., 2023). *Butyricicoccus*, a beneficial bacterium, inhibits inflammation and exerts enteroprotective effects, and maintains intestinal barrier function by downregulating the intestinal myeloperoxidase, CD-α, and IL-12 levels (Eeckhaut et al., 2013). Gram-negative bacteria, such as *Pasteuriaceae* may induce neuroinflammation. Bacterial belonging to the f_Pasteuriaceae_g_Aggregatibacter group are pathogenic. A genus of coagulant bacilli (*Aggregatibacter actinomycetemcomitans*) can cause inflammation and induce immune responses in brain cells. The blood levels of *A. actinomycetemcomitans* are positively correlated with cytokine concentrations (Lamphere et al., 2023). Most marker species restored by SMZJ are short-chain fatty acids (SCFAs)-producing bacteria. SCFAs exert anti-inflammatory, immunomodulatory and neuroprotective effects, and penetrate the blood-brain barrier minimally (Banfi et al., 2021). Additionally, SCFAs regulate the expression of tight junction proteins, inhibit histone deacetylase, and modulate genes associated with immune signaling that govern epithelial cell turnover, proliferation and biological functions, thereby preserving intestinal epithelial integrity (Gilbert et al., 2018).

PICRUSt2 function prediction revealed that SMZJ upregulated glycosphingolipid biosynthesis-lacto and neolacto_series, chorismate biosynthesis II (archaea), superpathway of ornithine degradation, and androstenedione degradation pathways in the TS rat model. Lactosylceramide functions as a key signaling regulator by activating the Ras/ERK1/2 and NF-κB pathways to regulate *iNOS* expression (Pannu et al., 2004). In the microbiota, putrescine is synthesized from ornithine through ornithine decarboxylase-mediated catalysis and is further degraded to gamma-aminobutyric acid (GABA) (ornithine degradation pathway) (Rashmi et al., 2018). The levels of GABA, which is the major inhibitory neurotransmitter, are dysregulated in the GABAergic neurons in TS. Animal model experiments suggest that the striatal injection of GABA antagonists can induce motor tics. Clinical evidence suggests that GABAergic drugs aid in controlling tics (Deidda et al., 2014). Dysbiosis may lead to metabolic disorders and affect the development of TS. SMZJ might improve the symptoms of the TS rat model by regulating the metabolism of gut microbiota.

Spearman correlation analysis revealed that the abundances of *Staphylococcus* and *Aggregatibacter* were positively correlated with the levels of inflammatory indicators and negatively correlated with the levels of anti-inflammatory indicators. In contrast, the abundances of *Collinsella* and *Butyricoccus* were negatively correlated with the levels of inflammatory indicators and positively correlated with the levels of anti-inflammatory indicators. SMZJ decreased *Aggregatibacter* abundance and increased *Butyricoccus* abundance, suggesting that SMZJ upregulates the abundance of bacteria with anti-inflammatory properties and downregulates the abundance of bacteria with pro-inflammatory properties. SMZJ-induced modulation of the gut microbiota may be associated with alleviation of peripheral inflammation and neuroinflammation. Nevertheless, only correlations between specific gut microbiota and immune parameters were identified, and relevant causal validation approaches such as fecal microbiota transplantation, antibiotic depletion and gnotobiotic model establishment remain unperformed. Future studies employing these approaches are warranted to establish causality and elucidate the underlying mechanisms. In addition, although the bidirectional crosstalk between gut microbiota and the central nervous system has been well documented, the region-specific regulatory effects of gut microbiota on diverse brain subregions remain largely elusive. Chu et al. successfully constructed a LC-APCI-MS/MS analytical platform to realize accurate quantification of cerebral neurosteroids in male mice (Chu et al., 2021). Hence, this validated methodological strategy could be adopted to optimize and unify the quantitative scoring criteria for stereotyped behaviors in TS rats in subsequent research.

## 5 Conclusions

This study demonstrated that SMZJ significantly alleviated the symptoms of the TS rat model. SMZJ suppressed peripheral inflammation and neuroinflammation in the TS rat model. SMZJ alleviated peripheral and neuroinflammation in TS rats via promoting T cell differentiation and downregulating TNF-α and IL-6 levels, which may be related to changes in the cAMP/PI3K/Akt/NF-κB signaling pathway. SMZJ downregulated the activation of striatal M1 microglia in the TS model. SMZJ may potentially modulated gut microbiota structure and metabolism, relieves peripheral and neuroinflammation, and ameliorates intestinal mucosal barrier injury.

## Data Availability

The data presented in the study are deposited in the Mendeley Data repository, accession number https://doi.org/10.17632/246p9b4tp4.1.
