# Supplementary figures and images for: Shao-Ma-Zhi-Jing granules alleviate Tourette Syndrome by modulating the cAMP/PI3K/AKT/NF-κB signaling pathway, T cell differentiation, microglia, and gut microbiota
Source: Front Physiol. 2026 Jun 17;17:1827824. doi: 10.3389/fphys.2026.1827824 (PMC13318577; doi:10.3389/fphys.2026.1827824)

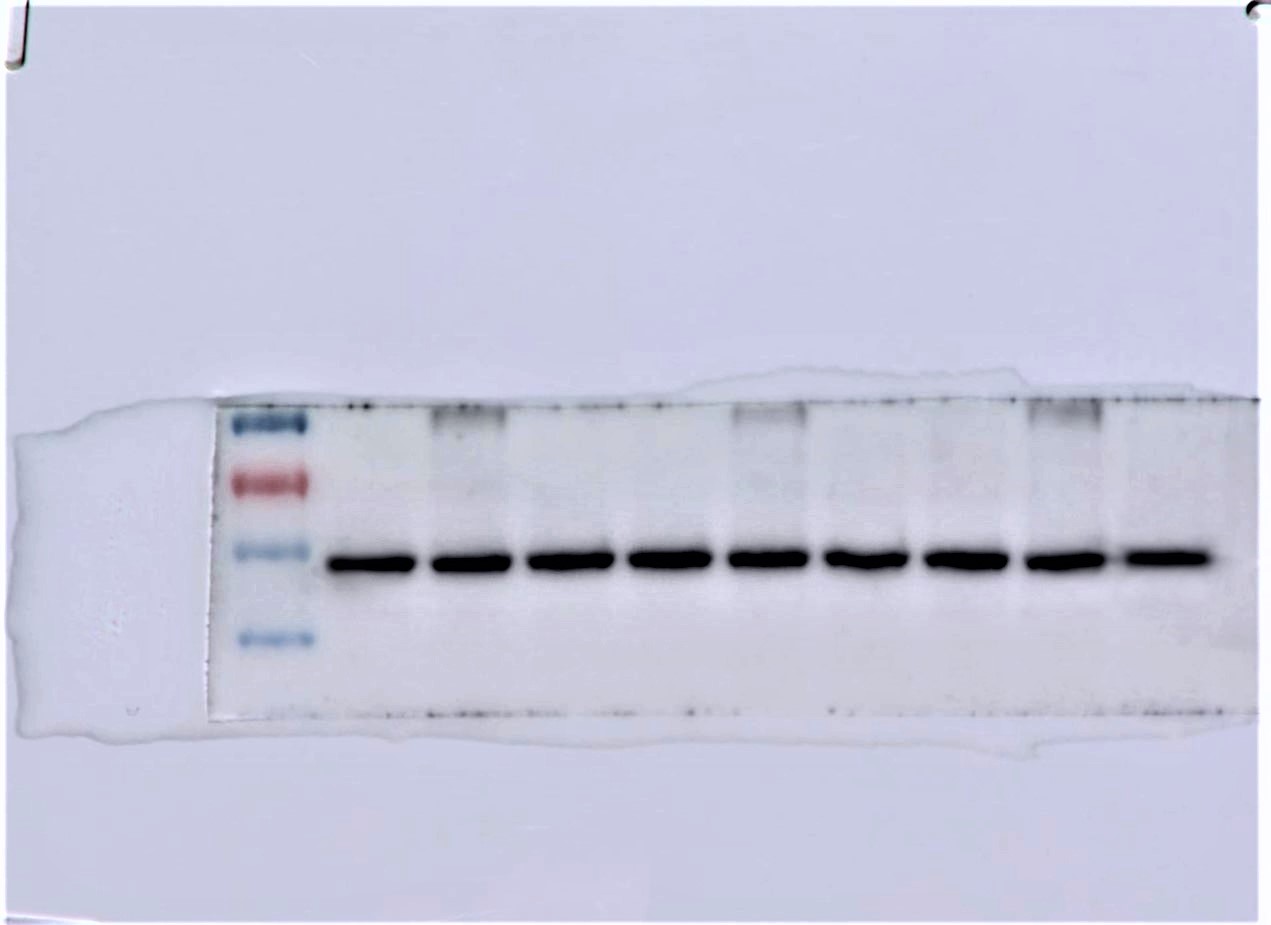

Supplement: Supplementary file 1 [file DataSheet1.zip › Western blotting-SMZJ/Western blotting-brain striatum-figure/AKT(56-62KD)-1.jpg]

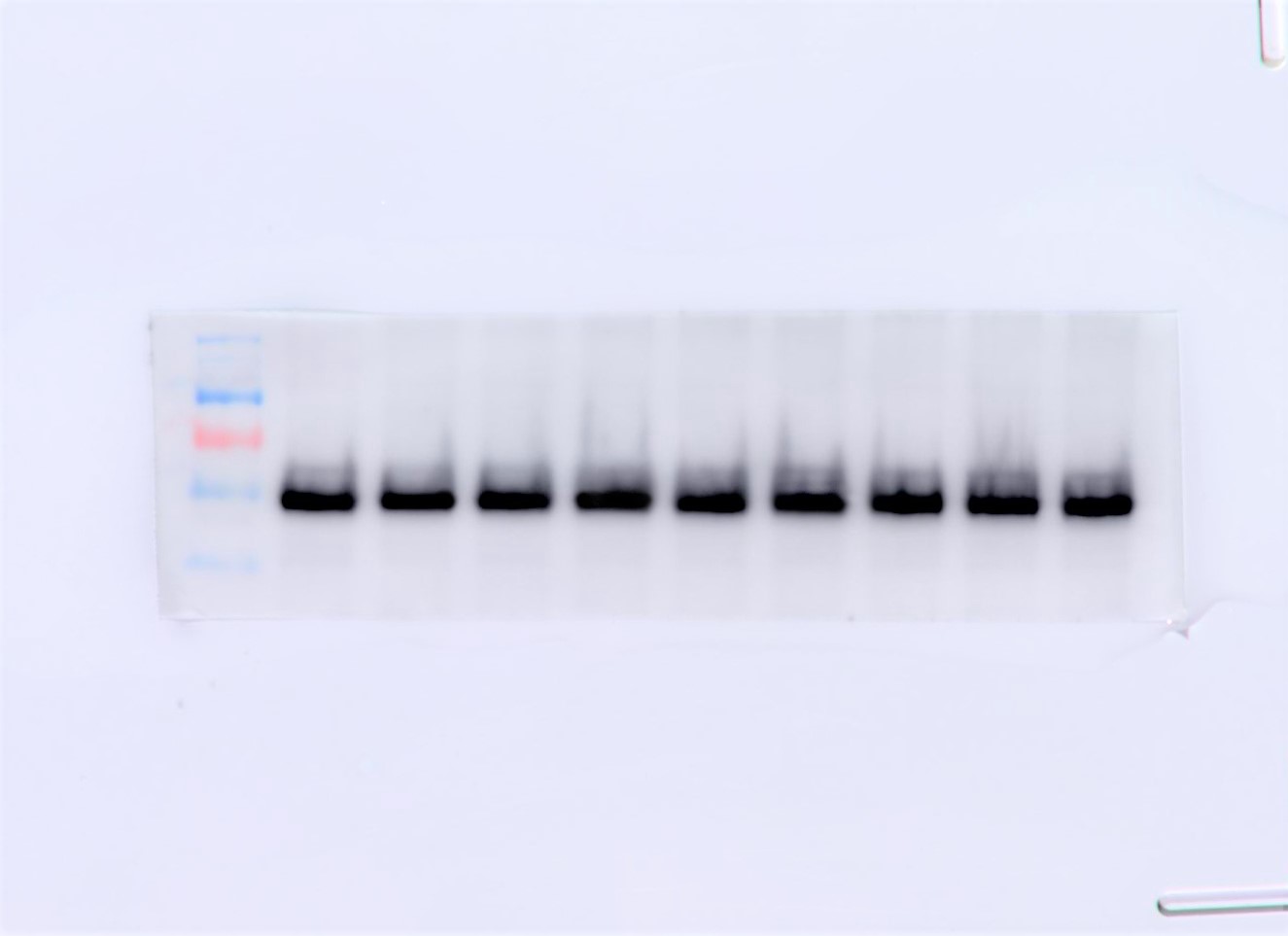

Supplement: Supplementary file 1 [file DataSheet1.zip › Western blotting-SMZJ/Western blotting-brain striatum-figure/AKT(56-62KD)-2.jpg]

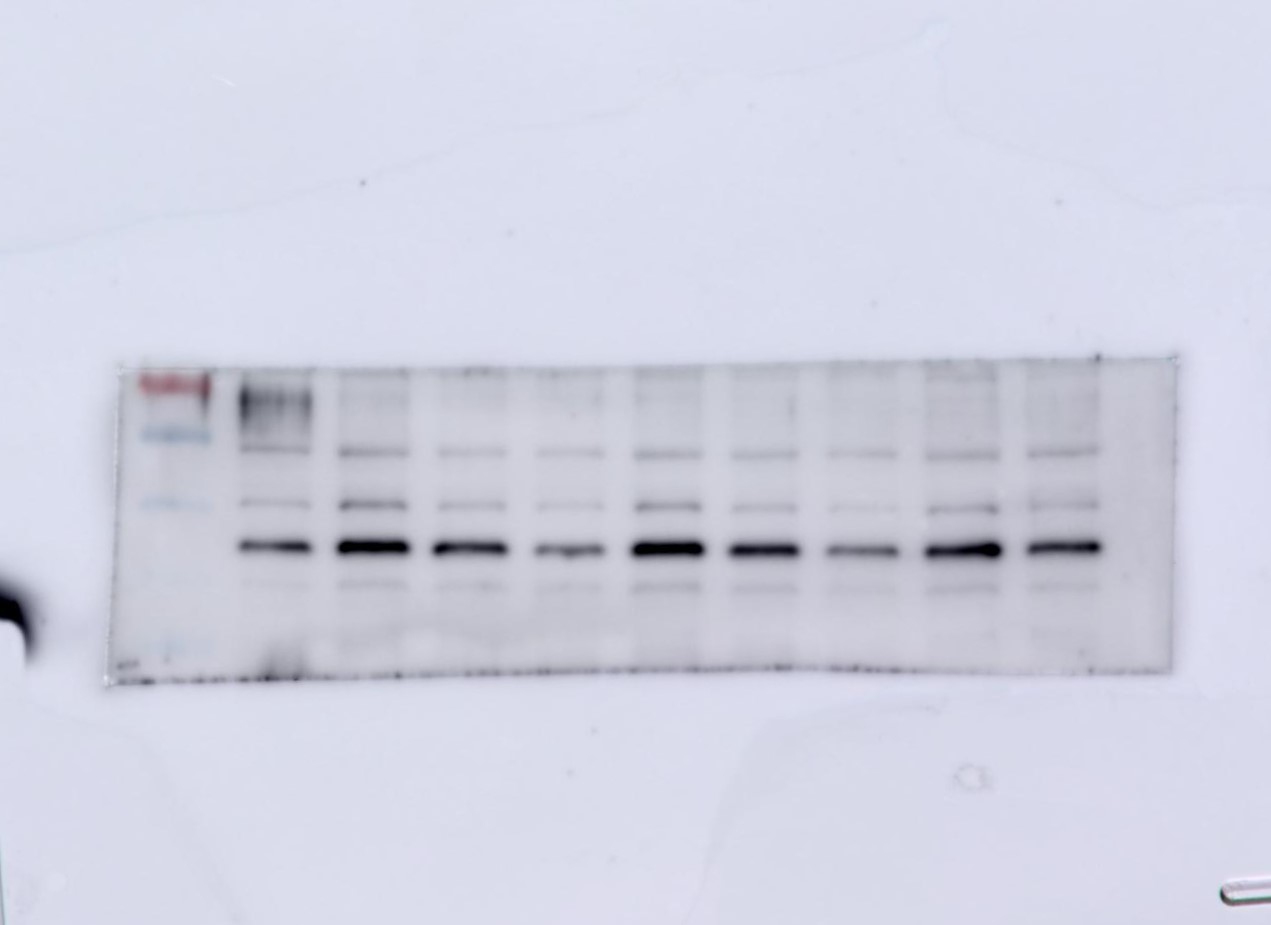

Supplement: Supplementary file 1 [file DataSheet1.zip › Western blotting-SMZJ/Western blotting-brain striatum-figure/ARG-1(36KD)-1.jpg]

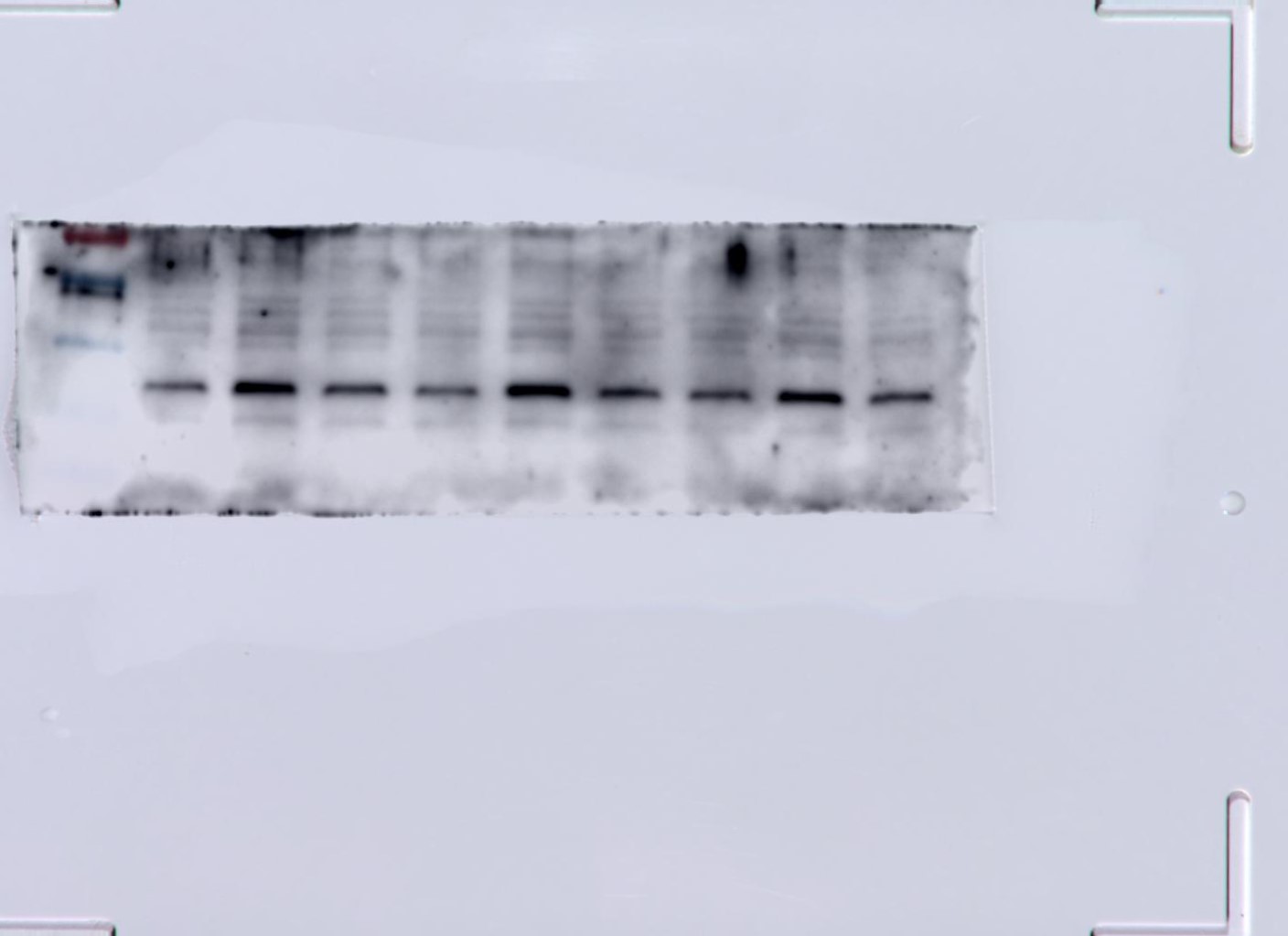

Supplement: Supplementary file 1 [file DataSheet1.zip › Western blotting-SMZJ/Western blotting-brain striatum-figure/ARG-1(36KD)-2.jpg]

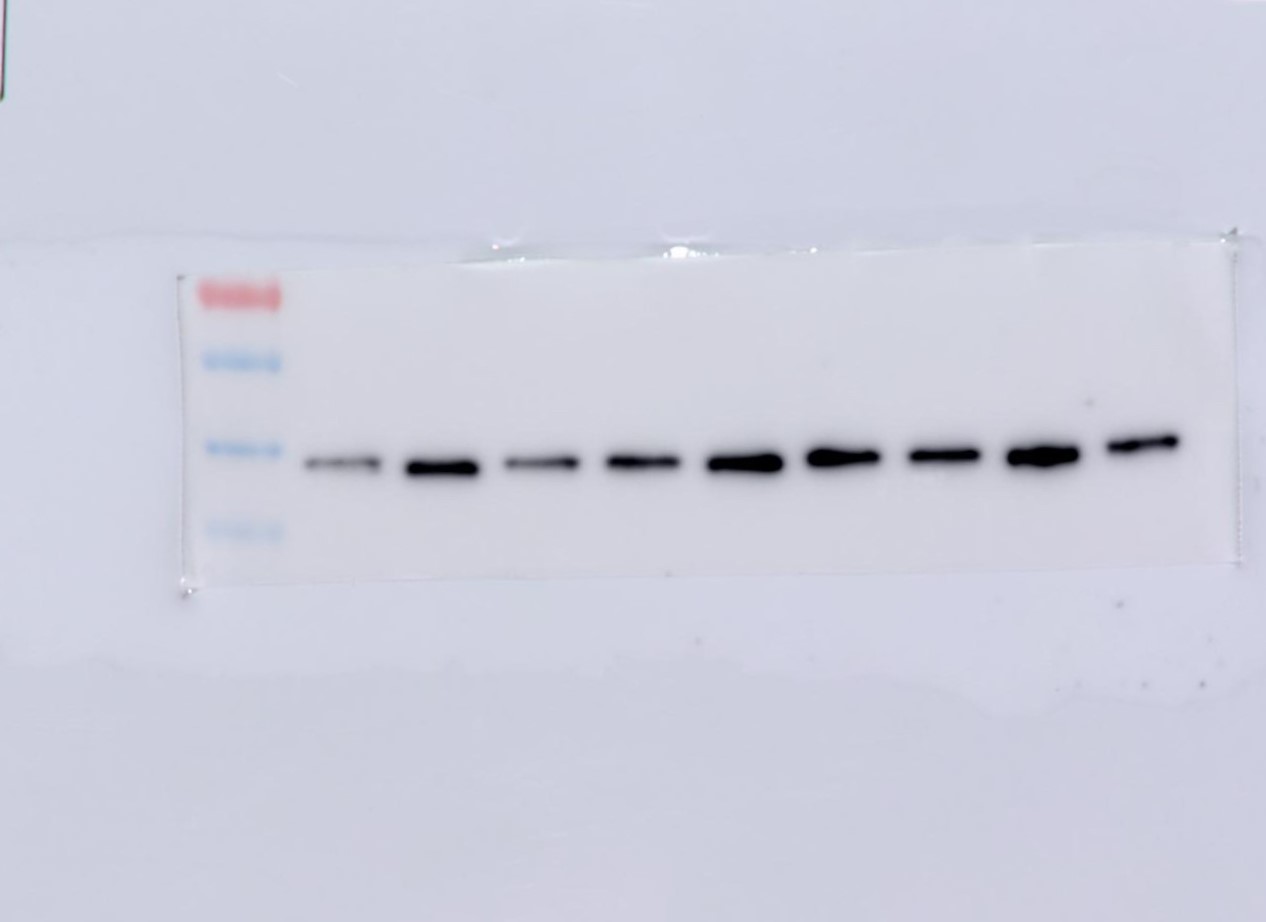

Supplement: Supplementary file 1 [file DataSheet1.zip › Western blotting-SMZJ/Western blotting-brain striatum-figure/CAMP(42,46KD)-1.jpg]

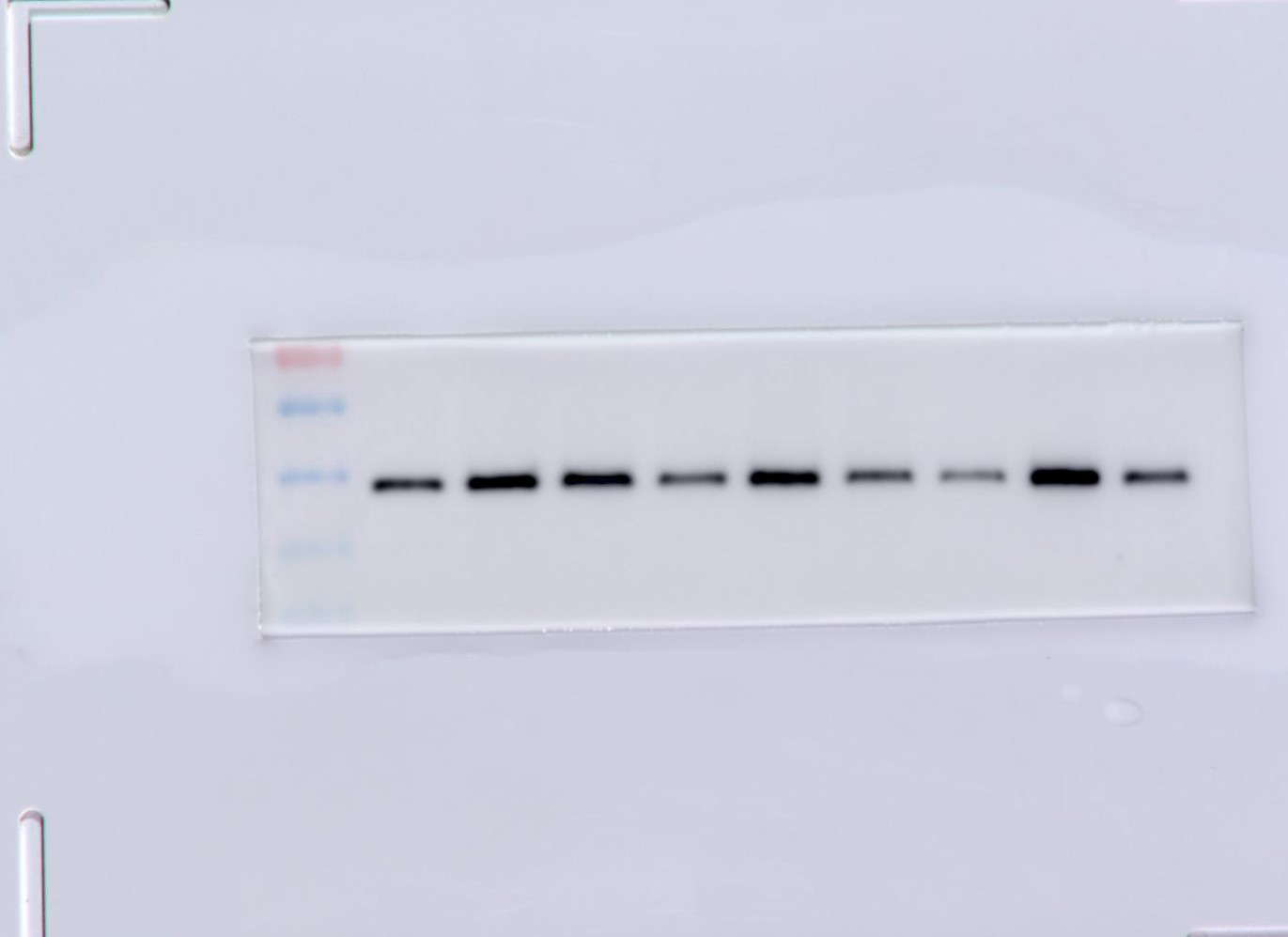

Supplement: Supplementary file 1 [file DataSheet1.zip › Western blotting-SMZJ/Western blotting-brain striatum-figure/CAMP(42,46KD)-2.jpg]

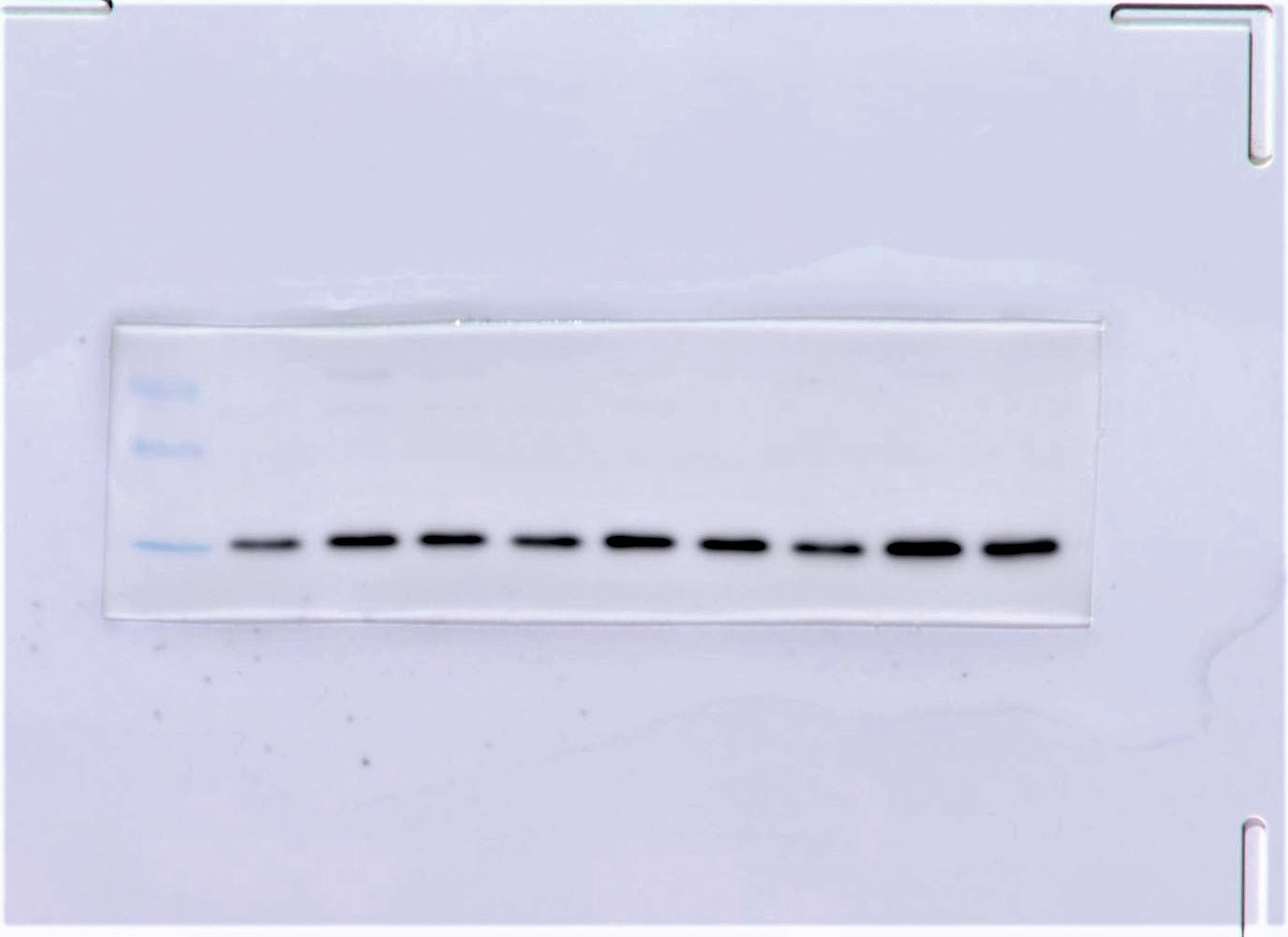

Supplement: Supplementary file 1 [file DataSheet1.zip › Western blotting-SMZJ/Western blotting-brain striatum-figure/iba1(16KD)-1.jpg]

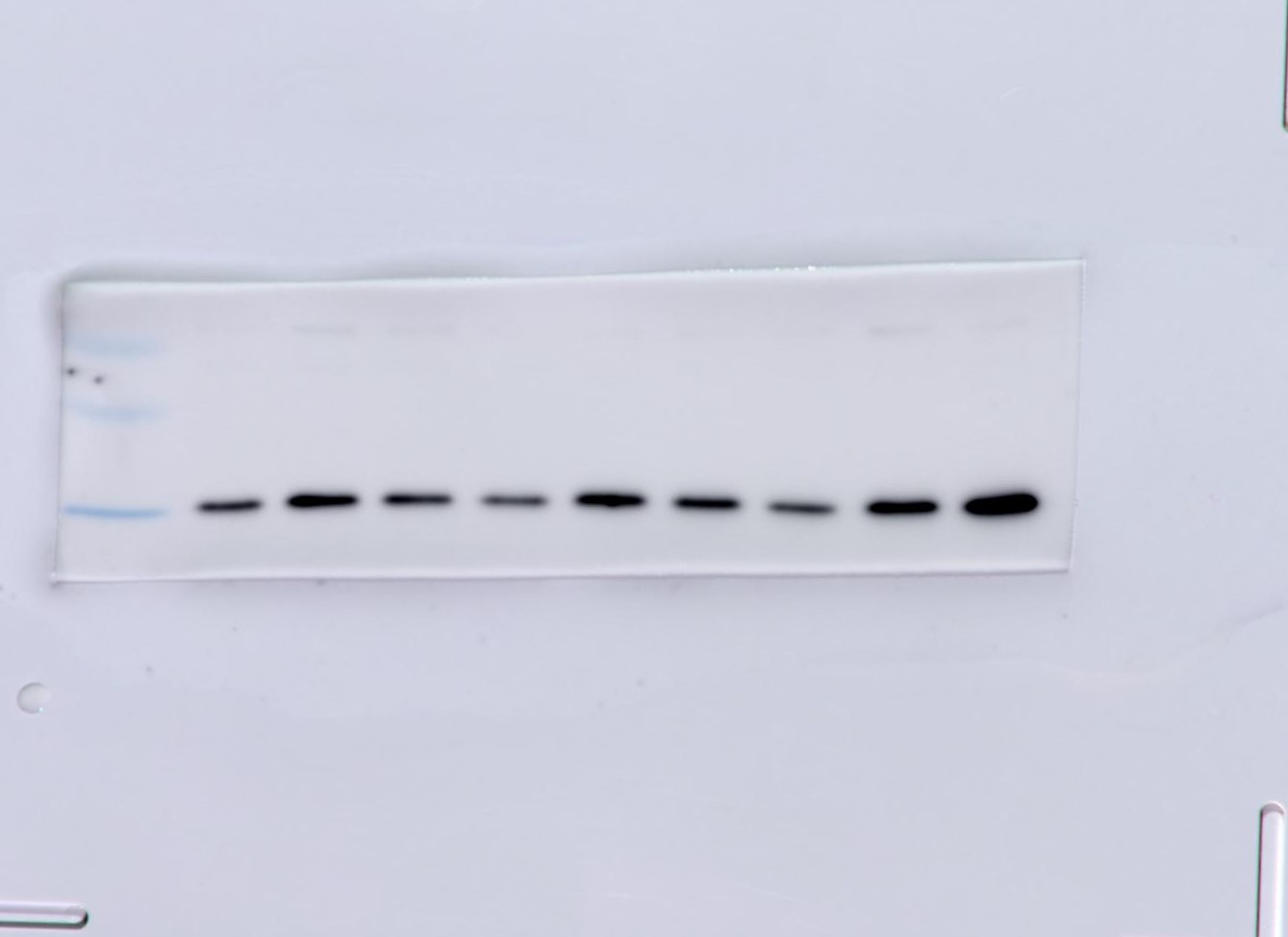

Supplement: Supplementary file 1 [file DataSheet1.zip › Western blotting-SMZJ/Western blotting-brain striatum-figure/iba1(16KD)-2.jpg]

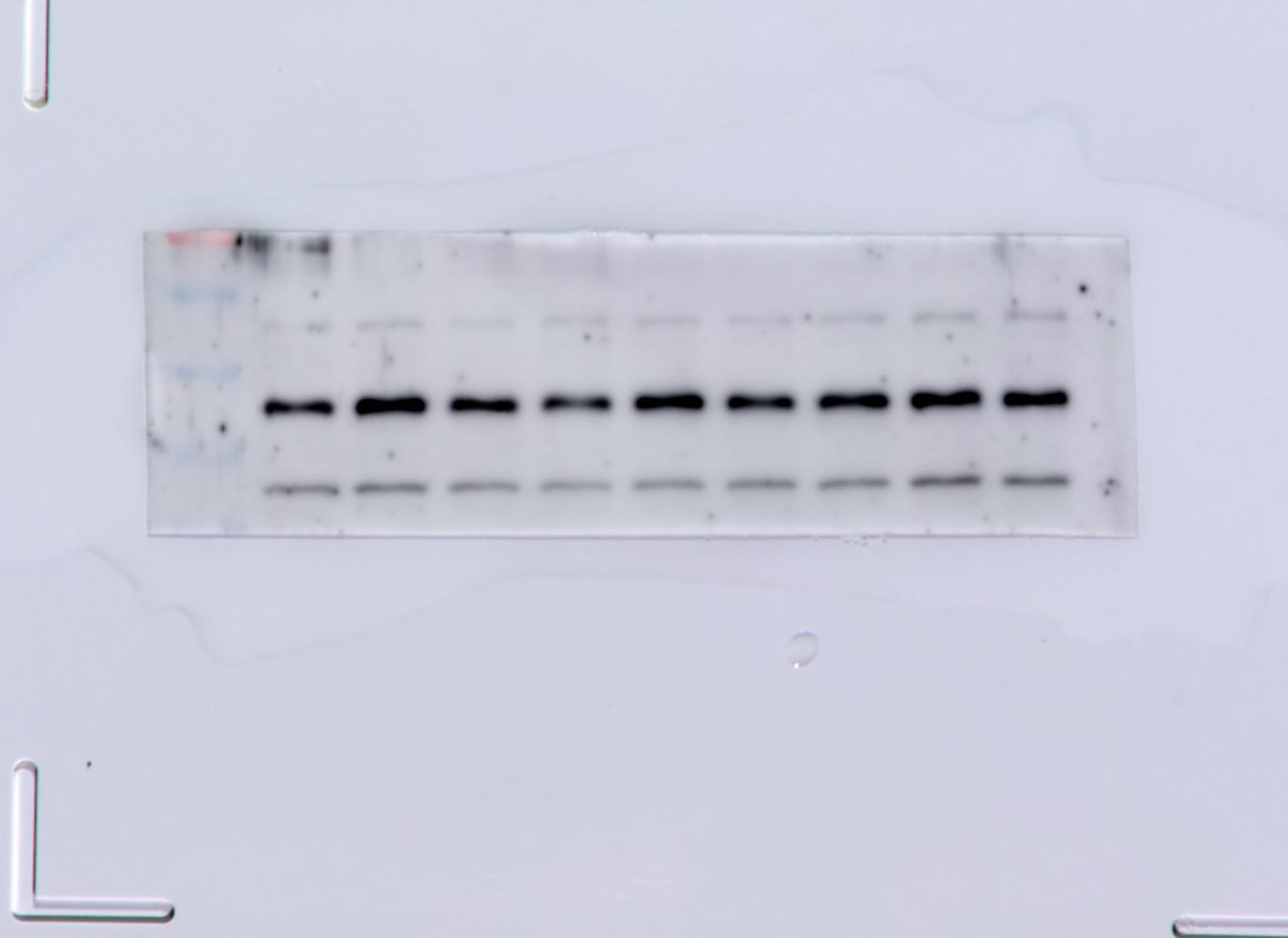

Supplement: Supplementary file 1 [file DataSheet1.zip › Western blotting-SMZJ/Western blotting-brain striatum-figure/IKBA(36KD)-1.jpg]

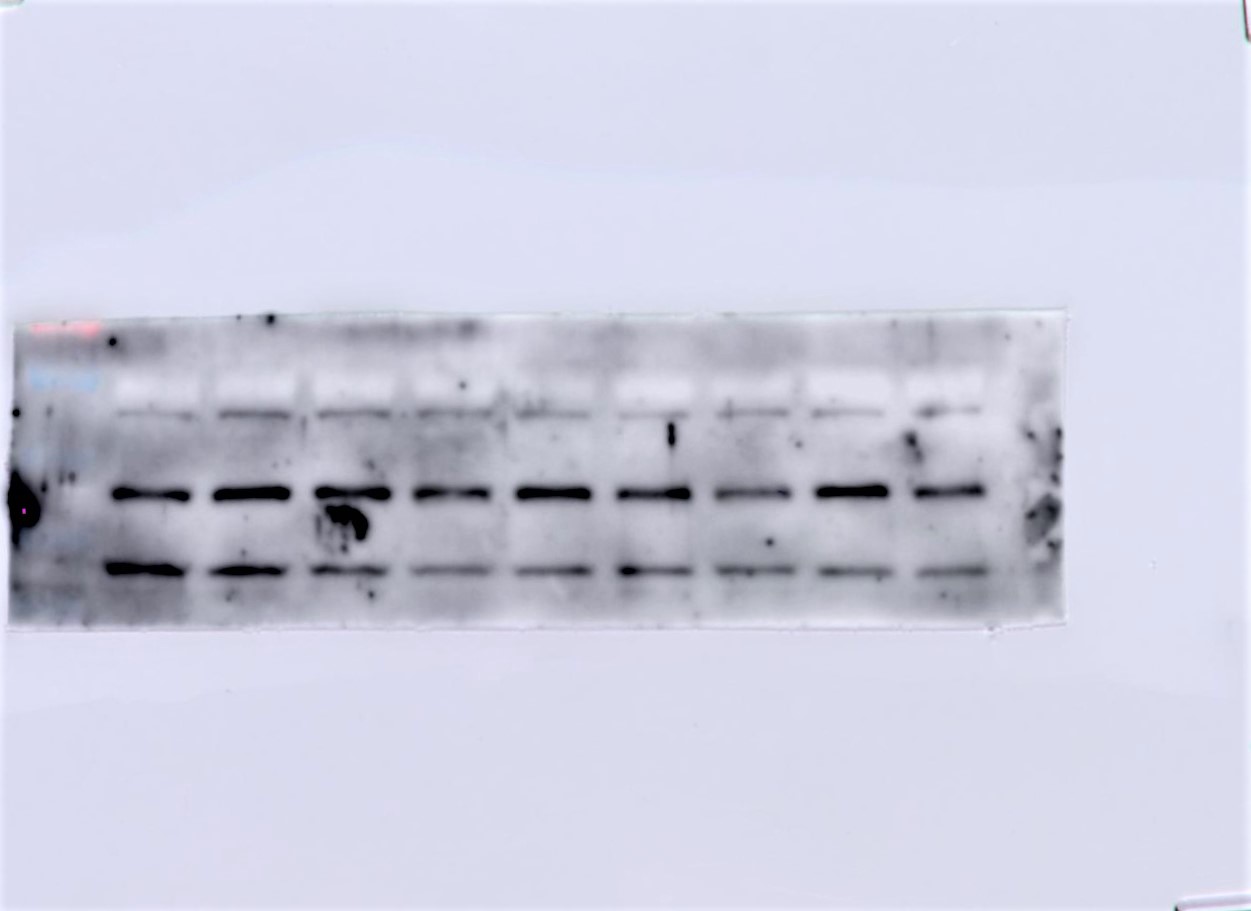

Supplement: Supplementary file 1 [file DataSheet1.zip › Western blotting-SMZJ/Western blotting-brain striatum-figure/IKBA(36KD)-2.jpg]

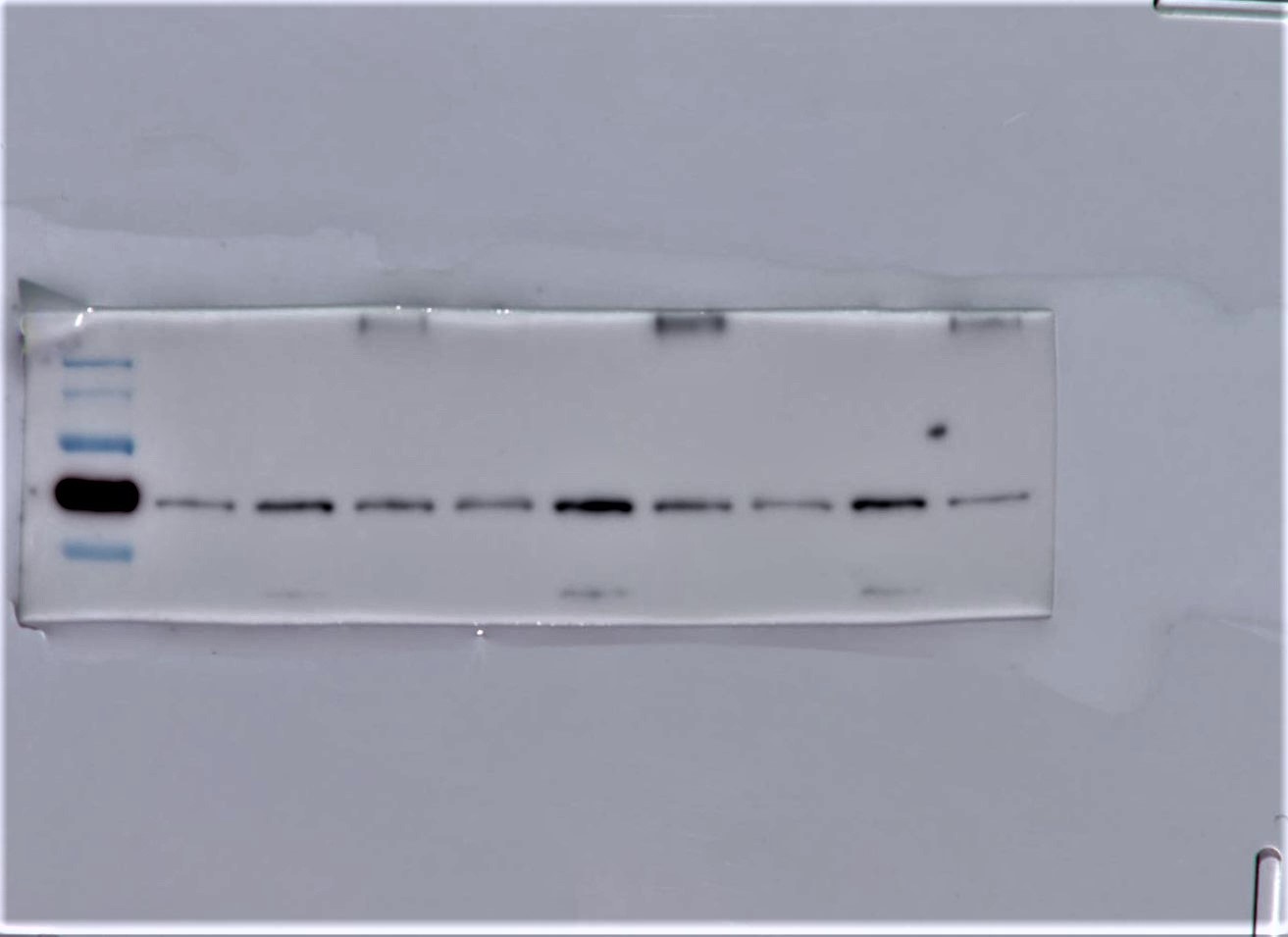

Supplement: Supplementary file 1 [file DataSheet1.zip › Western blotting-SMZJ/Western blotting-brain striatum-figure/INOS(65-70KD)-1.jpg]

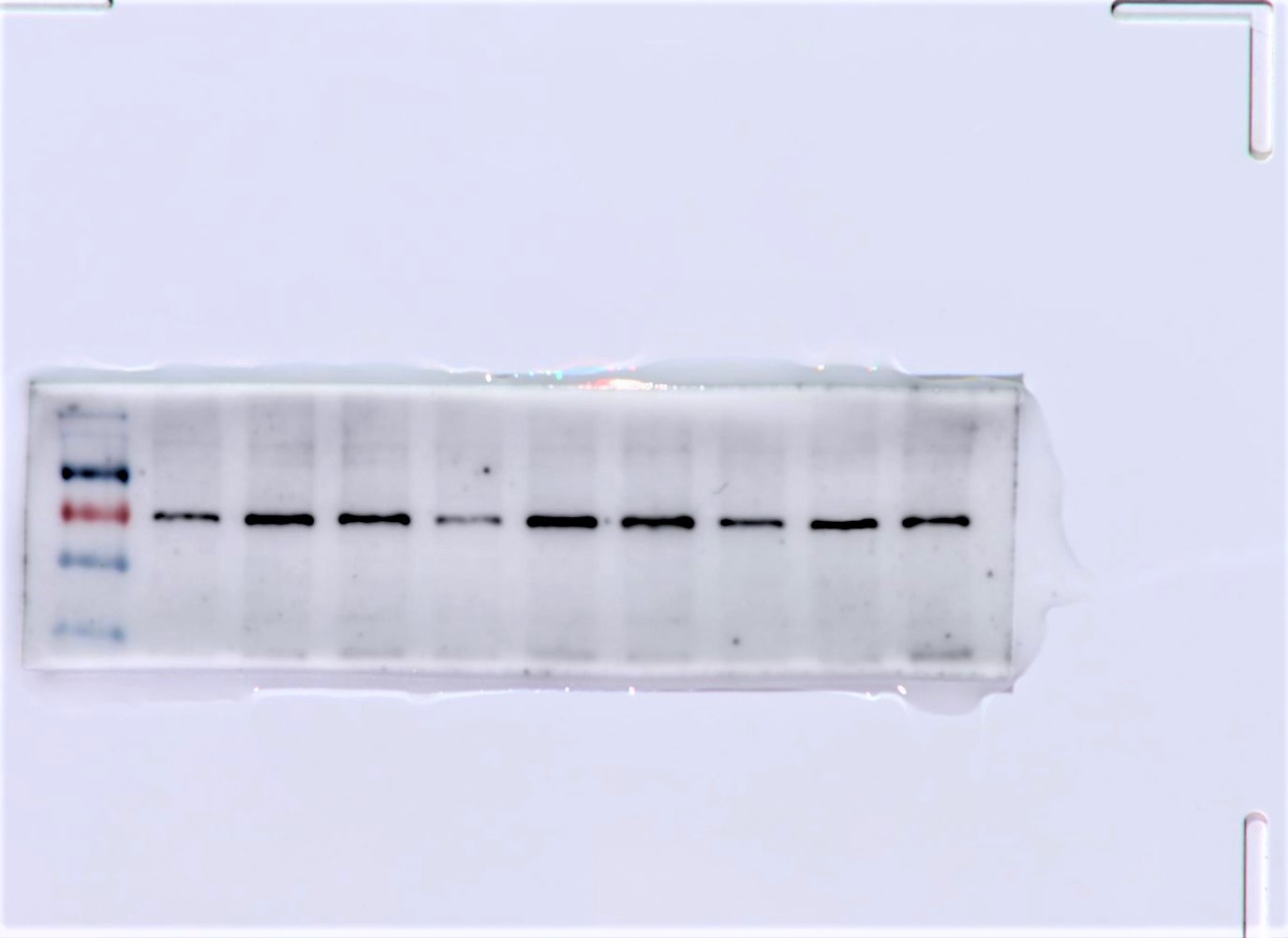

Supplement: Supplementary file 1 [file DataSheet1.zip › Western blotting-SMZJ/Western blotting-brain striatum-figure/INOS(65-70KD)-2.jpg]

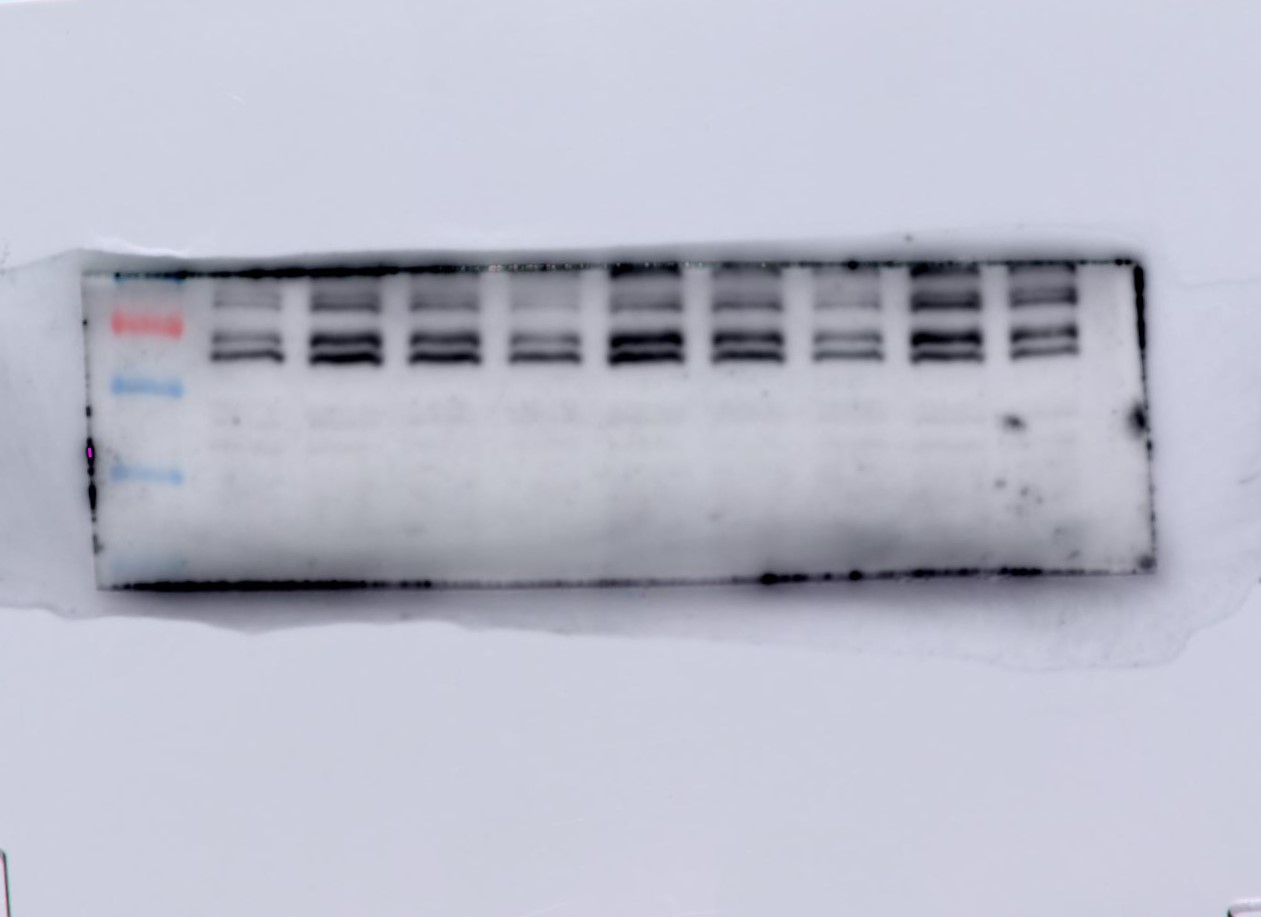

Supplement: Supplementary file 1 [file DataSheet1.zip › Western blotting-SMZJ/Western blotting-brain striatum-figure/P-P65(61KD)-1.jpg]

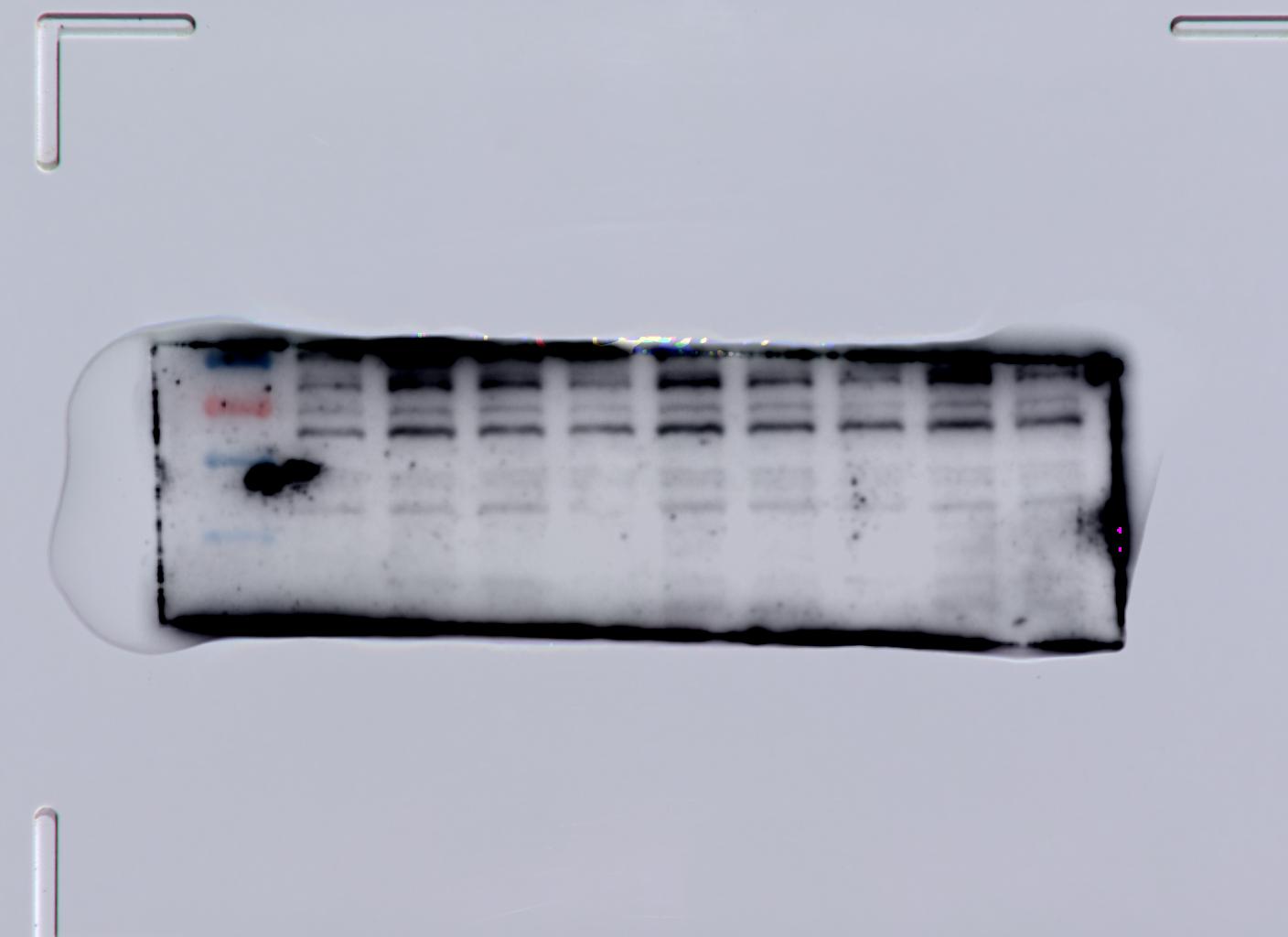

Supplement: Supplementary file 1 [file DataSheet1.zip › Western blotting-SMZJ/Western blotting-brain striatum-figure/P-P65(61KD)-2.jpg]

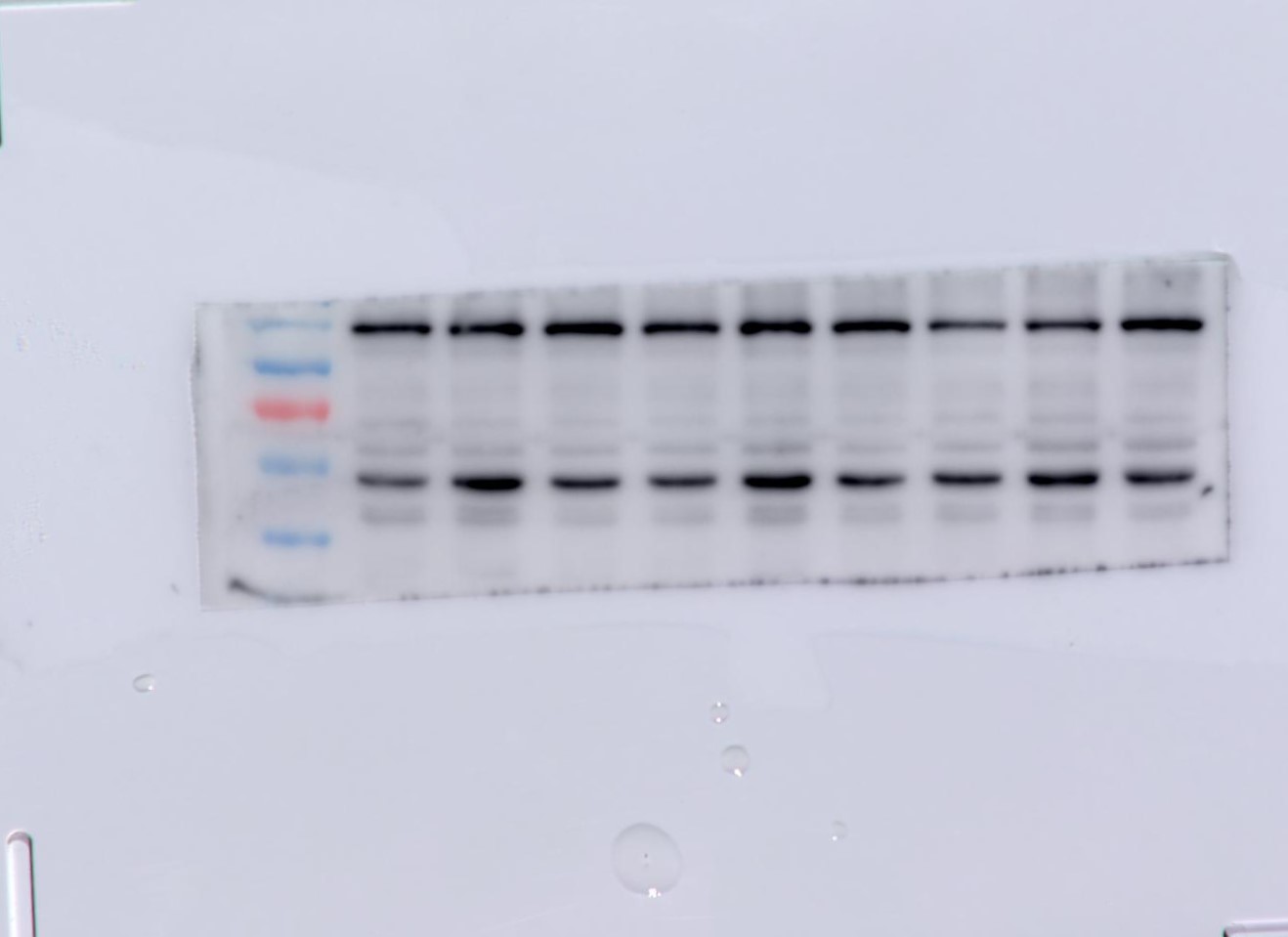

Supplement: Supplementary file 1 [file DataSheet1.zip › Western blotting-SMZJ/Western blotting-brain striatum-figure/P-PAKT(56KD)-1.jpg]

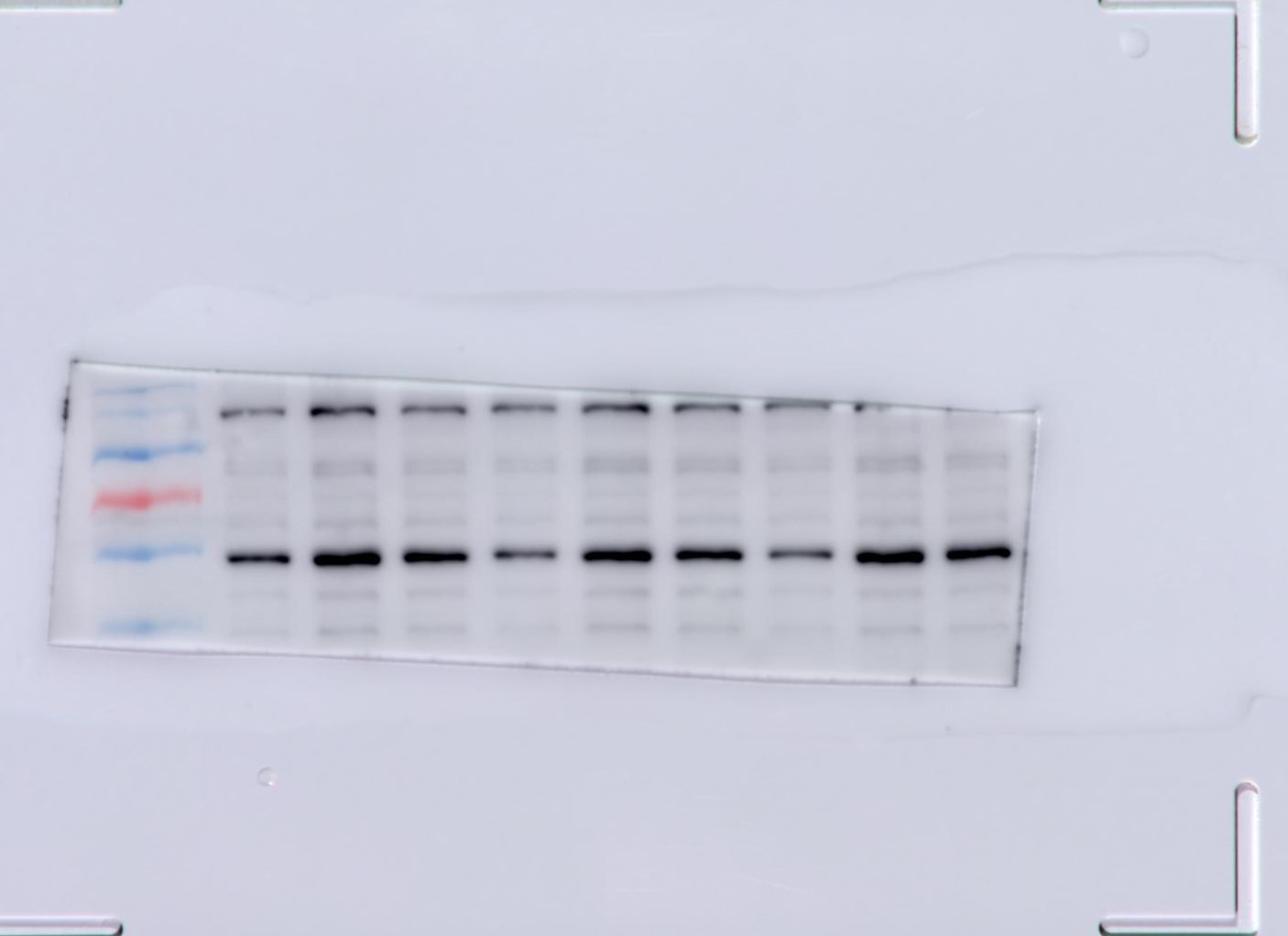

Supplement: Supplementary file 1 [file DataSheet1.zip › Western blotting-SMZJ/Western blotting-brain striatum-figure/P-PAKT(56KD)-2.jpg]

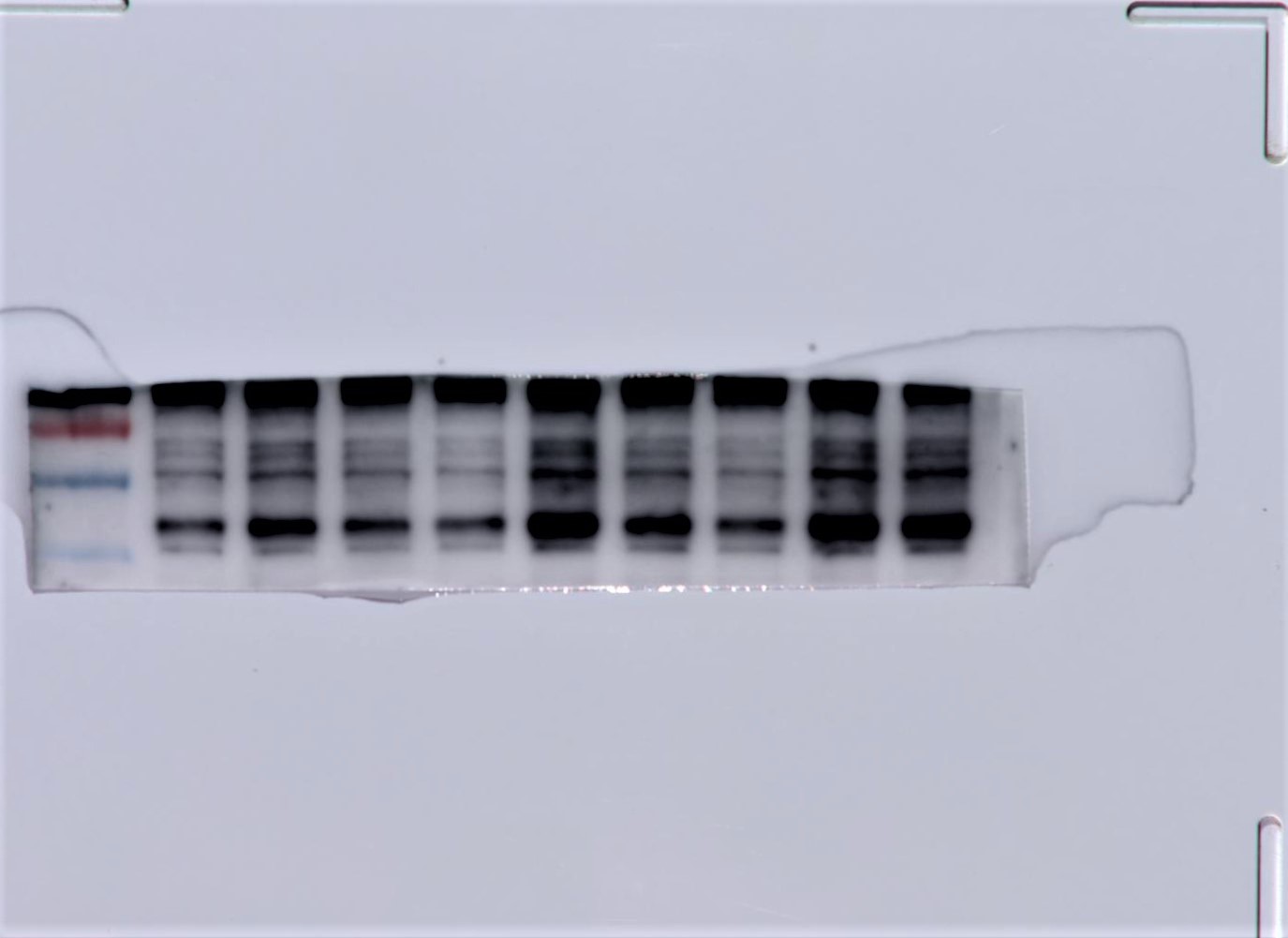

Supplement: Supplementary file 1 [file DataSheet1.zip › Western blotting-SMZJ/Western blotting-brain striatum-figure/P65(65KD)-1.jpg]

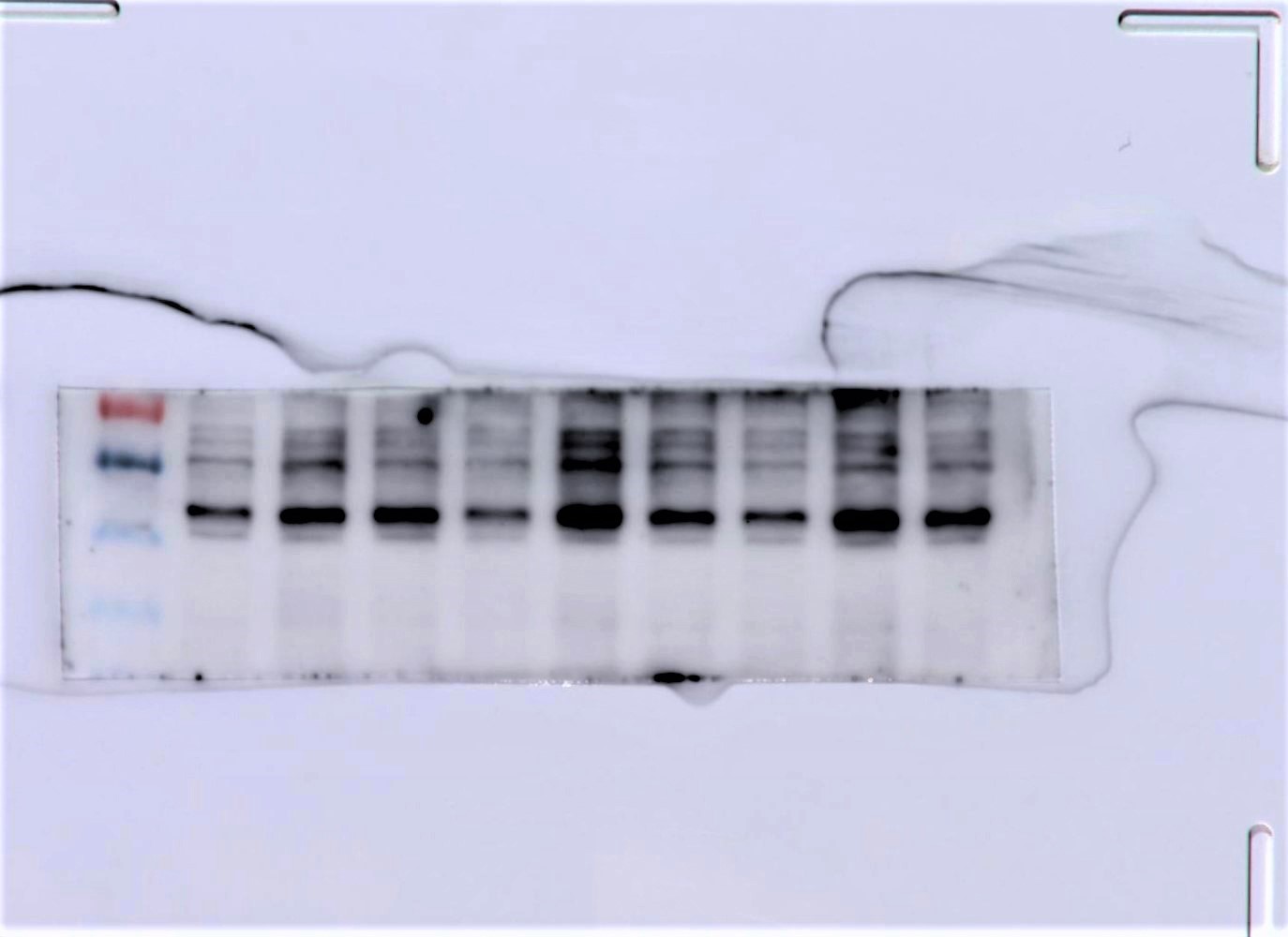

Supplement: Supplementary file 1 [file DataSheet1.zip › Western blotting-SMZJ/Western blotting-brain striatum-figure/P65(65KD)-2.jpg]

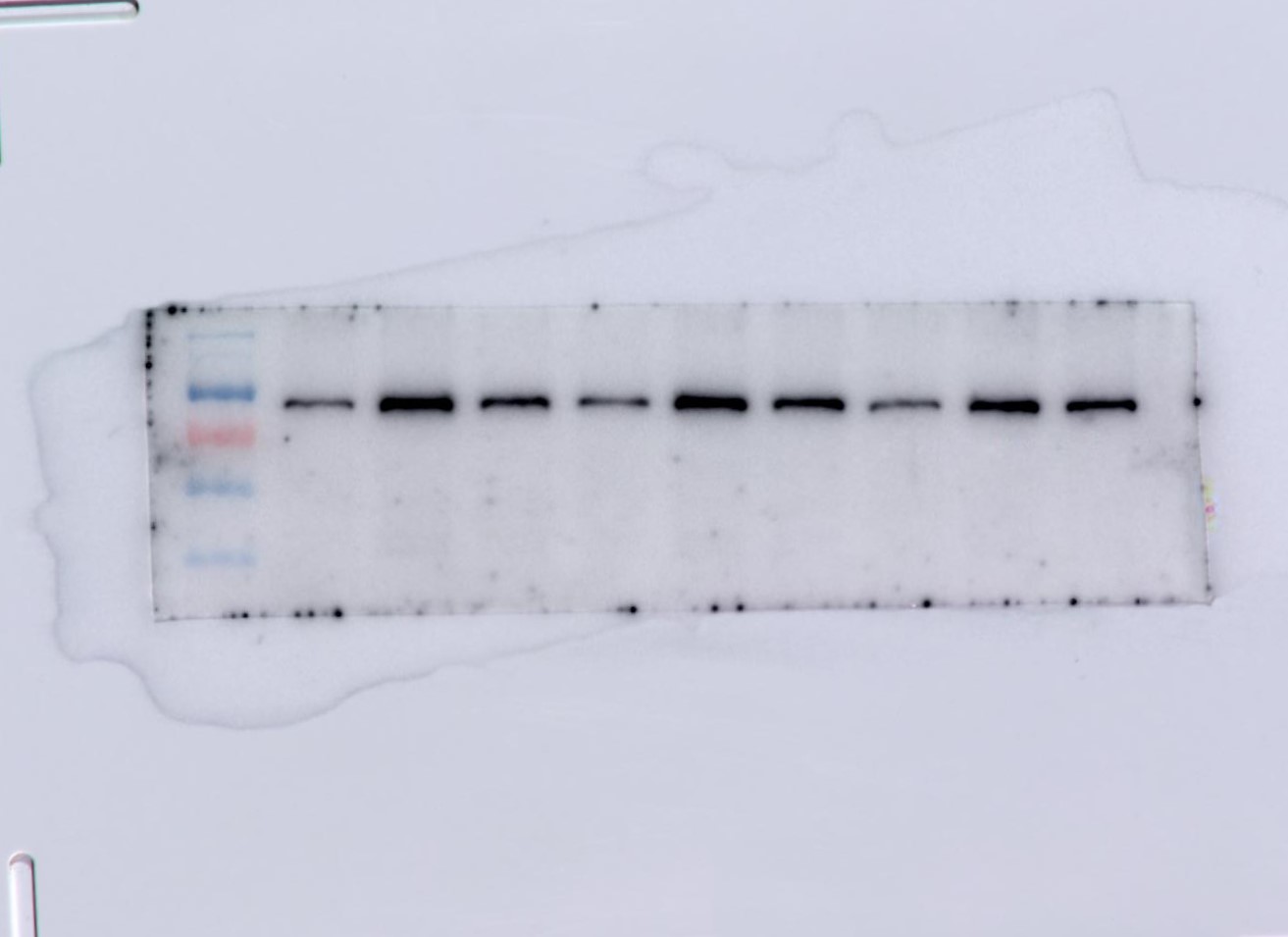

Supplement: Supplementary file 1 [file DataSheet1.zip › Western blotting-SMZJ/Western blotting-brain striatum-figure/PI3K(85KD)-1.jpg]

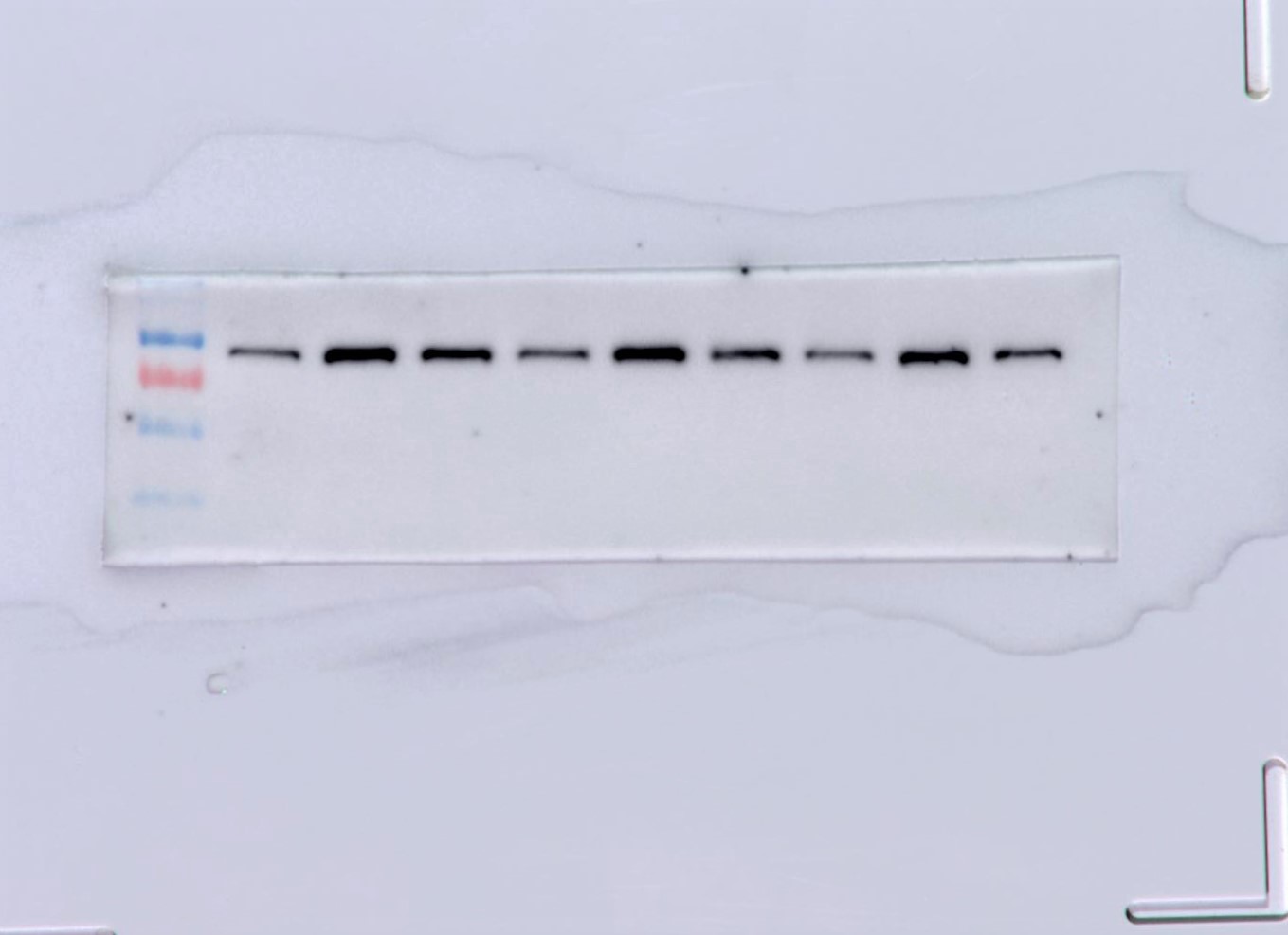

Supplement: Supplementary file 1 [file DataSheet1.zip › Western blotting-SMZJ/Western blotting-brain striatum-figure/PI3K(85KD)-2.jpg]

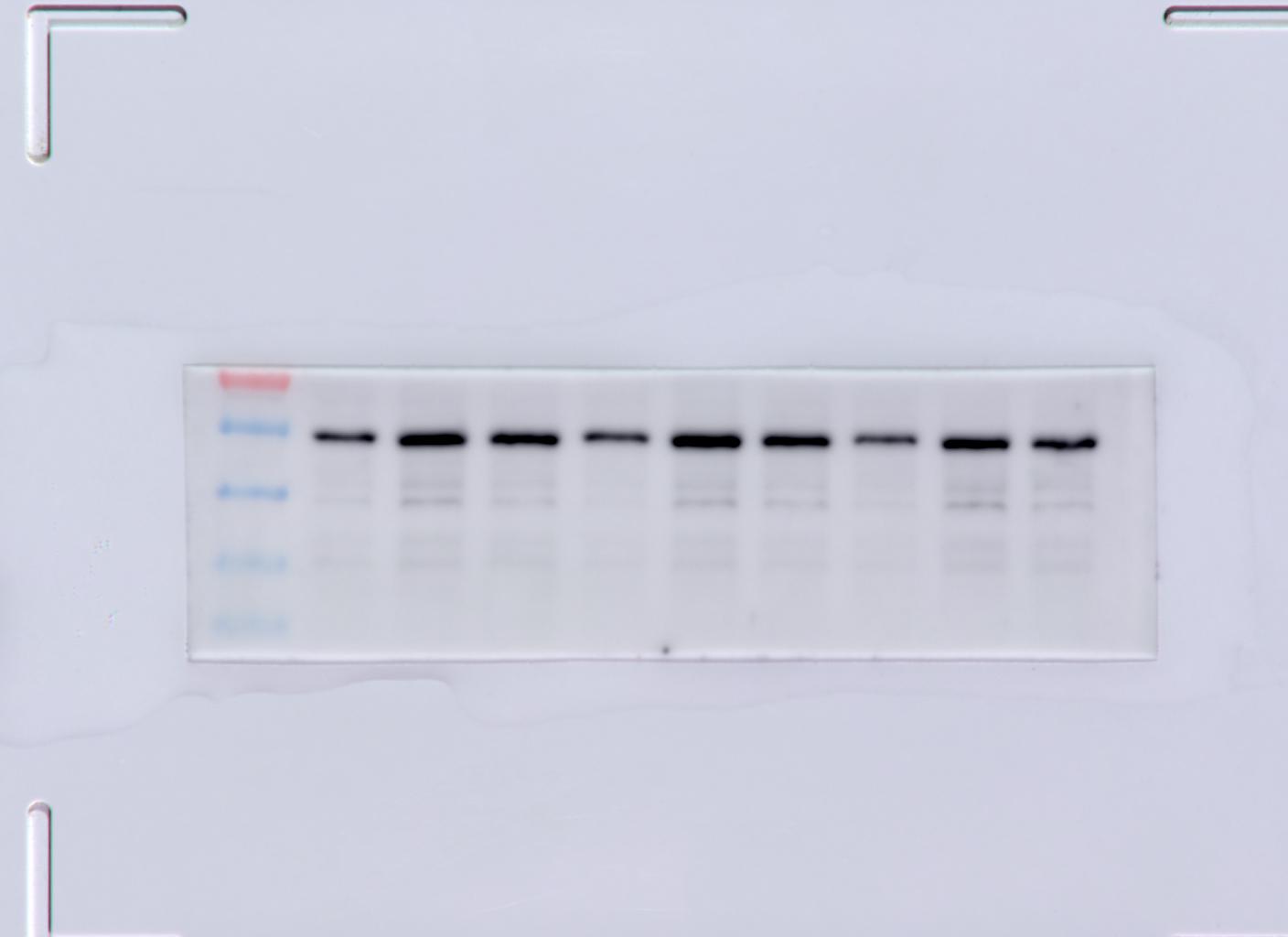

Supplement: Supplementary file 1 [file DataSheet1.zip › Western blotting-SMZJ/Western blotting-brain striatum-figure/PKA(45KD)-1.jpg]

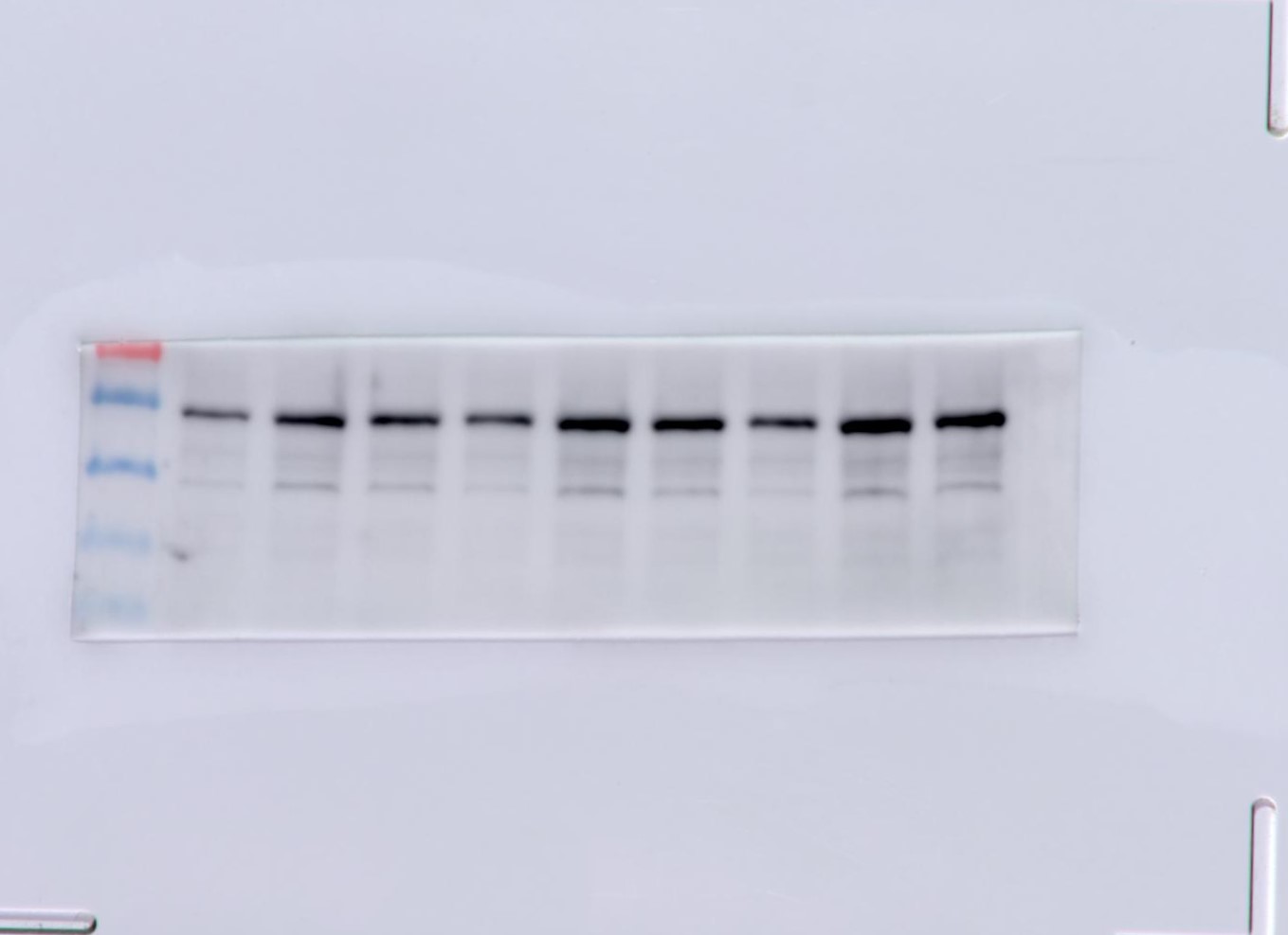

Supplement: Supplementary file 1 [file DataSheet1.zip › Western blotting-SMZJ/Western blotting-brain striatum-figure/PKA(45KD)-2.jpg]

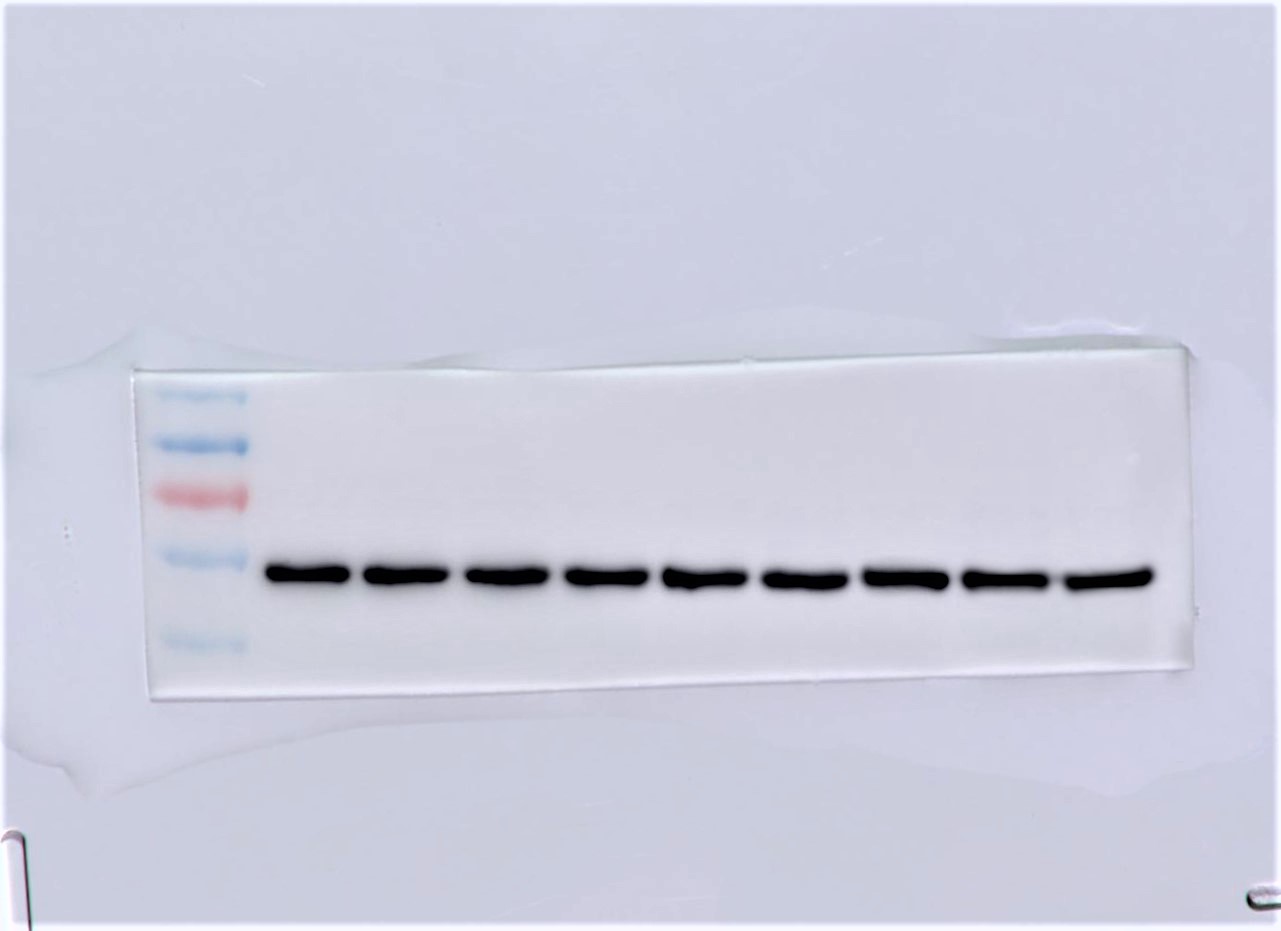

Supplement: Supplementary file 1 [file DataSheet1.zip › Western blotting-SMZJ/Western blotting-brain striatum-figure/β-tubulin(55KD)-1.jpg]

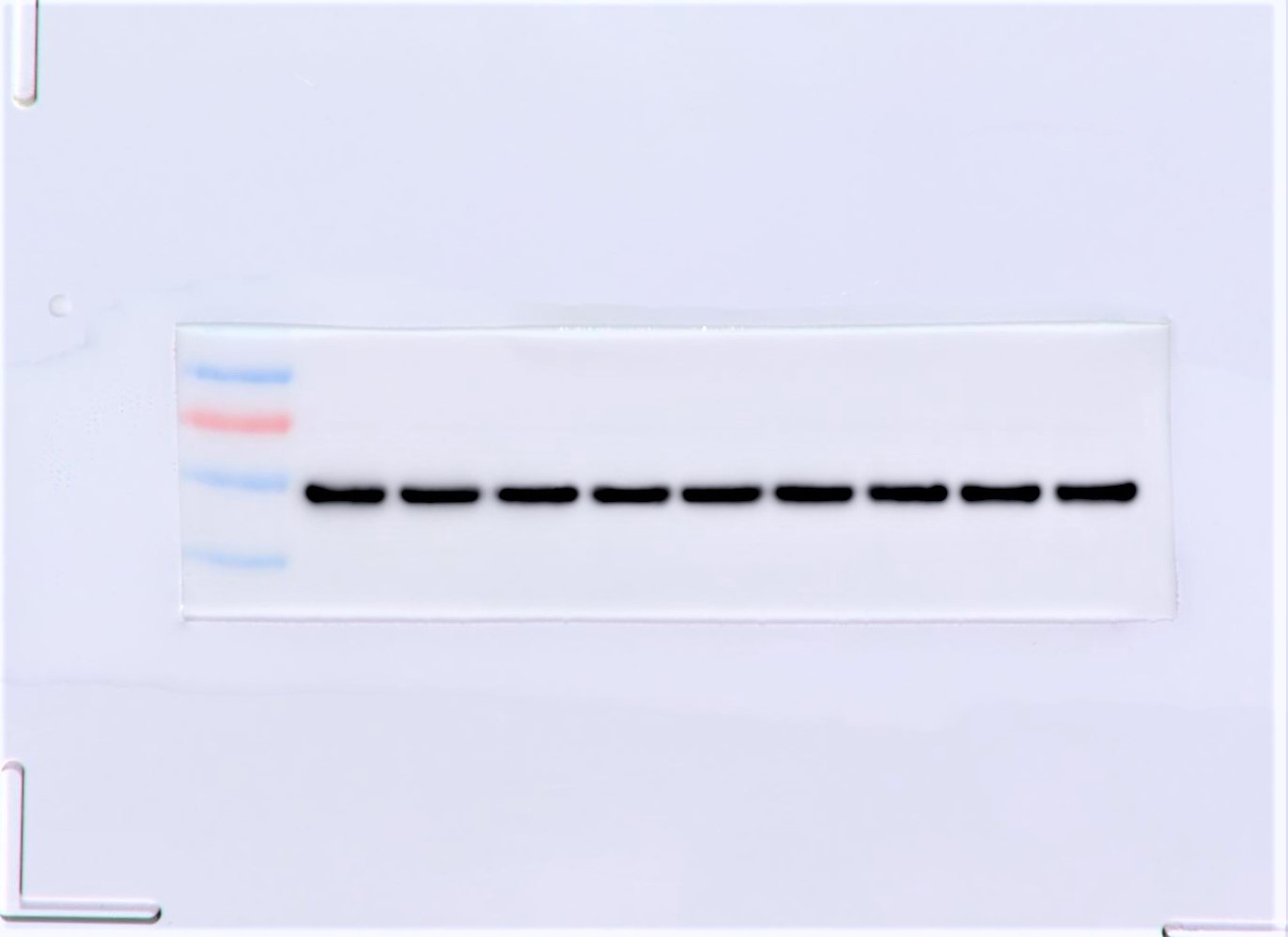

Supplement: Supplementary file 1 [file DataSheet1.zip › Western blotting-SMZJ/Western blotting-brain striatum-figure/β-tubulin(55KD)-2.jpg]

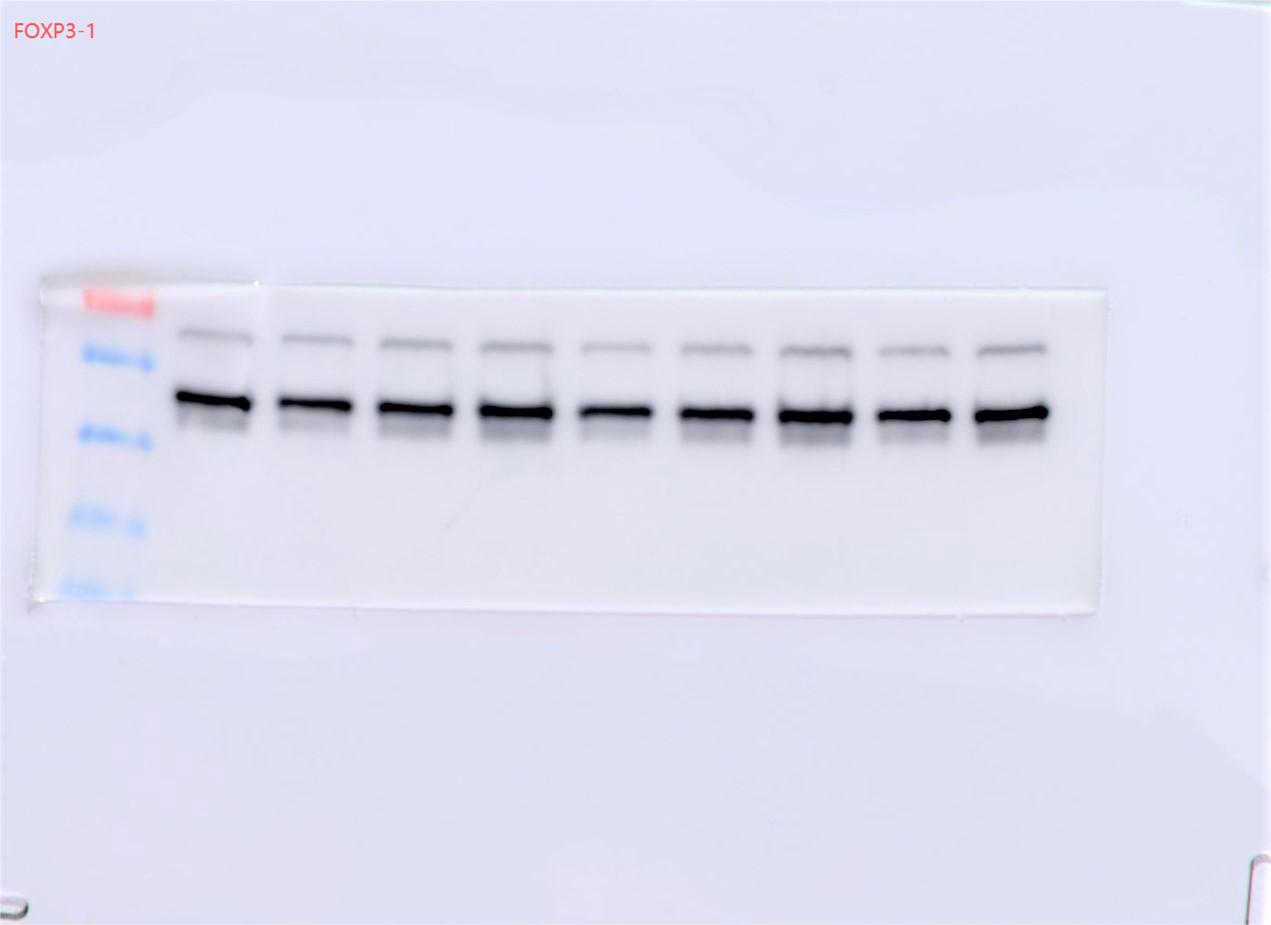

Supplement: Supplementary file 1 [file DataSheet1.zip › Western blotting-SMZJ/Western blotting-spleen-figure/foxp3(47KD)-1.jpg]

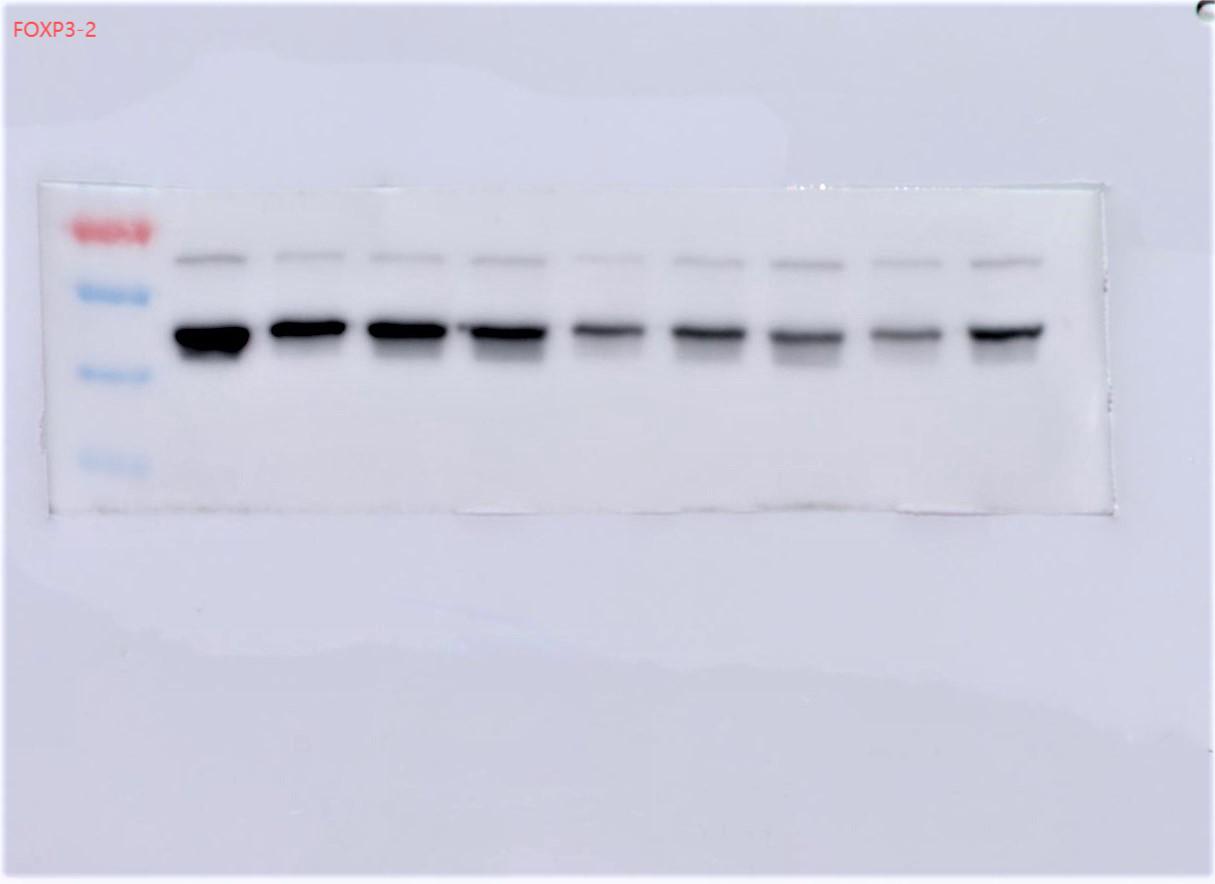

Supplement: Supplementary file 1 [file DataSheet1.zip › Western blotting-SMZJ/Western blotting-spleen-figure/foxp3(47KD)-2.jpg]

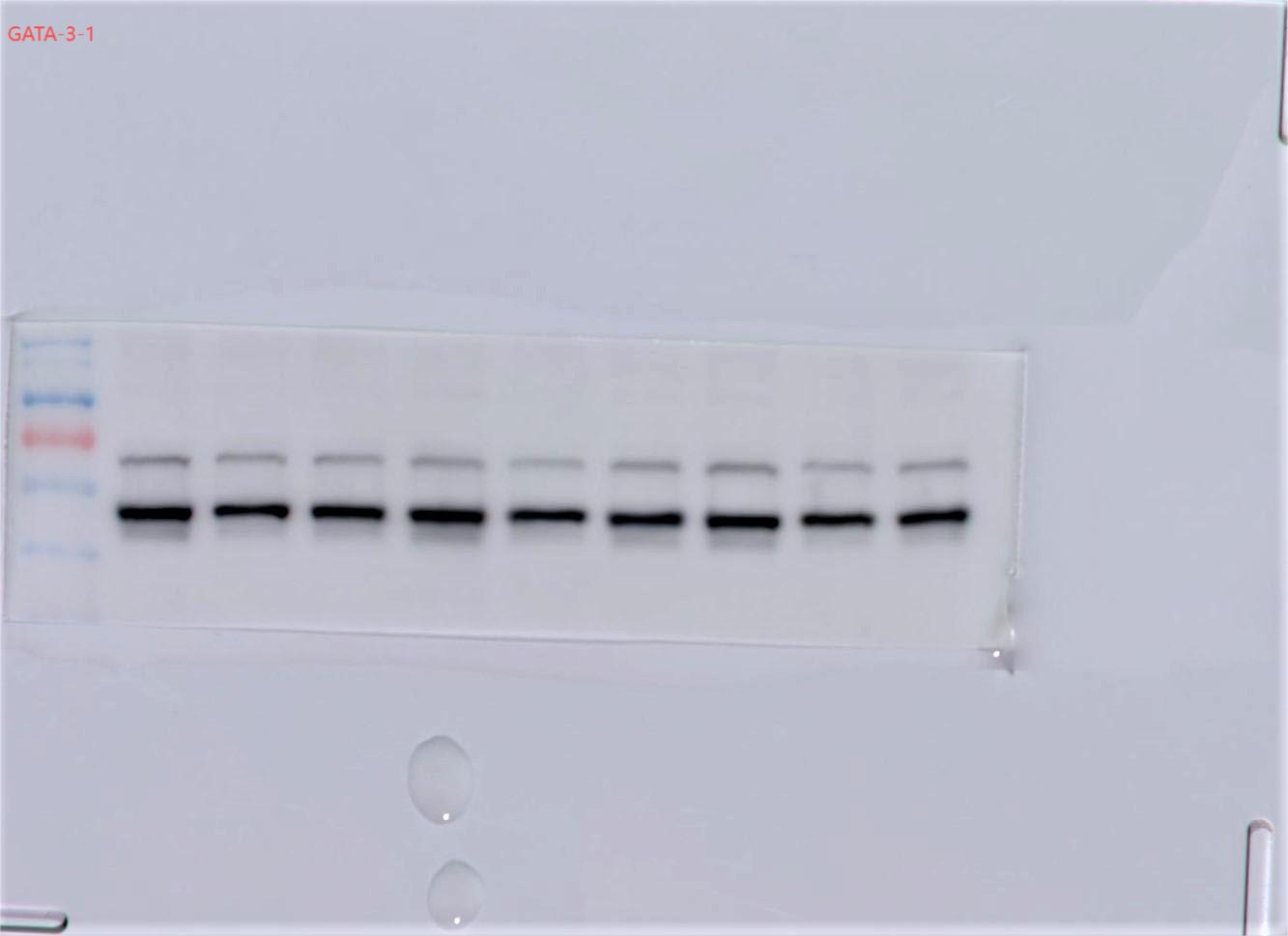

Supplement: Supplementary file 1 [file DataSheet1.zip › Western blotting-SMZJ/Western blotting-spleen-figure/GATA-3(48KD)-1.jpg]

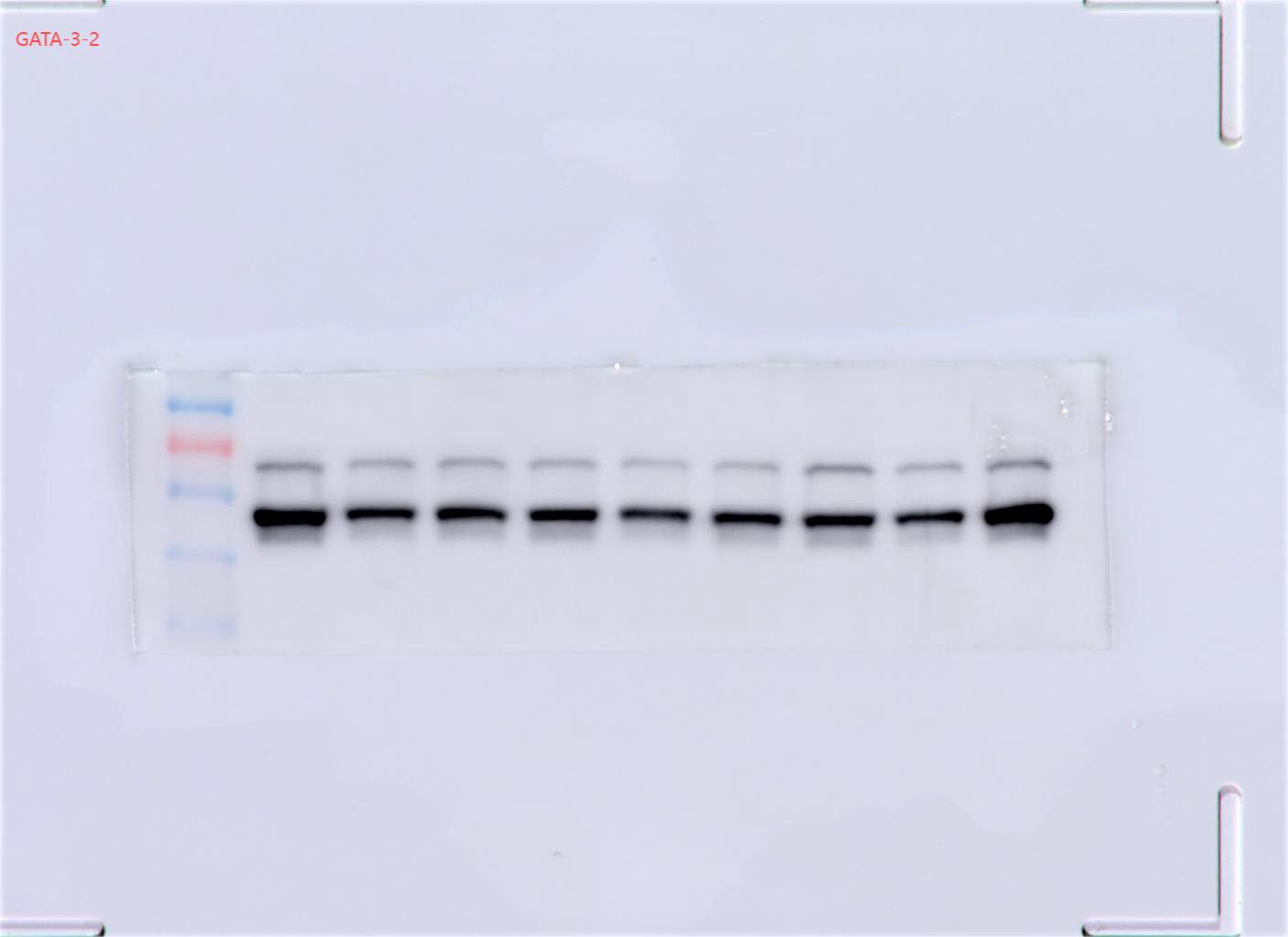

Supplement: Supplementary file 1 [file DataSheet1.zip › Western blotting-SMZJ/Western blotting-spleen-figure/GATA-3(48KD)-2.jpg]

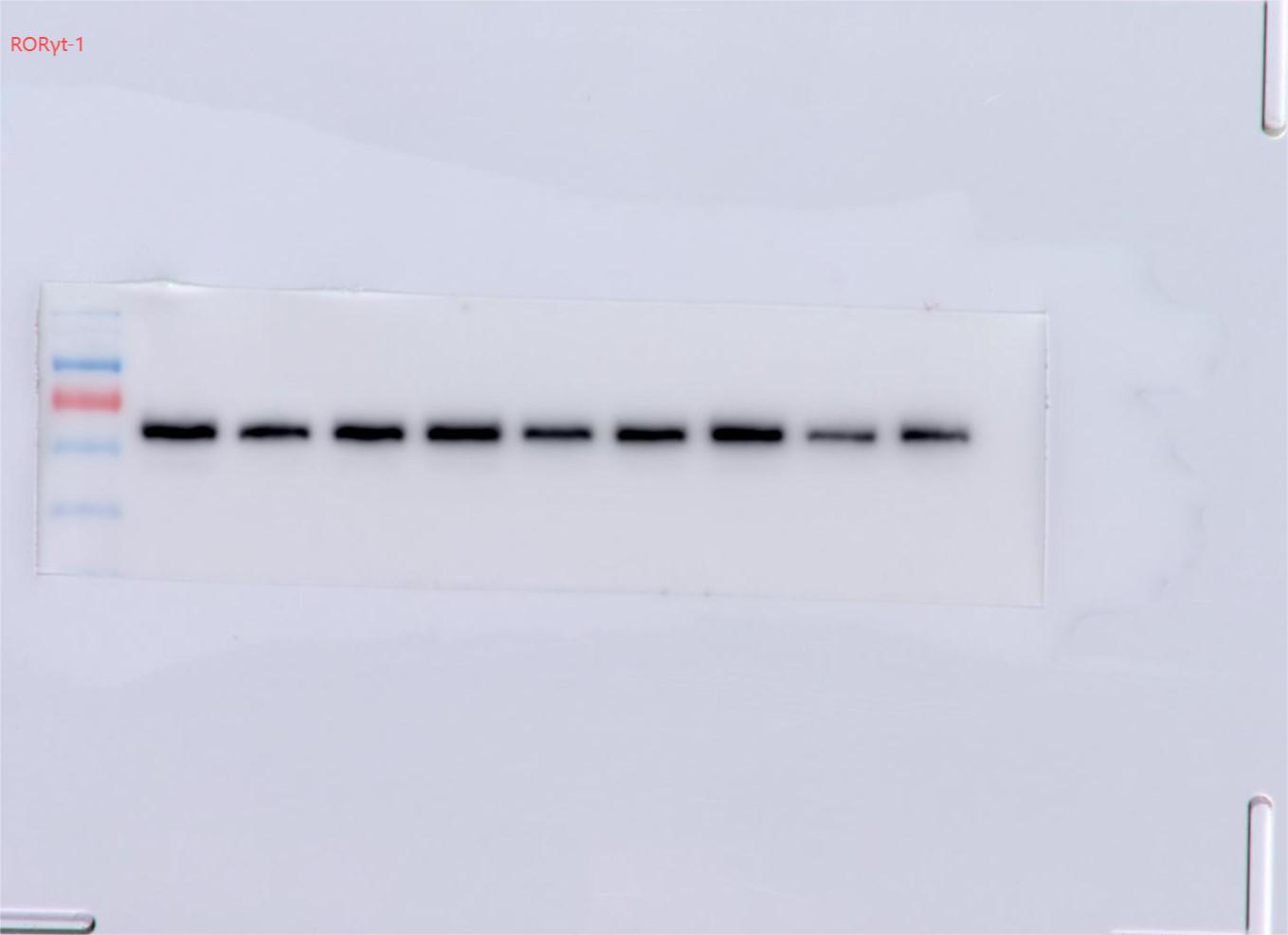

Supplement: Supplementary file 1 [file DataSheet1.zip › Western blotting-SMZJ/Western blotting-spleen-figure/roryt(58KD)-1.jpg]

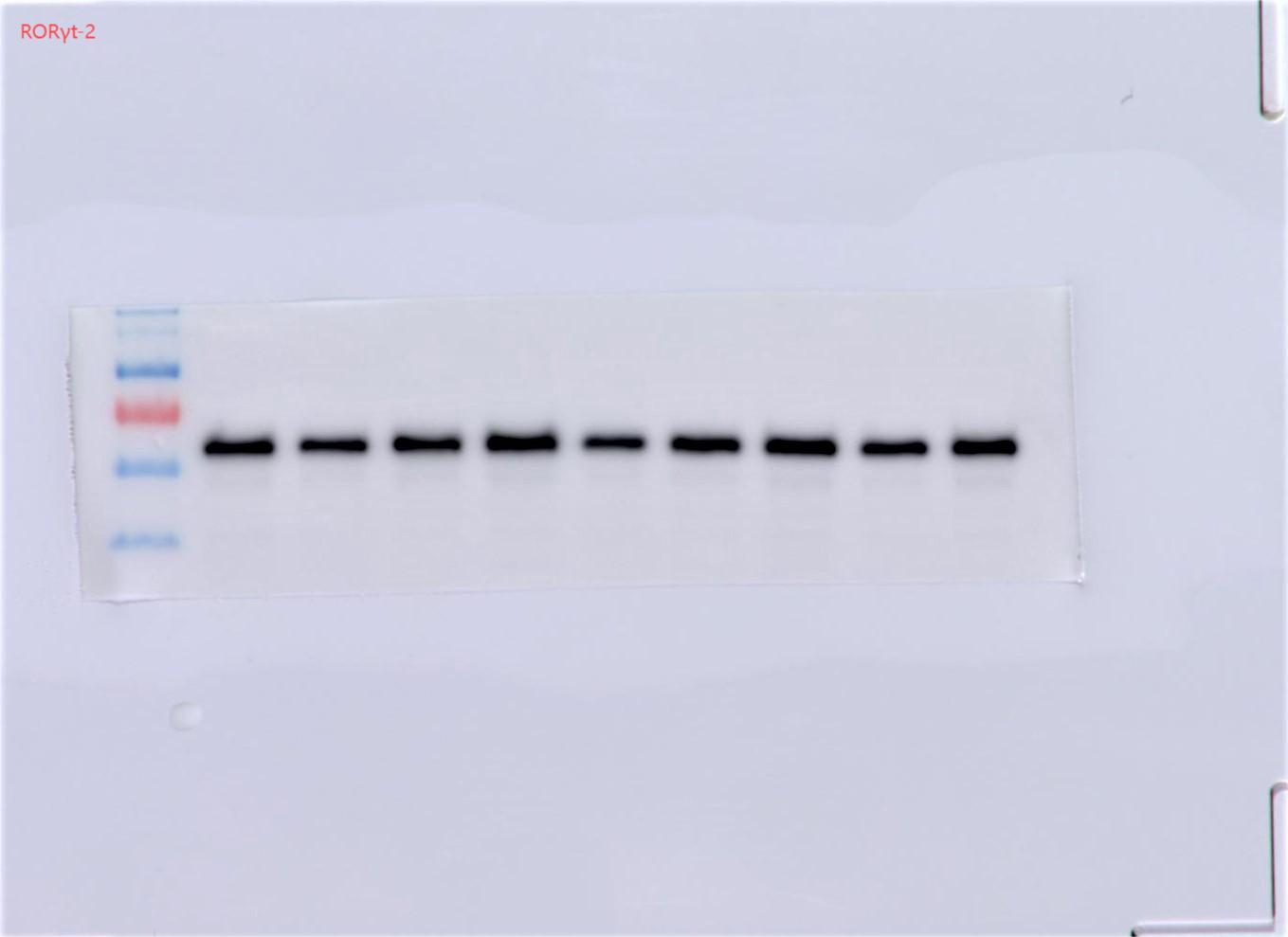

Supplement: Supplementary file 1 [file DataSheet1.zip › Western blotting-SMZJ/Western blotting-spleen-figure/roryt(58KD)-2.jpg]

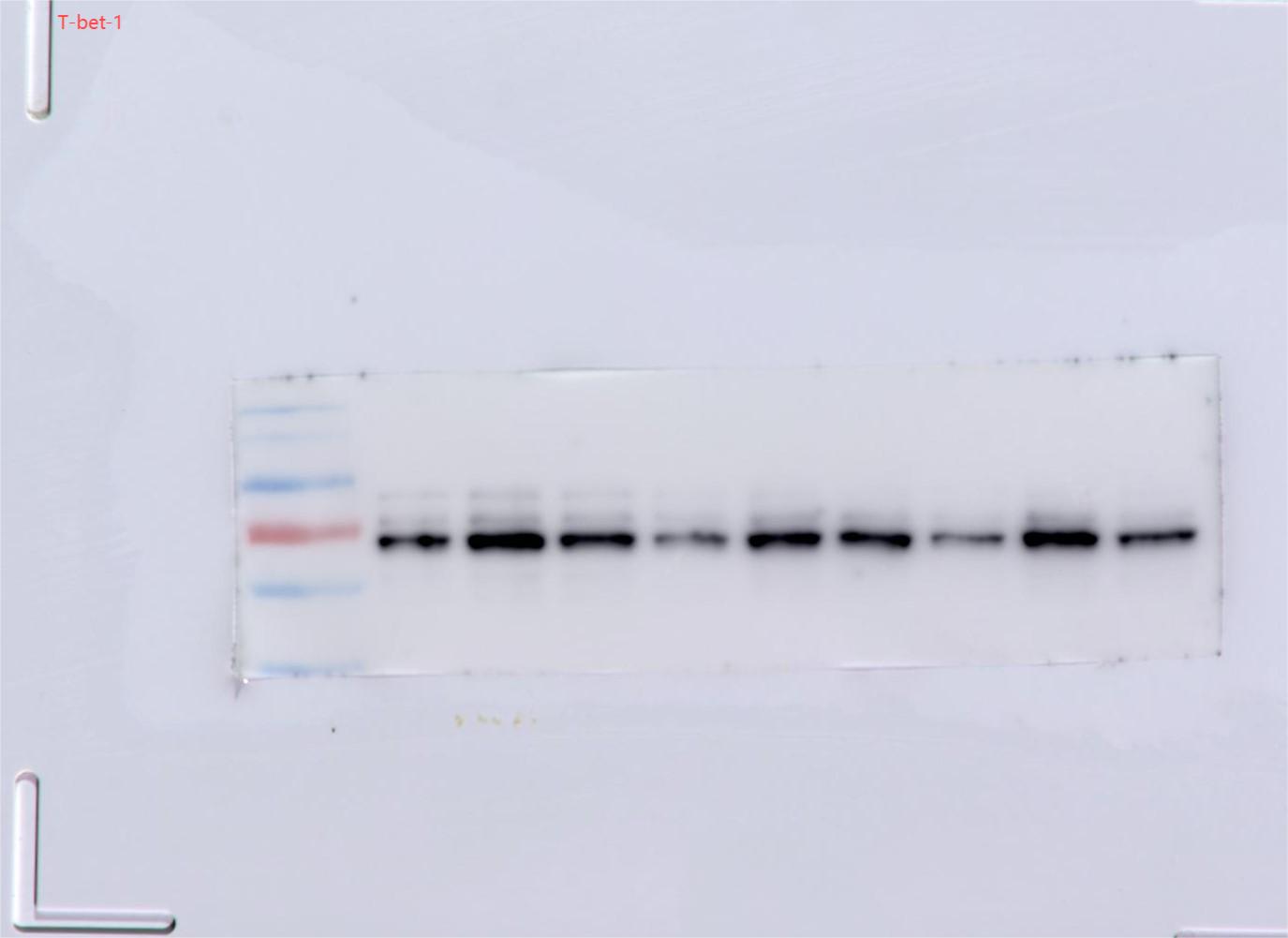

Supplement: Supplementary file 1 [file DataSheet1.zip › Western blotting-SMZJ/Western blotting-spleen-figure/T-BET(56-68KD)-1.jpg]

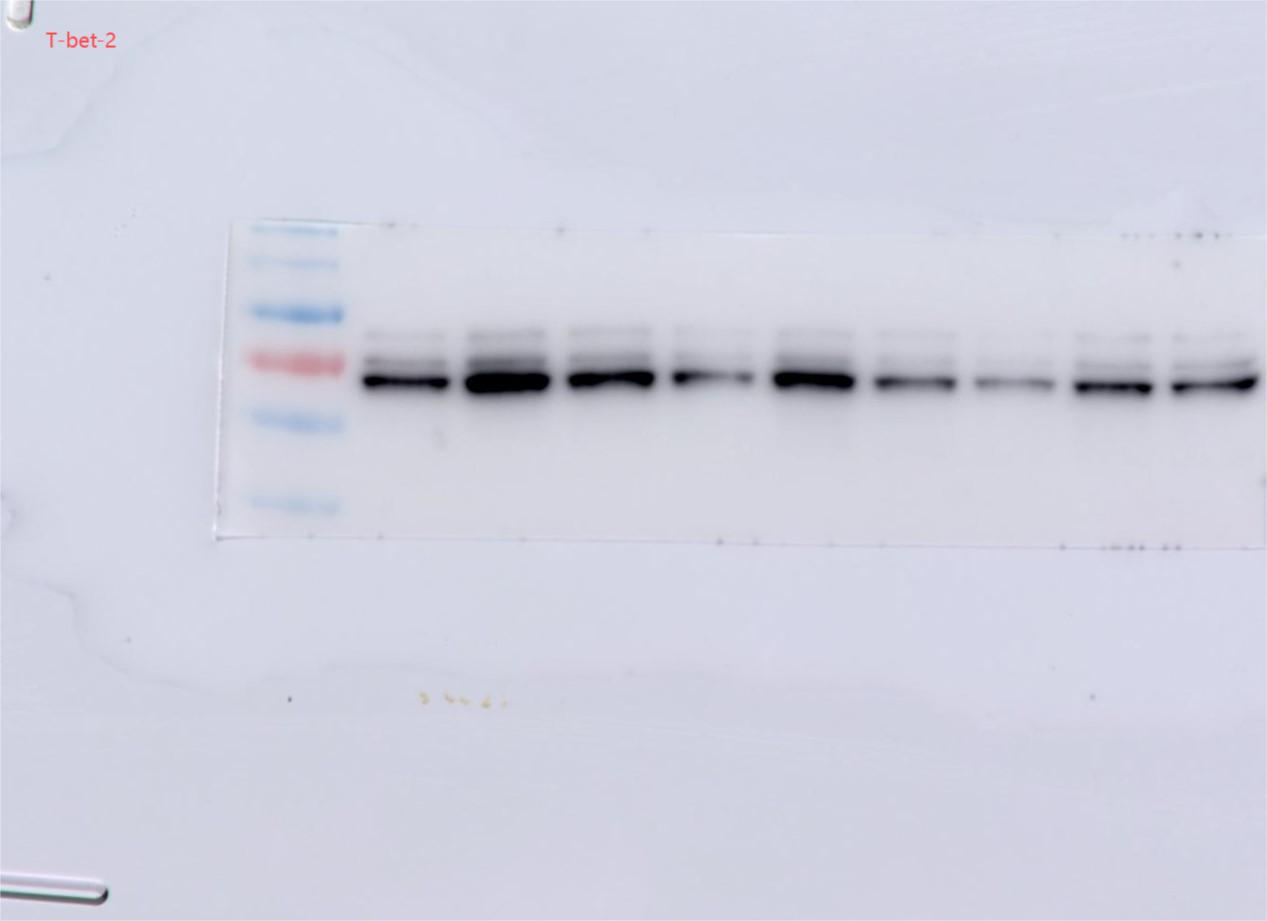

Supplement: Supplementary file 1 [file DataSheet1.zip › Western blotting-SMZJ/Western blotting-spleen-figure/T-BET(56-68KD)-2.jpg]

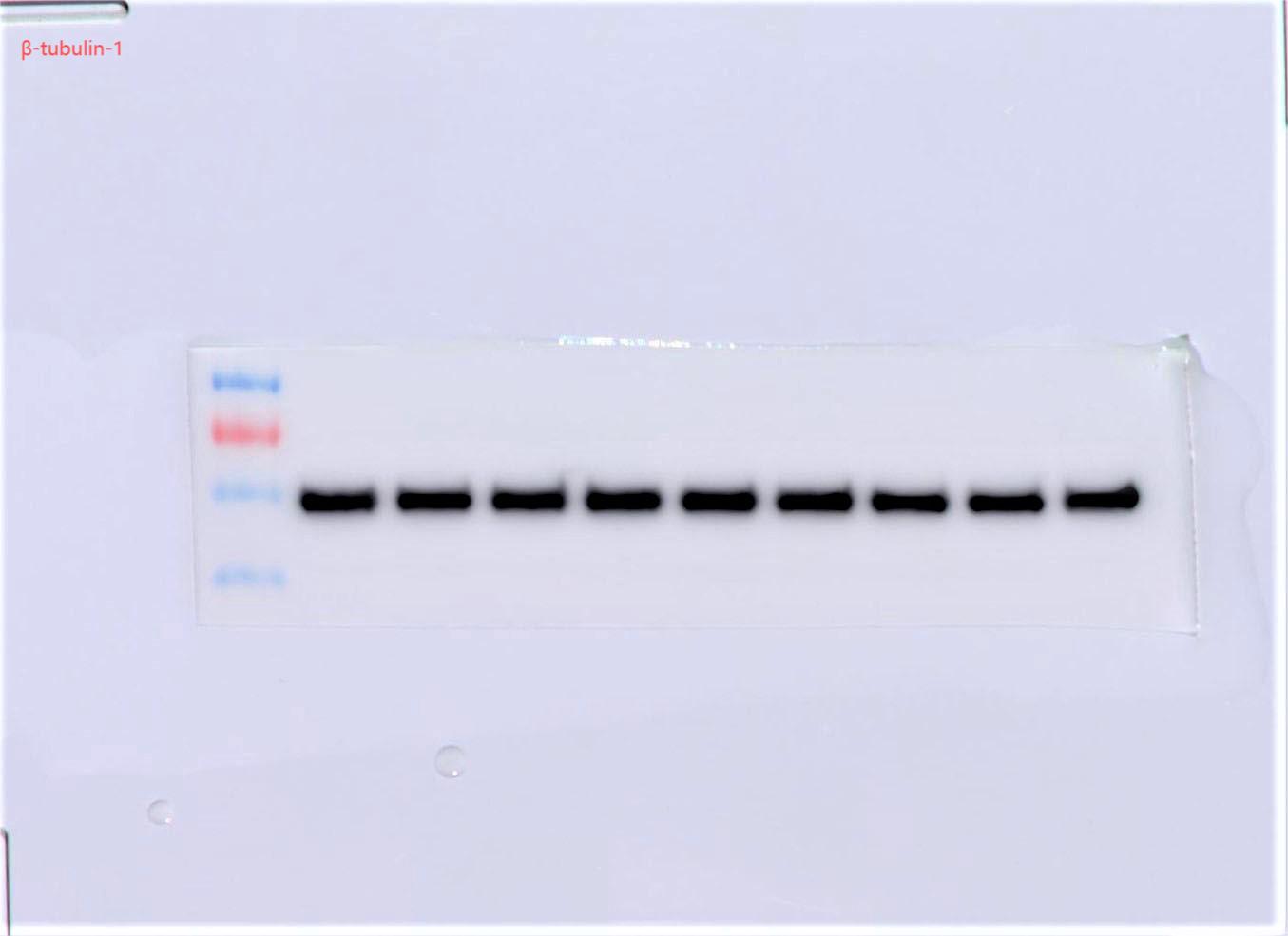

Supplement: Supplementary file 1 [file DataSheet1.zip › Western blotting-SMZJ/Western blotting-spleen-figure/β-tubulin(55KD)-1-spleen.jpg]

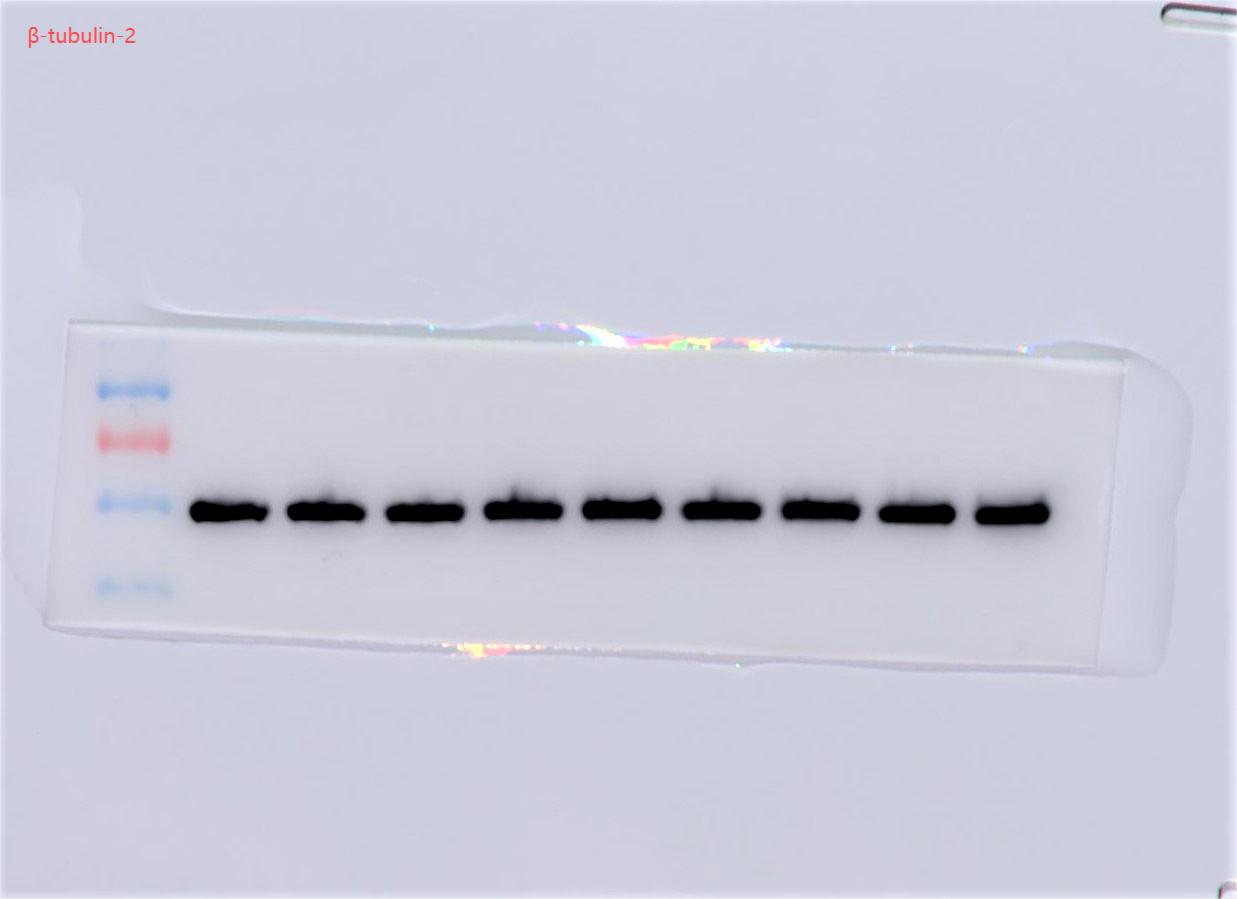

Supplement: Supplementary file 1 [file DataSheet1.zip › Western blotting-SMZJ/Western blotting-spleen-figure/β-tubulin(55KD)-2-spleen.jpg]

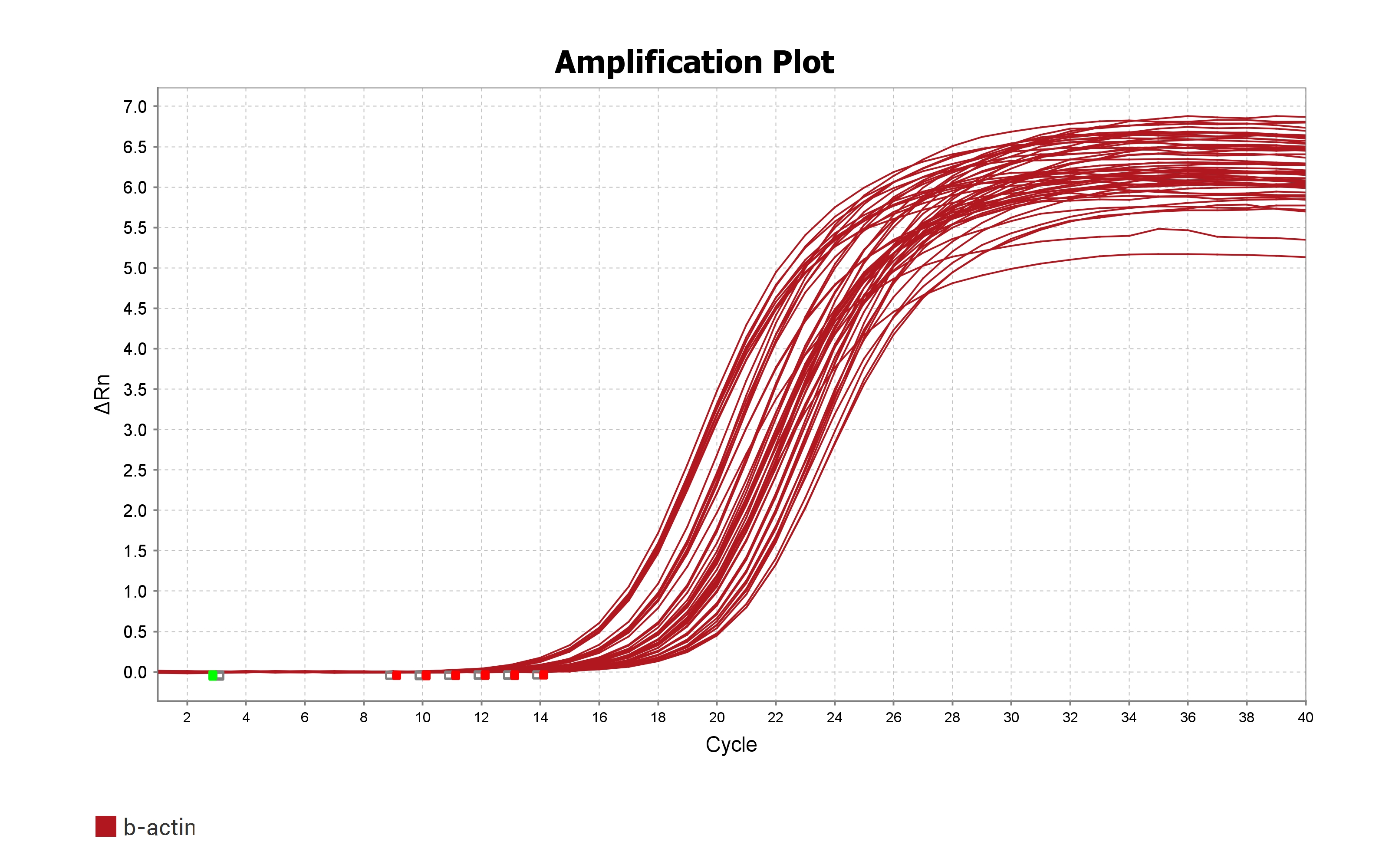

Supplement: Supplementary file 2 [file DataSheet2.zip › qRT-PCR-MZJ-2025-09/Curve/Amplification Plot lixinxin xibao b-actin .jpg]

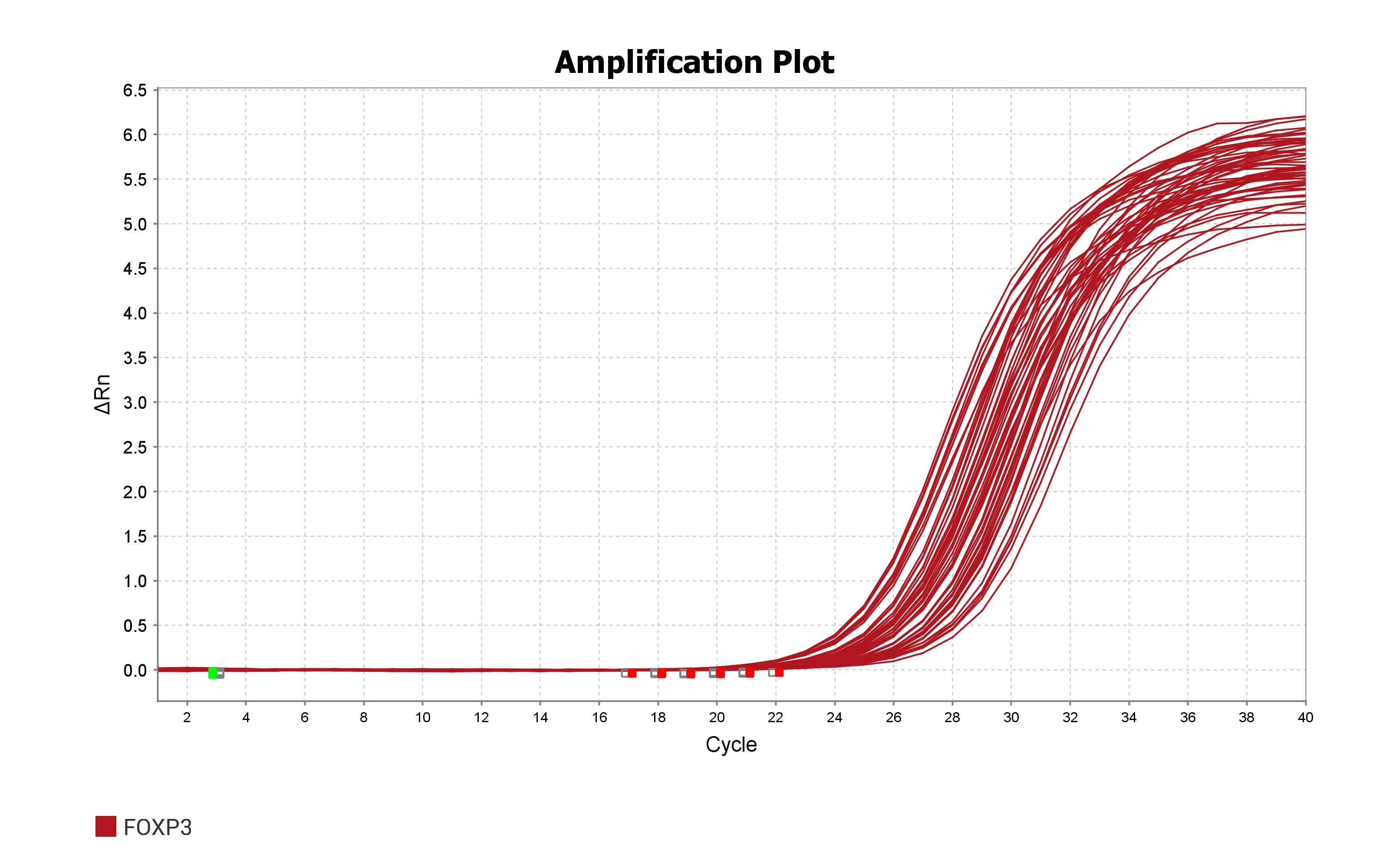

Supplement: Supplementary file 2 [file DataSheet2.zip › qRT-PCR-MZJ-2025-09/Curve/Amplification Plot lixinxin xibao FOXP3 .jpg]

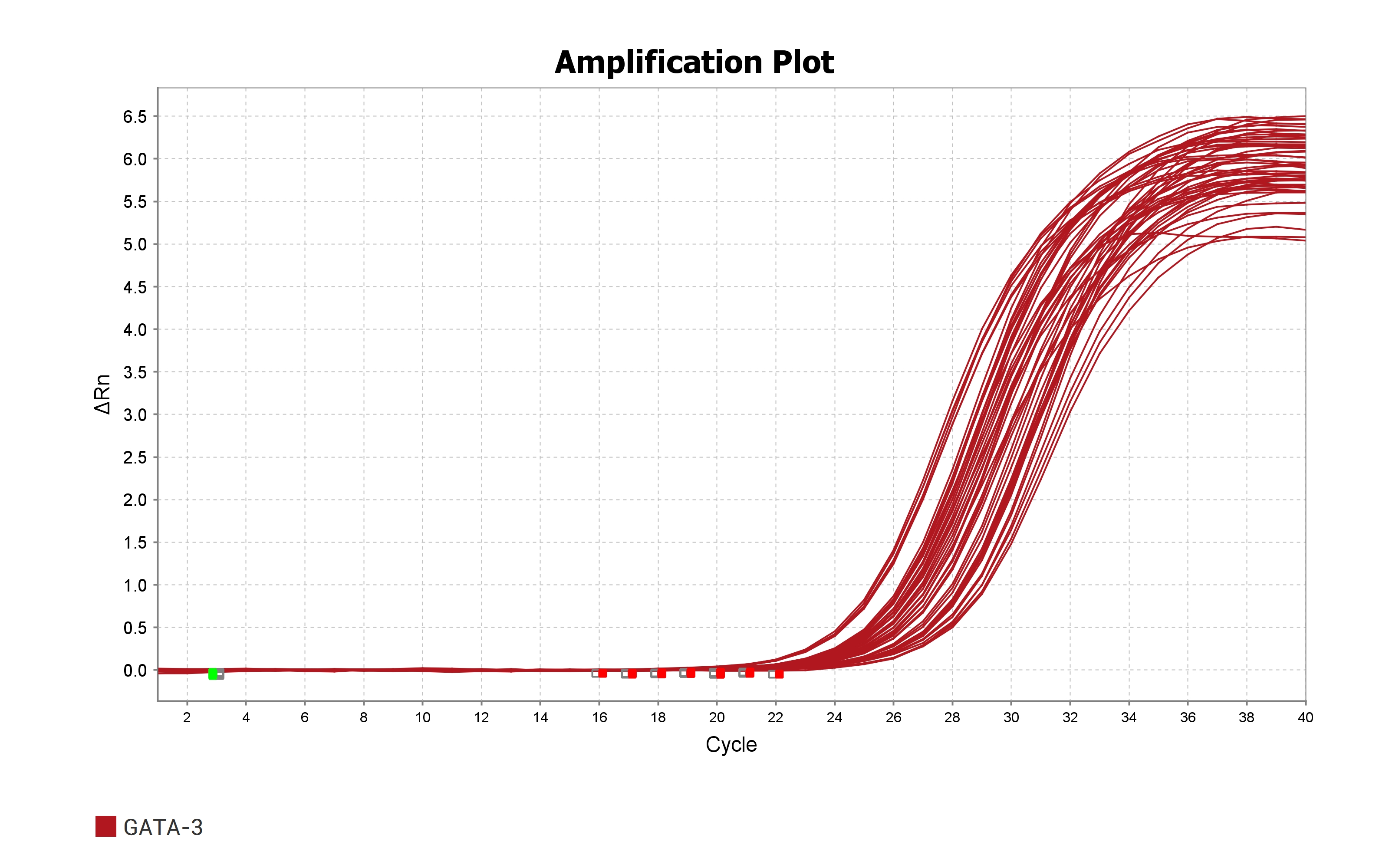

Supplement: Supplementary file 2 [file DataSheet2.zip › qRT-PCR-MZJ-2025-09/Curve/Amplification Plot lixinxin xibao GATA-3.jpg]

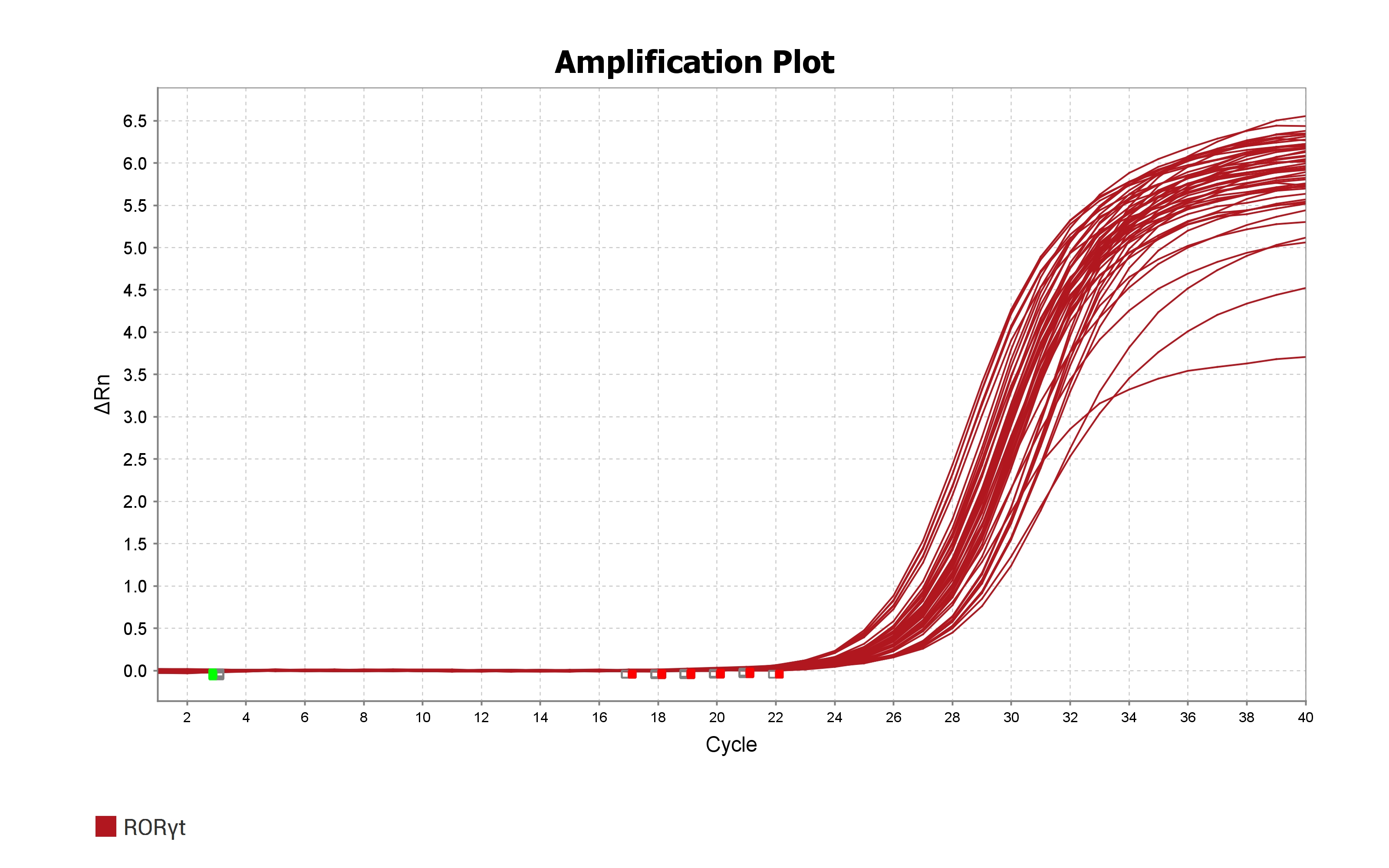

Supplement: Supplementary file 2 [file DataSheet2.zip › qRT-PCR-MZJ-2025-09/Curve/Amplification Plot lixinxin xibao RORyt .jpg]

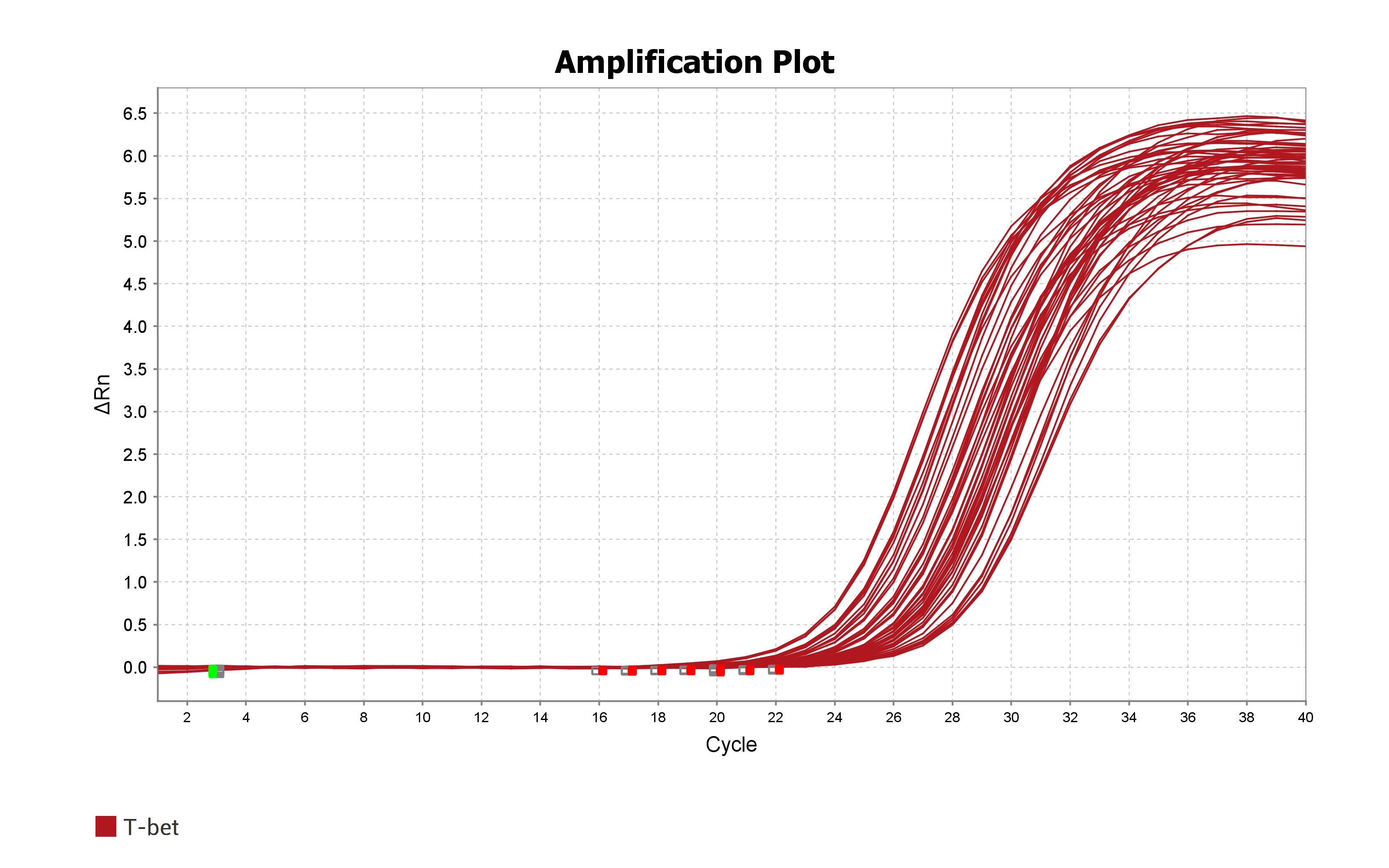

Supplement: Supplementary file 2 [file DataSheet2.zip › qRT-PCR-MZJ-2025-09/Curve/Amplification Plot lixinxin xibao T-bet .jpg]

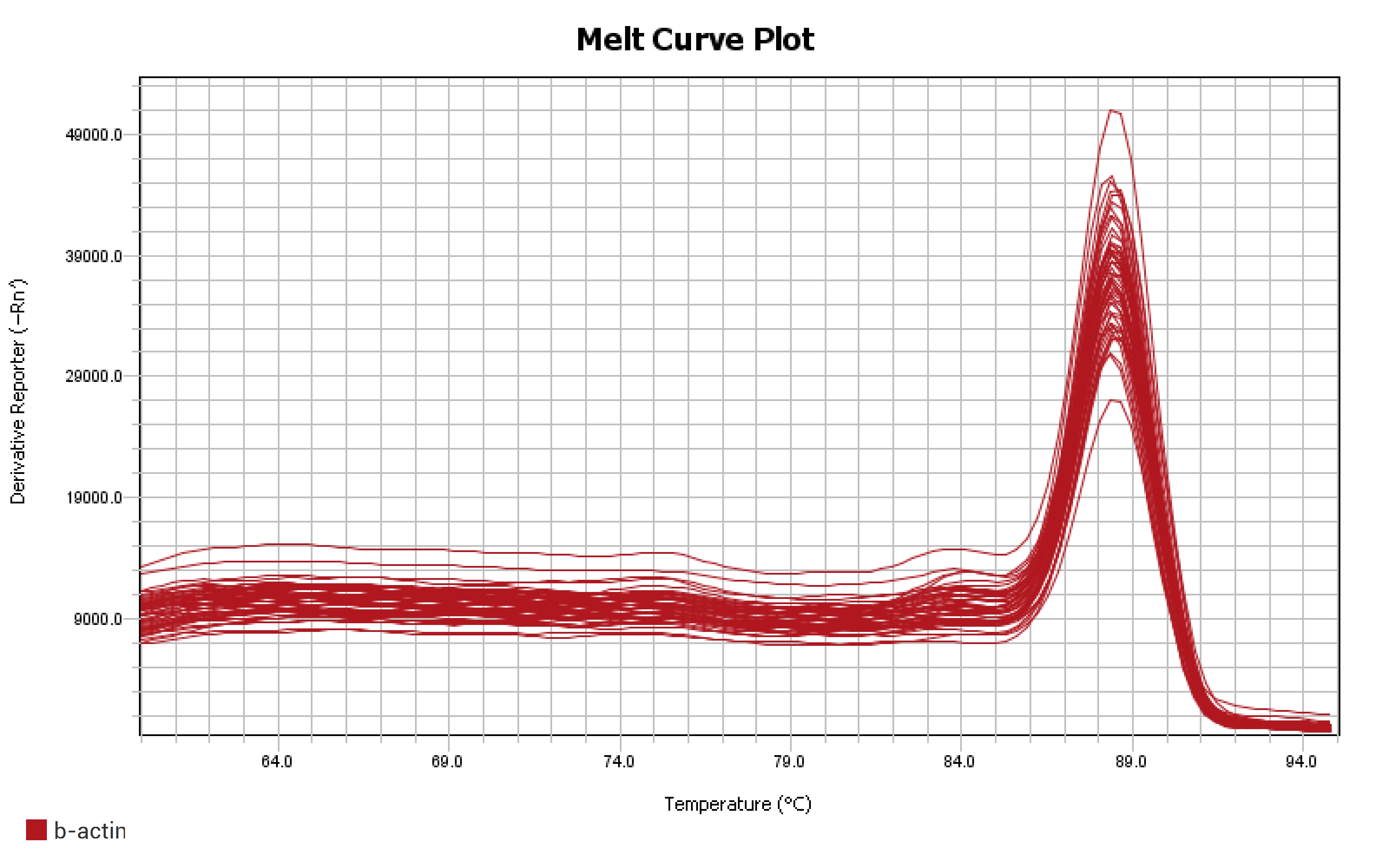

Supplement: Supplementary file 2 [file DataSheet2.zip › qRT-PCR-MZJ-2025-09/Curve/Melt Curve Plot lixinxin xibao b-actin.jpg]

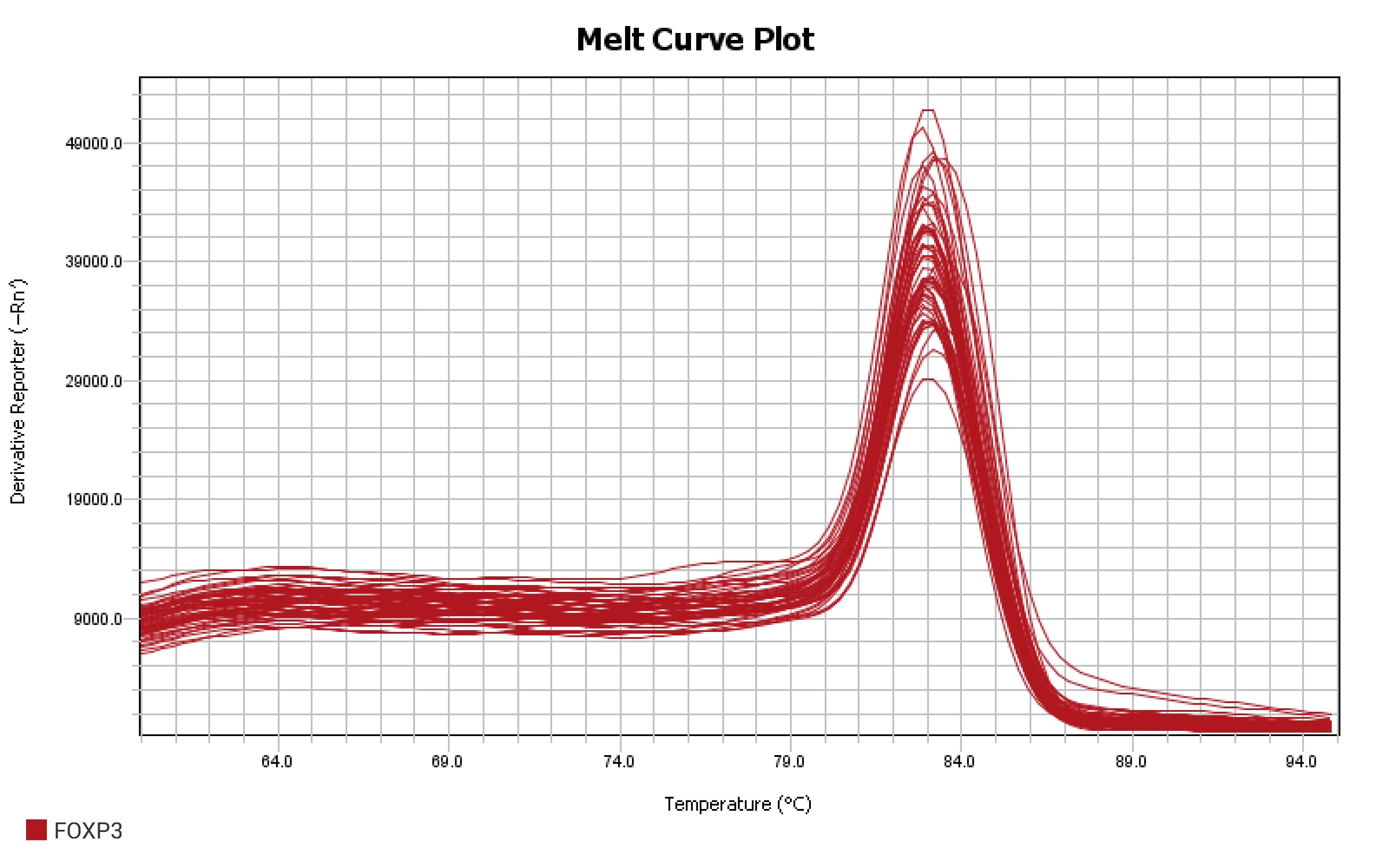

Supplement: Supplementary file 2 [file DataSheet2.zip › qRT-PCR-MZJ-2025-09/Curve/Melt Curve Plot lixinxin xibao FOXP3.jpg]

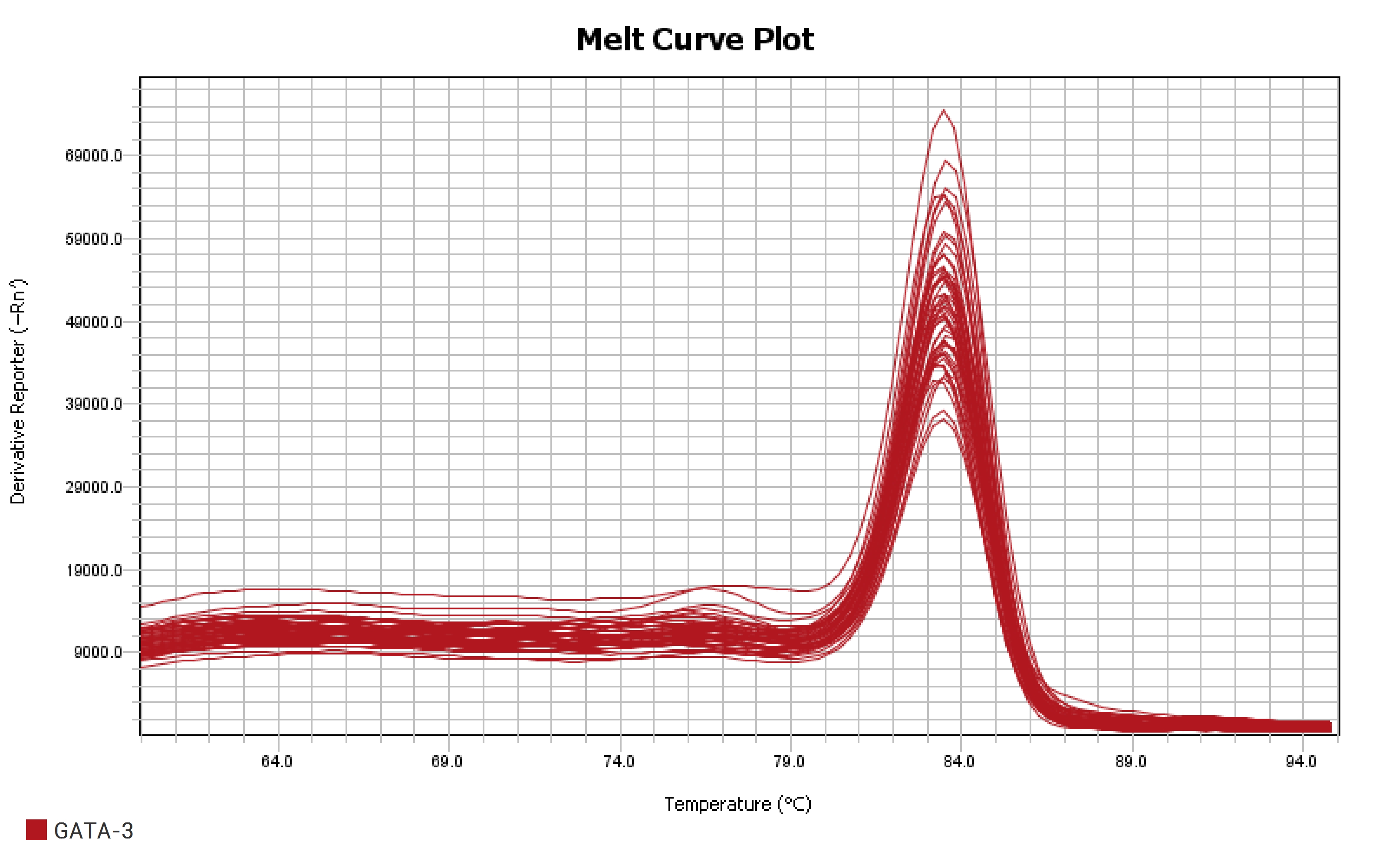

Supplement: Supplementary file 2 [file DataSheet2.zip › qRT-PCR-MZJ-2025-09/Curve/Melt Curve Plot lixinxin xibao GATA-3.jpg]

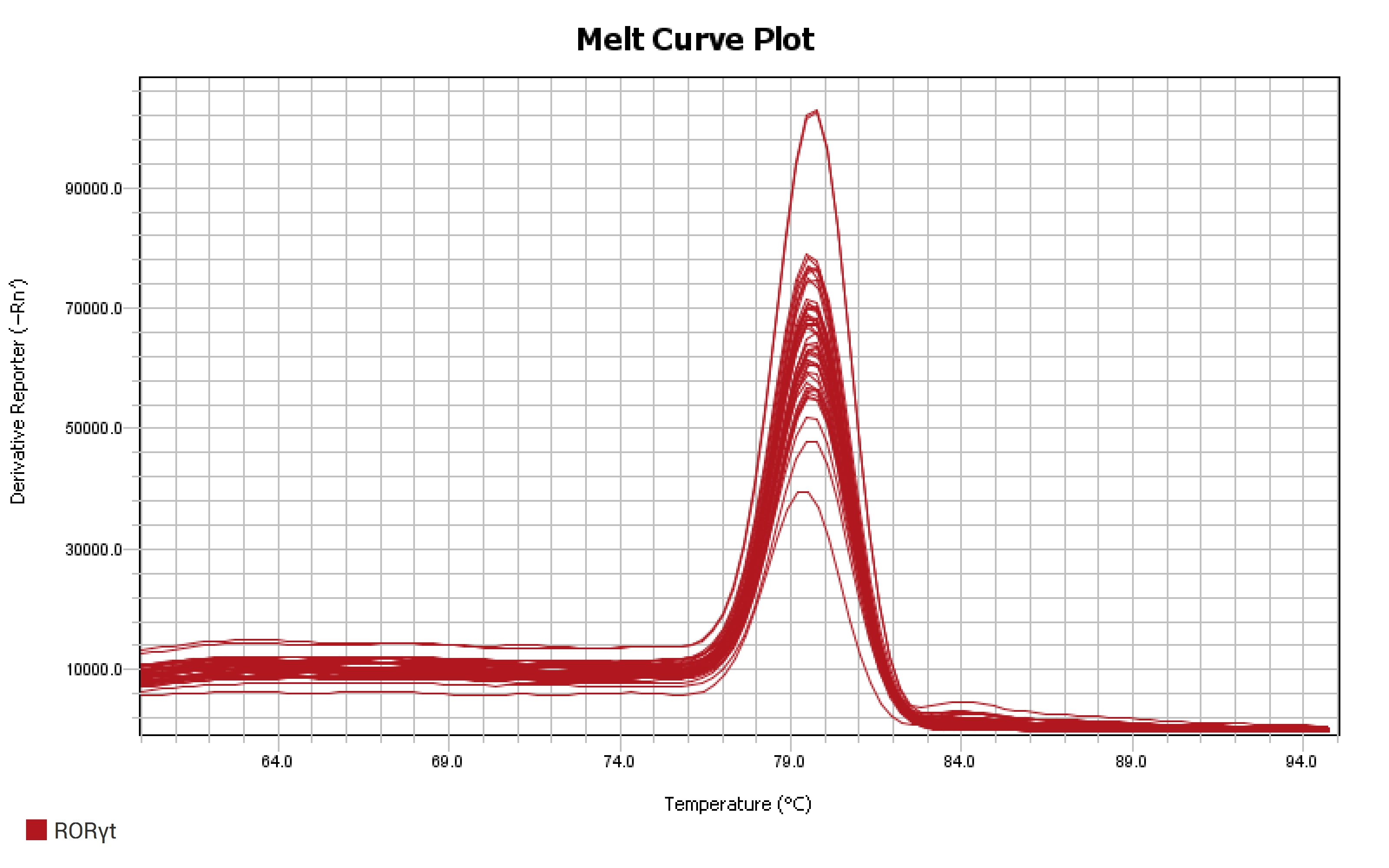

Supplement: Supplementary file 2 [file DataSheet2.zip › qRT-PCR-MZJ-2025-09/Curve/Melt Curve Plot lixinxin xibao RORyt.jpg]

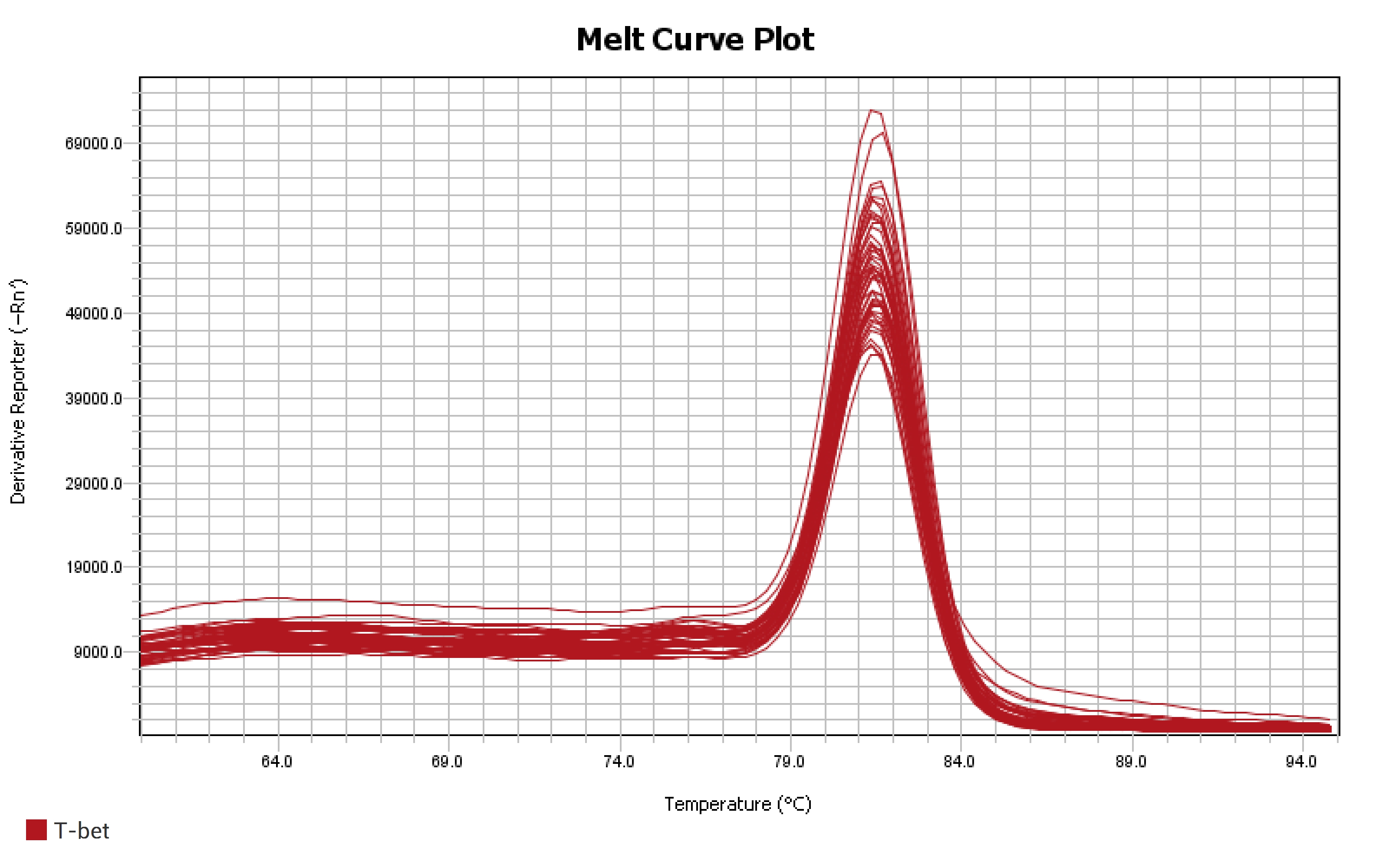

Supplement: Supplementary file 2 [file DataSheet2.zip › qRT-PCR-MZJ-2025-09/Curve/Melt Curve Plot lixinxin xibao T-bet .jpg]
